# Metabolic Targeting of Oxidative Phosphorylation Enhances Chemosensitivity in Triple-Negative Breast Cancer via a Synergistic Nanomedicine

**DOI:** 10.7150/thno.116250

**Published:** 2025-06-23

**Authors:** Xiaoding Shen, Hao Cai, Yihan Wang, Maodi Xie, Yunkun Li, Dayi Pan, Jing Jing, Qiyong Gong, Kui Luo

**Affiliations:** 1 Department of Radiology, Huaxi MR Research Center (HMRRC), Institution of Radiology and Medical Imaging, Rehabilitation Therapy, Breast Center, Institute of Breast Health Medicine, Department of Thoracic Surgery and Institute of Thoracic Oncology, Laboratory of Mitochondrial Metabolism and Perioperative Medicine, Frontiers Science Center for Disease-Related Molecular Network, State Key Laboratory of Biotherapy, West China Hospital, Sichuan University, Chengdu 610041, China.; 2 West China School of Medicine, Chengdu 610041, China.; 3 Functional and molecular imaging Key Laboratory of Sichuan Province, Key Laboratory of Transplant Engineering and Immunology, NHC, and Research Unit of Psychoradiology, Chinese Academy of Medical Sciences, Chengdu 610041, China.; 4 Xiamen Key Lab of Psychoradiology and Neuromodulation, Department of Radiology, West China Xiamen Hospital of Sichuan University, Xiamen 361021, China.

**Keywords:** oxidative phosphorylation, metabolic reprogramming, triple-negative breast cancer, chemosensitivity, drug delivery

## Abstract

**Rationale:** Triple-negative breast cancer (TNBC) is an aggressive form of breast cancer. There are very few targeted treatment options with satisfactory therapeutic indexes for TNBC. Although chemotherapy is the principal treatment modality for TNBC, its effectiveness is significantly compromised by low chemosensitivity in the TNBC patient population. Recent evidence has suggested that metabolic adaptation of tumor cells may play a critical role in reducing therapeutic responses. Metabolic interventions could enhance chemosensitivity and improve chemotherapeutic efficacy.

**Methods:** The influence of oxidative phosphorylation (OXPHOS) on TNBC chemosensitivity was evaluated by integrating bioinformatic analyses of patient datasets with metabolic phenotyping of TNBC cells. The correlation was established between the level of OXPHOS gene expression and therapeutic responses to standard chemotherapeutics. A mitochondria-targeting OXPHOS inhibitor, TPP-LND (a mitochondria-targeting derivative of lonidamine), was synthesized. A dendron-based polymer was conjugated with epirubicin (EPI) via an acid-responsive hydrazone bond to form a nanocarrier. TPP-LND was subsequently encapsulated into this nanocarrier, yielding PEG-Dendron-EPI@TPP-LND.

**Results:** In TCGA-BRCA cohorts, an elevation in OXPHOS gene expression was correlated with poor clinical outcomes and a higher IC_50_ value of chemotherapeutic drugs like EPI was found in the patients with upregulated OXPHOS expression, suggesting diminished chemosensitivity in these patients. TNBC cells heavily relied on mitochondrial ATP production, and TPP-LND effectively inhibited OXPHOS. PEG-Dendron-EPI@TPP-LND significantly suppressed tumor growth and prevented compensatory glycolytic activation without inducing observable systemic toxicity *in vivo*.

**Conclusion:** A mechanistic correlation was established between the OXPHOS activity and TNBC chemosensitivity. OXPHOS inhibition via TPP-LND was synergized with chemotherapy via the EPI prodrug to effectively suppress tumor growth and mitigate systemic toxicity of TPP-LND and EPI. This strategy could be promising for metabolic interventions to enhance the efficacy of chemotherapy in TNBC.

## Introduction

Triple-negative breast cancer (TNBC) remains the most aggressive breast cancer subtype with very few therapeutic options 1. Due to the lack of distinct biomolecular targets on TNBC cell surfaces, chemotherapy remains the cornerstone of systemic treatment recommended by clinical guidelines 2, 3. However, chemotherapy treatment for TNBC is often accompanied with suboptimal therapeutic indexes, which is primarily due to intrinsic heterogeneity in chemosensitivity among TNBC cells. This treatment approach for TNBC frequently leads to inconsistent treatment outcomes and high risks of relapse 4. Consequently, critical factors for modulating chemosensitivity of TNBC cells are actively examined 5, 6. While several molecules have been identified to regulate chemosensitivity in TNBC cells, distinct therapeutic targets for enhancing chemosensitivity remain to be elucidated 7-9. In this context, innovative strategies for discovering and harnessing novel therapeutic targets are currently under development to augment chemotherapeutic efficacy 10-15.

Recent studies have revealed a pivotal role of tumor metabolism in facilitating tumor progression by supplying vital nutrients, energy, and biosynthetic precursors to enable rapid proliferation, invasion, and metastasis of tumor cells 16-19. Moreover, metabolic adaptation of tumor cells significantly impedes the response of tumors to treatment 20. Under therapeutic stress, tumor cells undergo adaptive metabolic reprogramming, rewiring metabolic pathways, such as glutamine catabolism 21, 22, fatty acid oxidation 23, and nucleotide biosynthesis 24, to sustain proliferation 25, 26. Notably, energy metabolism, including glycolysis and oxidative phosphorylation (OXPHOS), is central for regulating these metabolic adaptations 27. Profiling the energy metabolism of TNBC cells could help identifying effective energy metabolic interventions to improve therapeutic outcomes of different modalities.

The Warburg effect, a long-standing paradigm in the field of cancer research, supports that cancer cells favor glycolysis over OXPHOS, even under an aerobic condition, to generate ATP and biosynthetic precursors 27, 28. However, emerging evidence reveals a dynamic interplay between glycolysis and OXPHOS 29. Tumor cells can flexibly reprogram their metabolic pathways to adapt to a dynamic tumor microenvironment 30. For instance, glucose deprivation, chemotherapeutic stress, or metastatic spread can trigger a metabolic shift toward OXPHOS to sustain energy production and maintain the redox balance 31-33. An increase in the OXPHOS activity has been mechanistically correlated with a compromised chemotherapy efficacy in multiple cancer types 34, 35. In colorectal cancer, SIRT1/PGC1α-mediated OXPHOS activation has been associated with alterations in cellular redox homeostasis and mitochondrial metabolism, diminishing the cytotoxic effect of 5-fluorouracil 36. Similarly, in prostate cancer, RB1 loss leads to an enhancement in mitochondrial biogenesis and an elevation in the OXPHOS activity, which has been shown to improve the effectiveness of targeted therapies 37. In TNBC, a subset of cells modulates treatment responses by facilitating pyruvate entry into the mitochondria, leading to changes in TCA cycle intermediates and enhancements in the reliance on OXPHOS 38. These metabolic changes have solidified the role of OXPHOS in the therapeutic response of TNBC to chemotherapy, confirming the clinical relevance of targeting OXPHOS to enhance the chemosensitivity of TNBC.

In this study, the influence of the OXPHOS activity on the chemosensitivity of TNBC was initially examined, and OXPHOS inhibition in synergy with chemotherapy was subsequently explored to enhance therapeutic outcomes. Bioinformatic interrogation of TCGA-BRCA cohorts revealed that an elevation in the OXPHOS gene expression was associated with poor clinical outcomes. Drug response modeling in breast cancer (BC) patients stratified by high and low oxidative phosphorylation scores suggested that higher OXPHOS activity was inversely correlated with the level of chemosensitivity. Predominant reliance on OXPHOS was seen after metabolic phenotyping of 4T1 murine TNBC cells in a stage IV disease model, and mitochondrial ATP production accounted for approximately 75% of total ATP—three times that from glycolysis. To therapeutically harness this metabolic characteristic, a mitochondria-targeting derivative of lonidamine (TPP-LND) was developed by conjugating lonidamine (LND) with triphenylphosphine group (TPP^+^). Mitochondrial accumulation of TPP-LND resulted in remarkable OXPHOS inhibition. To improve suboptimal pharmacokinetics of TPP-LND, a dendron-based polymer-EPI prodrug was prepared for delivery of hydrophobic TPP-LND. TPP-LND in the nanocarrier could mitigate compensatory glycolytic activation, while co-delivery of TPP-LND and EPI in one single nanocarrier resulted in a substantial enhancement in antitumor efficacy and a pronounced reduction in systemic toxicity of EPI and LND. In summary, our study shed light on the mechanistic correlation between OXPHOS activity and TNBC chemosensitivity, and this correlation was harnessed to develop a rational combinatorial strategy via a nanomedicine to enhance chemotherapeutic response by targeting tumor metabolism.

## Results

### The impact of OXPHOS levels on tumor treatment

**Figure 1:**
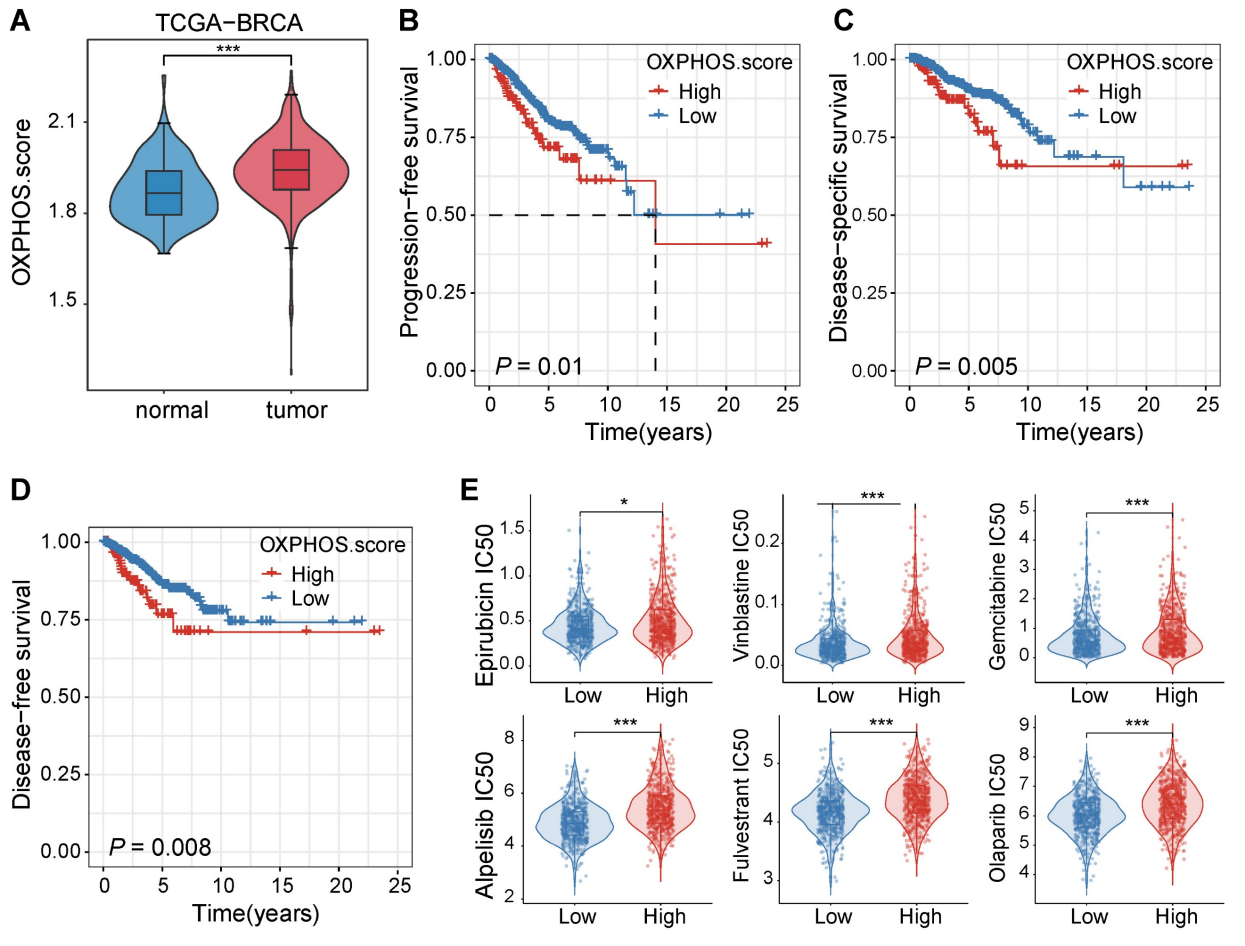
** Elevated OXPHOS activity in breast cancer was correlated with poor prognosis and reduced sensitivity to chemotherapeutic agents.** (A) A violin plot for OXPHOS scores of tumor tissues and their adjacent normal tissues from the TCGA-BRCA cohort. Tumor tissues displayed significantly higher OXPHOS scores (***P < 0.001). (B-D) Kaplan-Meier survival curves stratified by OXPHOS scores (high vs. low) within the same cohort. A high OXPHOS activity was well correlated with a shortened progression-free survival rate (PFS, B), a disease-specific survival rate (DSS, C), and a disease-free survival rate (DFS, D). (E) Drug response prediction analysis results via the OncoPredict R package. Breast cancer patients with elevated OXPHOS scores were often administrated with chemotherapeutic agents at a higher estimated IC_50_ value, including epirubicin, vinblastine, gemcitabine, cisplatin, alpelisib, and olaparib, indicating lower sensitivity of TNBC cells with higher OXPHOS scores to these chemotherapeutic agents (*P < 0.05, **P < 0.01, ***P < 0.001).

We initially examined the patient data from the TCGA-BRCA cohort to assess the impact of OXPHOS on breast cancer progression and treatment responses. The analysis revealed that OXPHOS-related gene expression was significantly elevated in tumor tissues compared to adjacent normal tissues (*P* < 0.001, Figure 1A), suggesting that mitochondrial metabolism was upregulated in breast cancer and it may play a role in tumor progression and therapeutic resistance.

Next, we assessed the clinical significance of the OXPHOS activity by comparing survival rates of patients with high and low levels of OXPHOS. Elevated OXPHOS activity was associated with significantly poorer prognosis, which was supported with shorter progression-free survival rates (PFS, *P* = 0.01, Figure 1B), reduced disease-specific survival rates (DSS, *P* = 0.005, Figure 1C), and lower disease-free survival rates (DFS, *P* = 0.008, Figure 1D). These findings suggested that patients with high OXPHOS activity may experience disease progression, recurrence, or breast cancer-specific mortality despite receiving standard treatments.

To reveal the underlying correlation between the OXPHOS level and the degree of drug sensitivity, we performed a computational drug response prediction analysis using the OncoPredict R package with training data. The differences in half-maximal inhibitory concentration (IC_50_) values of various chemotherapeutic drugs in BC patients with high and low OXPHOS scores were evaluated. Significant differences in the IC_50_ values were identified in the drugs including topoisomerase inhibitors (e.g., epirubicin, vinblastine), nucleotide analogues (e.g., gemcitabine), platinum-based agents (e.g., cisplatin), and targeted inhibitors such as the PI3K inhibitor alpelisib and the PARP inhibitor olaparib. Tumors with high OXPHOS activity were often treated with these drugs at a significantly higher IC_50_ value (*P* < 0.05 to *P* < 0.001, Figure 1E), consolidating that the metabolic activity of TNBC cells may contribute to reduced chemosensitivity and poor prognosis.

### TPP-LND inhibited OXPHOS and enhanced chemosensitivity

**Figure 2:**
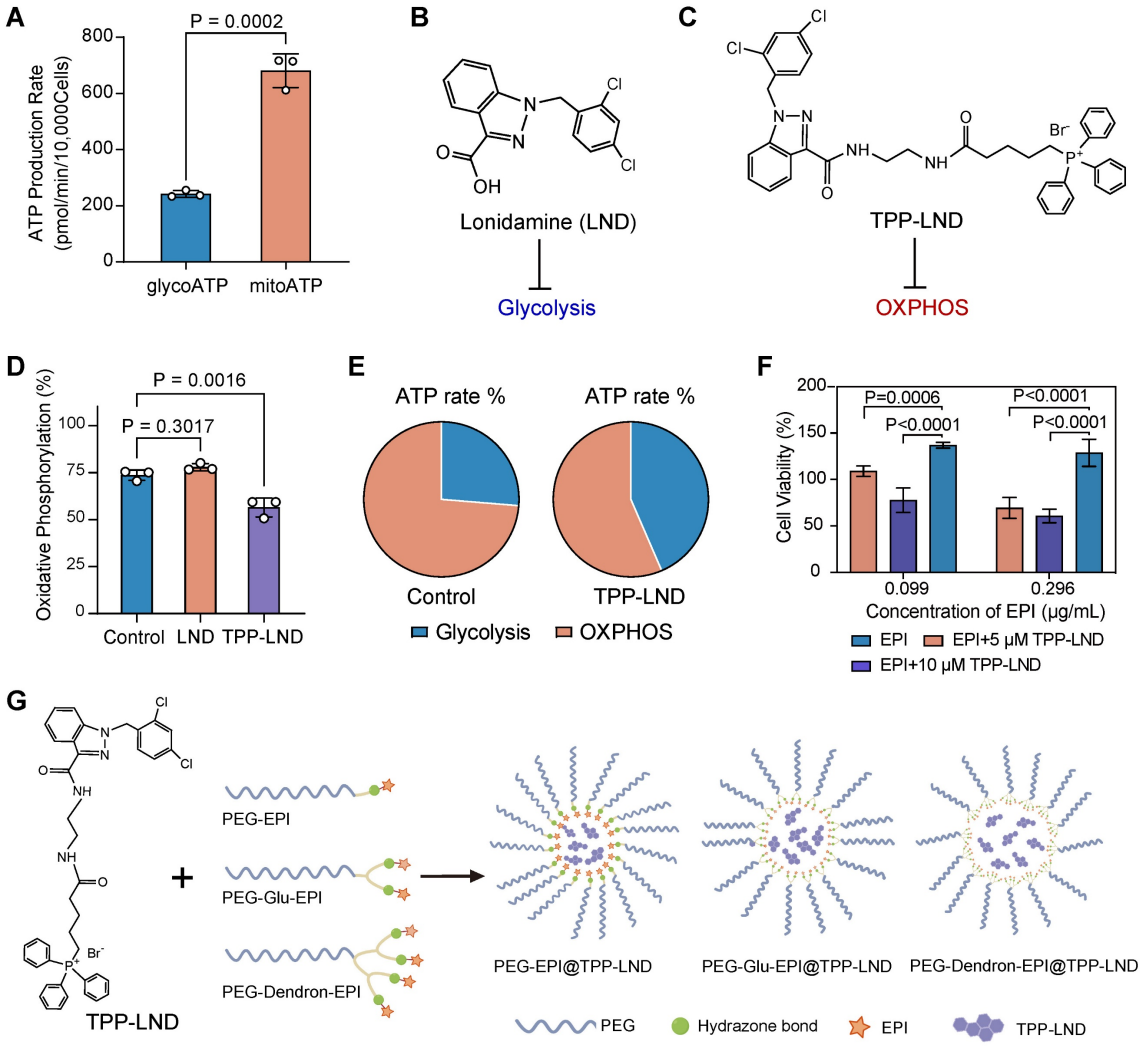
** Analysis of cellular metabolic preferences.** (A) The ATP production rate by 4T1 cells through glycolysis (glycoATP) and oxidative phosphorylation (mitoATP). (B) Chemical structure of LND. (C) Chemical structure of TPP-LND. (D) The degree of oxidative phosphorylation in 4T1 cells treated with LND and TPP-LND. (E) The proportion of ATP produced by glycolysis and oxidative phosphorylation in 4T1 cells treated with TPP-LND versus the control group. (F) Cell viabilities after treatment with the combination of EPI at an EPI concentration of 0.099 μg/mL or 0.296 μg/mL) and TPP-LND at a concentration of 5 μM or 10 μM. (G) Schematic illustration of PEG-EPI prodrugs and their self-assembly with encapsulated TPP-LND for co-delivery of EPI and TPP-LND. The chemical structures of PEG-EPI prodrugs are shown in Figure S27.

To systemically examine the role of OXPHOS in advanced TNBC, 4T1 cells, a highly metastatic stage IV TNBC model, were selected. The Seahorse energy metabolism phenotype assays revealed that ATP production in 4T1 cells was predominantly derived from mitochondrial oxidative phosphorylation (OXPHOS) rather than glycolysis (*P* = 0.0002, Figure 2A). This finding confirmed that advanced TNBC cells, such as 4T1 cells, were highly dependent on OXPHOS for energy metabolism, suggesting targeting OXPHOS could be a promosing therapeutic strategy.

To exploit the vulnerability of the metabolic process, we evaluated two metabolic inhibitors: LND, a commercially available glycolytic inhibitor (Figure 2B), and TPP-LND, a synthetic TPP-conjugated derivative of LND for mitochondrial targeting (Figure 2C). The synthesis route and structural characterization of TPP-LND are provided in the Supporting Information (Figure S1-S4). LND displayed no significant inhibitory effect on OXPHOS, on the contrast, TPP-LND exhibited a potent inhibitory effect on mitochondrial OXPHOS in 4T1 cells, leading to a notable reduction in OXPHOS-dependent ATP production (*P* = 0.0016, Figure 2D-2E). These results indicated that TPP-LND could effectively interfere with mitochondrial metabolism, thus it could be a promising candidate for targeting OXPHOS in TNBC cells.

Given the strong correlation between an elevated OXPHOS level and a reduced degree of chemosensitivity observed in our drug sensitivity analysis (Figure 1E), we investigated the impact of OXPHOS inhibition by TPP-LND on cytotoxic effects of chemotherapy. EPI, a commonly used chemotherapeutic agent for breast cancer, was selected for this study. Treatment of 4T1 cells with TPP-LND significantly enhanced cytotoxic effects of EPI, even at a low dose (*P* < 0.0001, Figure 2F, Figure S5). Furthermore, a higher concentration of TPP-LND were associated with a more pronounced cytotoxic effect of EPI, suggesting the enhancement in the EPI efficacy was dependent on the TPP-LND dose. TPP-LND alone did not display significant cytotoxicity to cells at a concentration below 16.4 µM for 48 h, and cell viability exceeding 100% was observed after the TPP-LND treatment (Figure S6). However, it significantly enhanced the cytotoxicity of EPI. These results validated our hypothesis that OXPHOS inhibition may enhance chemosensitivity in TNBC, providing a novel therapeutic approach to improving therapeutic outcomes by combining metabolic inhibitors with chemotherapeutic agents. Therefore, the aim of this study was to use TPP-LND as an OXPHOS inhibitor in TNBC cells to enhance the chemosensitivity of EPI. Due to the hydrophobic nature of both TPP-LND and EPI, we developed delivery vehicles to facilitate the *in vivo* application of both drugs. EPI contains a site amenable to chemical modification, allowing the design and synthesis of three amphiphilic PEG-EPI with different structures as EPI prodrugs: PEG-EPI, PEG-Glu-EPI, and PEG-Dendron-EPI (Figure 2G). In contrast, TPP-LND does not have chemically modifiable sites; thus, it could be physically encapsulated in the self-assembled nanoparticles formed by the EPI prodrugs.

### Design of PEG-EPI conjugates as prodrugs

**Figure 3:**
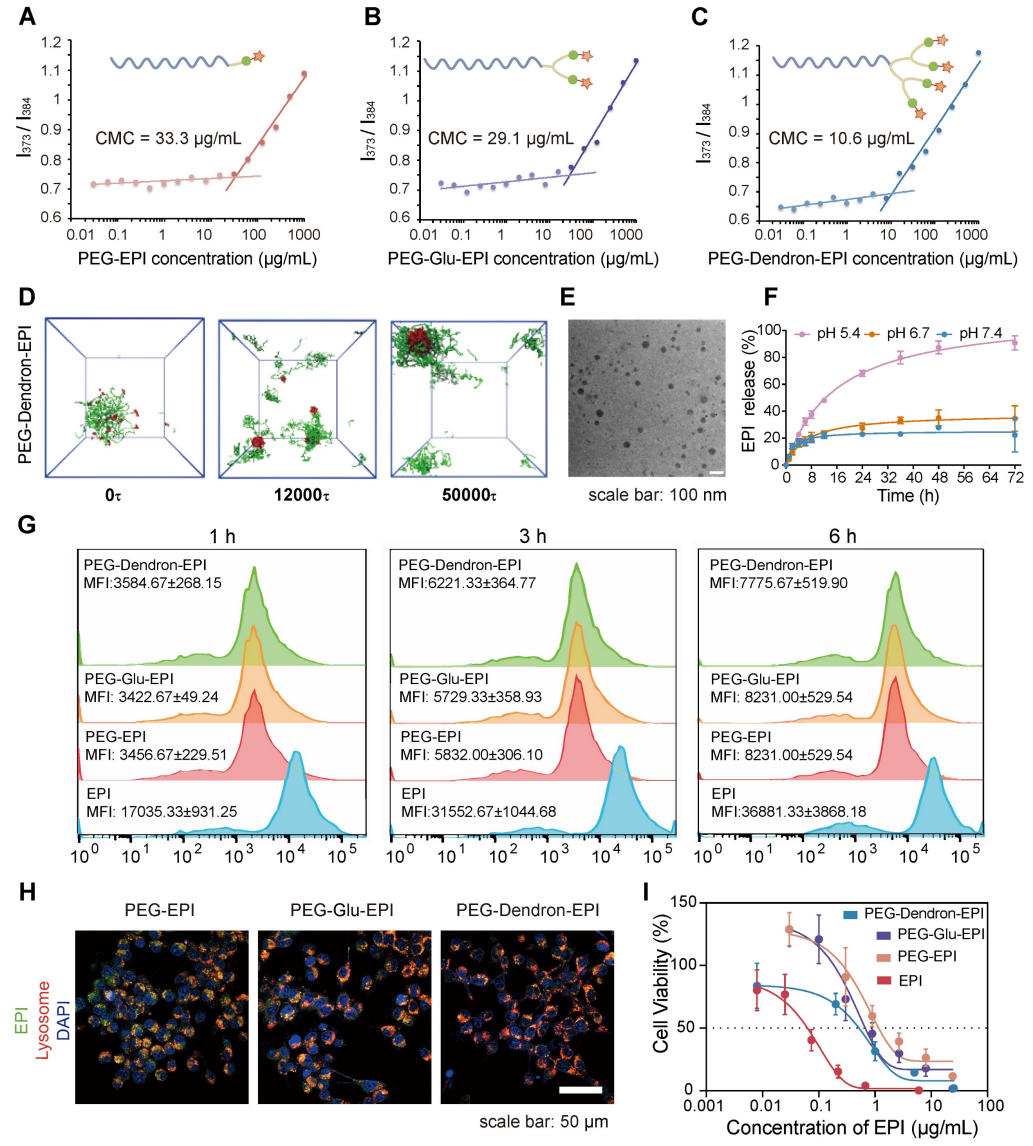
** Physicochemical properties and biological effects of PEG-EPI prodrugs.** (A-C) The critical micelle concentration (CMC) of PEG-EPI (A), PEG-Glu-EPI (B) and PEG-Dendron-EPI (C) measured using pyrene as a fluorescent probe. (D) DPD simulation of the self-assembly process of PEG-Dendron-EPI to form aggregates in an aqueous environment. (E) TEM images of PEG-Dendron-EPI (scale bar = 100 nm). (F) The EPI release curves under different pH conditions. (G) Flow cytometry analysis of cellular uptake of PEG-EPI, PEG-Glu-EPI, and PEG-Dendron-EPI at 1 h, 3 h, and 6 h. (H) Fluorescence confocal microscopy images for the localization of PEG-EPI, PEG-Glu-EPI, and PEG-Dendron-EPI in organelles at 6 h (EPI incubation concentration: 10 μg/mL, green: EPI, red: Lysosome, blue: DAPI, scale bar: 50 μm). (I) Toxic effects of PEG-EPI, PEG-Glu-EPI, and PEG-Dendron-EPI on 4T1 cells after 48 h of treatment.

The design and preparation of the three PEG-EPI prodrugs: PEG-EPI, PEG-Glu-EPI, and PEG-Dendron-EPI, were shown in Scheme S2-S4 and Figure S7-S29. Due to the presence of hydrophilic PEG chains and hydrophobic EPI segments in the PEG-EPI prodrugs, they could self-assemble into nanoparticles in an aqueous solution, and the particle size was relatively stable (Figure S30). Notably, the critical micelle concentration (CMC) analysis revealed that PEG-Dendron-EPI exhibited the lowest CMC value compared to the other two prodrugs, suggesting its superior self-assembly capability (Figure 3A-C). Additionally, the self-assembly dynamics of the three polymer prodrugs in an aqueous environment were investigated using dissipative particle dynamics (DPD) simulations. As shown in Figure 3D and Figure S31, the hydrophilic and hydrophobic segments of the polymers in a random distribution pattern were transitioned to an assembled structure with a hydrophobic core and hydrophilic coverage on its surface, and the assembled structure gradually turned into stable nanoparticles. Notably, among the three EPI prodrugs, those bearing a higher number of terminal EPI groups formed nanostructures with larger hydrophobic cores. Driven by π-π stacking and hydrophobic interactions, these enlarged hydrophobic domains are expected to endow the nanoparticles with improved self-assembly capability and enhanced colloidal stability. The hydrodynamic particle size and the assembly morphology of three EPI prodrugs were investigated by DLS and transmission electron microscopy (TEM), respectively. Self-assembly of three EPI prodrugs in water resulted in an average hydrodynamic size of 175.26 nm, 148.88 nm, and 122.62 nm, respectively (Table S1). Under the TEM, the nanoparticles formed from three EPI prodrugs exhibited a spherical morphology with a diameter of approximately 50 nm and they were relatively regular in shape (Figure 3E, Figure S32). It is noteworthy that the size observed under the TEM was smaller than the hydrodynamic particle size measured by DLS. This discrepancy could be attributed to a slightly contracted structure in a solid state for TEM samples compared to DLS samples in an aqueous environment.

In the prodrug structure, EPI was conjugated to the PEG terminal via a pH-sensitive hydrazone bond. We simulated a weakly acidic tumor microenvironment to assess the release of EPI from three EPI prodrugs under different pH conditions. Three EPI prodrugs exhibited a pH-responsive drug release pattern. At pH 7.4, a small amount of EPI was released from these prodrugs and the cumulative release was approximately 20% over 72 h. As the pH in the release environment decreased, the cumulative release of EPI from these prodrugs increased. Notably, at pH 5.4, sustained release behavior of EPI was observed in these prodrugs. The cumulative EPI release reached 80.7 ± 0.7% and 77.7 ± 4.8% from PEG-EPI and PEG-Glu-EPI, respectively, over 72 h (Figure S33), by contrast, a slightly higher cumulative release of 90.8 ± 5.2% was achieved from PEG-Dendron-EPI (Figure 3F).

The localization of three EPI prodrugs in 4T1 cells after their uptake was observed under the CLSM. PEG-EPI, PEG-Glu-EPI, and PEG-Dendron-EPI inside 4T1 cells exhibited comparable fluorescence intensities. Similar intracellular location was observed for three prodrugs and the majority of them were found inside the lysosome (Figure 3G-H, Figure S34-S35). Subsequent flow cytometry analysis revealed that there was no significant difference in the uptake amount of three EPI prodrugs after exposure to 4T1 cells for 1 h, 3 h, and 6 h. The internalization of the three acid-responsive EPI prodrugs by the cells was realized through the process of endocytosis. The acid-responsive bonds in the prodrugs were effectively cleaved at a low pH (5.4) within the lysosome. The cytotoxic effects of three EPI prodrugs on 4T1 cells were assessed using the CCK-8 assay. The results (Figure 3I) suggested that the three prodrugs exhibited a comparable level of cytotoxicity: a distinctive inhibitory effect of the EPI prodrugs on tumor cells. Among these EPI prodrugs, PEG-Dendron-EPI had the lowest IC_50_ value, and it displayed the most pronounced cytotoxic effect at the lowest drug concentration.

### Biodistribution of three EPI prodrugs and *ex vivo* imaging

**Figure 4:**
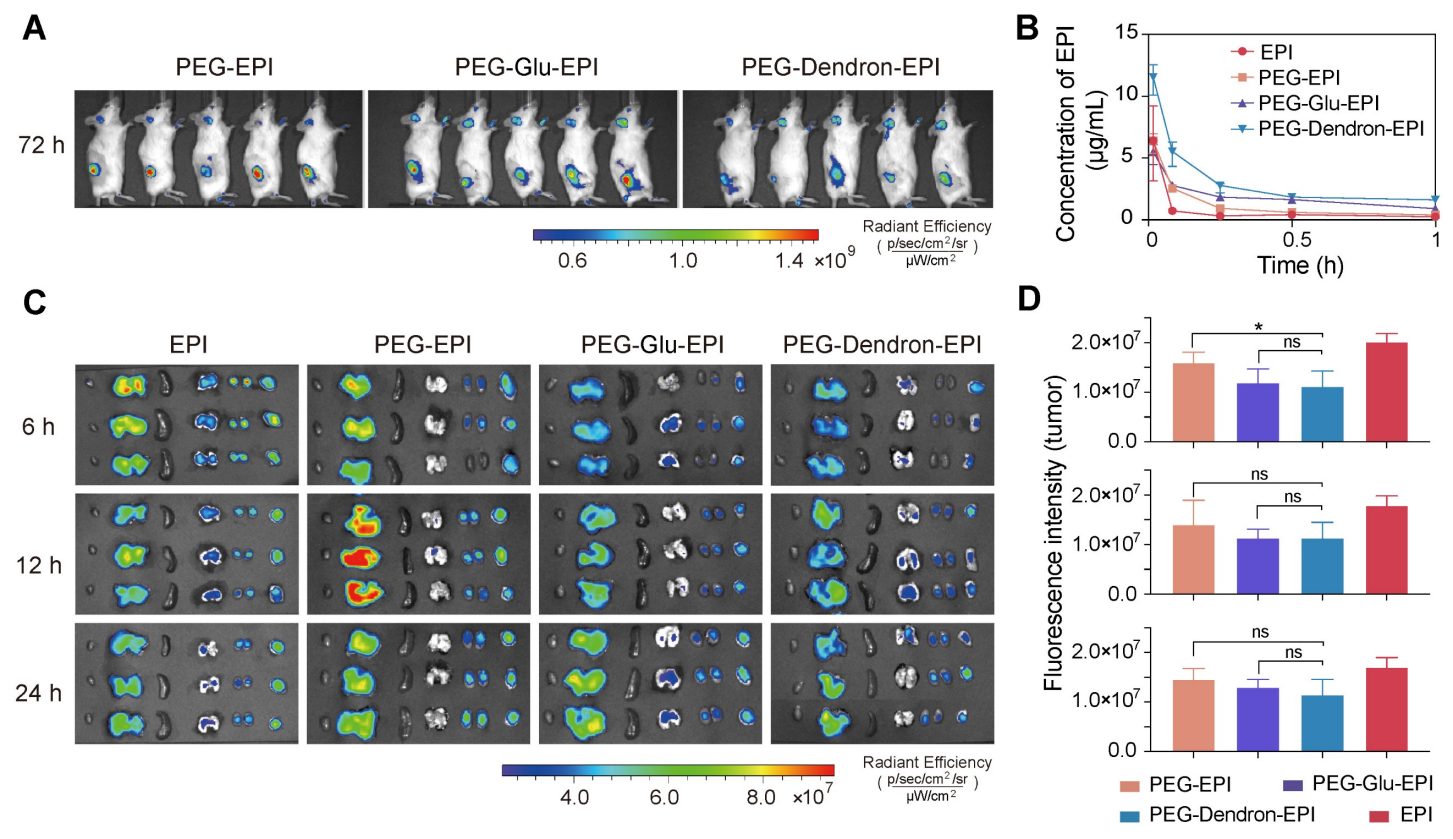
***In vivo* biodistribution and pharmacokinetics of PEG-EPI prodrugs.** (A) *In vivo* fluorescence images of tumor-bearing mice at 72 h post intravenous administration of Cy5-labeled nanoparticles based on PEG-EPI, PEG-Glu-EPI, and PEG-Dendron-EPI (Cy5 channel). (B) Pharmacokinetic profiles of EPI, PEG-Glu-EPI, and PEG-Dendron-EPI over 1 h after systemic injection. The peak concentration reached at one minute followed by a rapid decline (Ex: 480 nm, Em: 595 nm). (C) *Ex vivo* fluorescence images of excised tumors and major organs at 6, 12, and 24 h after injection (EPI channel). (D) Quantification of the fluorescence intensity in tumor tissues for each treatment group at different time points. Data are presented as mean ± standard deviation. All animals received an equivalent EPI dose of 8 mg/kg.

To investigate the biodistribution and pharmacokinetics of different EPI prodrugs, PEG-EPI, PEG-Glu-EPI, and PEG-Dendron-EPI were administered to tumor-bearing mice at an equivalent EPI dose of 8 mg/kg. *In vivo* imaging of mice using Cy5-labeled nanoparticles revealed detectable accumulation of three prodrug-based nanoparticles in the tumor area at 72 h post-injection (Figure 4A, Figure S36-S37). While these nanoparticles displayed fluorescent signal localized in the tumor region over 72 h, the signal intensity was observed to decrease at 3 h post-administration, suggesting they may be subjected to progressive systemic clearance, for example, renal excretion. Pharmacokinetic profiling based on the fluorescence signal of EPI revealed that all prodrug-based nanoparticles rapidly reached a peak in the blood concentration within 1 min post intravenous administration (Figure 4B). Notably, PEG-Dendron-EPI displayed a higher plasma concentration over the first hour compared to PEG-EPI and PEG-Glu-EPI, suggesting it had better circulation stability. After 1 h post-injection, the EPI concentration in the groups treated with EPI prodrugs dropped below 5 μg/mL (Figure S38, Table S2). *Ex vivo* fluorescence imaging (Figure 4C) at 6, 12, and 24 h post-injection confirmed that PEG-EPI accumulated predominantly in the liver at 12 h, and PEG-Glu-EPI and PEG-Dendron-EPI exhibited similar biodistribution profiles with prolonged retention in the tumor tissue. However, there was no statistically significant difference in the tumor fluorescence intensity at each time point (Figure 4D) among experimental groups treated by three prodrug groups. PEG-EPI was found to display a slightly higher accumulation level at 6 h compared to the other two prodrugs. These results suggested that dendronized PEG-EPI prodrugs exhibited favorable pharmacokinetics and great tumor retention, which may be due to an optimal particle size, PEGylation, and enhanced systemic stability, thus their *in vivo* therapeutic performance could be significantly improved.

### Synergistic effects of combined TPP-LND and EPI by co-delivery

**Figure 5:**
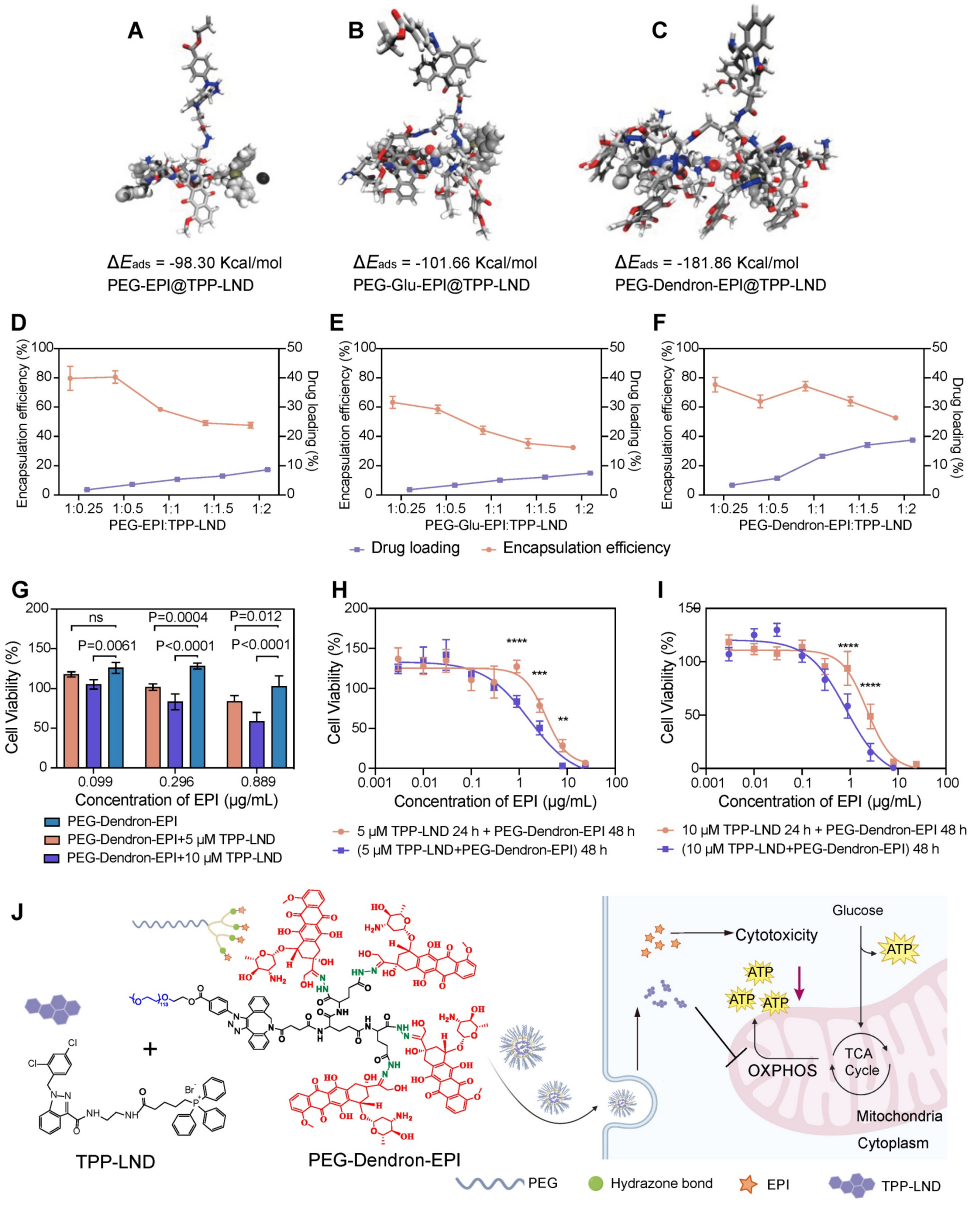
** Efficient encapsulation and co-delivery of TPP-LND by PEG-Dendron-EPI to achieve synergistic cytotoxicity.** (A-C) Molecular dynamics simulations of the binding interaction between TPP-LND and three PEG-EPI prodrugs with estimated adsorption energy (E_ads_) values: PEG-EPI (A), PEG-Glu-EPI (B), and PEG-Dendron-EPI (C). A more negative ΔE_ads_ indicated a stronger binding affinity. Drug loading contents and encapsulation efficiencies of TPP-LND in PEG-EPI (D), PEG-Glu-EPI (E) and PEG-Dendron-EPI (F) prodrugs at different loading ratios. (G) Cytotoxicity of PEG-Dendron-EPI in combination with TPP-LND (5 μM or 10 μM) at three equivalent EPI doses (0.099 μg/mL, 0.296 μg/mL, 0.889 μg/mL) against 4T1 cells at 48 h post treatment (n = 5). (H-I) Cytotoxicity of TPP-LND (H, 5 μM or I, 10 μM) and PEG-Dendron-EPI after simultaneous treatment of both formulations for 48 h versus sequential treatment by pretreating cells with TPP-LND for 24 h followed by PEG-Dendron-EPI for an additional 24 h. A reduction in the efficacy was seen during the sequential treatment compared to simultaneous treatment. (J) Schematic representation of the structural composition and mechanisms of action of the synergistic nanomedicine PEG-Dendron-EPI@TPP-LND. The co-delivery and release of both drugs inside TNBC cells could enhance the cytotoxic effect on TNBC.

TPP-LND is a mitochondria-targeting OXPHOS inhibitor. Since TPP-LND is hydrophobic and there are very few options for chemical modification of TPP-LND, physical encapsulation of TPP-LND was explored to combine TPP-LND and EPI for their synergistic effects. To evaluate the encapsulation efficiency of TPP-LND by PEG-EPI prodrugs, all-atom molecular dynamics (AAMD) simulations were performed to model the interaction between TPP-LND and EPI prodrugs including PEG-EPI, PEG-Glu-EPI, and PEG-Dendron-EPI constructs. The adsorption energies (E_ads_) were estimated to be -98.30 Kcal/mol, -101.66 Kcal/mol and -181.86 Kcal/mol for PEG-EPI@TPP-LND, PEG-Glu-EPI@TPP-LND, and PEG-Dendron-EPI@TPP-LND, respectively (Figure 5A-C, Table S3). Negative adsorption energies between TPP-LND and EPI prodrugs indicated they had strong binding affinity, thus a high encapsulation efficiency could be obtained due to strong hydrophobic interaction between TPP-LND and the dendronized EPI prodrug (Figure S39). Additionally, the DPD simulation results for the encapsulation of TPP-LND by polymer prodrugs (Figure S40-S42) revealed that hydrophobic TPP-LND, initially uniformly dispersed in an aqueous phase, gradually became aggregated and eventually were encapsulated into the hydrophobic core of the nanoparticles. This process manifested that the drug molecules first aggregated into small clusters in the aqueous phase, and the aggregates were subsequently incorporated into the interior of the nanoparticle upon contact with the polymer prodrug-based nanoparticles. Based on the findings of the simulation of the self-assembly of polymer prodrugs and the all-atom simulation, a higher EPI content promoted the formation of a larger hydrophobic core with a more negative adsorption energy for the drug that enables faster and more stable encapsulation of hydrophobic drugs. AAMD simulations supported that the interaction energy between PEG-EPI prodrugs and TPP-LND increased with a higher EPI content, indicating that a greater EPI loading enhanced the drug encapsulation efficiency. Meanwhile, DPD simulations of the micelle formation process and the resulting structural features at varying EPI concentrations corroborated the AAMD findings.

To evaluate encapsulation efficiency of TPP-LND by PEG-EPI prodrugs, the film method was applied to encapsulate TPP-LND by the PEG-EPI prodrug. In agreement with kinetic simulation results, the best encapsulation performance, in terms of encapsulation efficiency and drug loading capacity for TPP-LND, was achieved by the PEG-Dendron-EPI prodrug compared to PEG-EPI and PEG-Glu-EPI. The optimal encapsulation efficiency was seen at a 1:1 mass ratio of PEG-Dendron-EPI to TPP-LND, and a drug loading capacity of 13.2% was obtained (Figure 5D-5F, Figure S43 and Table S4-S6). From the encapsulation results, the PEG-Dendron-EPI prodrug, was selected for encapsulation of TPP-LND, and a novel nanomedicine, PEG-Dendron-EPI@TPP-LND, was generated from the encapsulation process at an optimal ratio.

The synergistic therapeutic effect of TPP-LND and PEG-Dendron-EPI was subsequently assessed *in vitro*. The dose of TPP-LND was fixed at two concentrations of 5 μM or 10 μM with no significant cytotoxicity, and a diluted dose of PEG-Dendron-EPI was applied. Significantly reduced cell viability was seen in the PEG-Dendron-EPI-treated group even at TPP-LND concentrations with low cytotoxicity (Figure 5G, Figure S44). Furthermore, the inhibitory effect of 10 μM TPP-LND combined with PEG-Dendron-EPI on 4T1 cell growth was more significant than that of 5 μM TPP-LND. The inhibitory effect on 4T1 cells was also assessed after sequential treatment with two agents. 4T1 cells were initially treated with TPP-LND at a concentration of 5 μM or 10 μM for 24 h, and subsequently with PEG-Dendron-EPI for additional 24 h or 48 h. Interestingly, sequential treatment with TPP-LND and then PEG-Dendron-EPI was not effective in inhibiting 4T1 cell growth compared to simultaneous treatment with TPP-LND and PEG-Dendron-EPI for 48 h (Figure 5H-5I). Consequently, a strategy of co-delivering TPP-LND and PEG-Dendron-EPI was implemented by physically loading TPP-LND into PEG-Dendron-EPI self-assembled nanoparticles (Figure 5J). This approach could facilitate the formation of a synergistic nanomedicine, enabling simultaneous exertion of the effects of both agents to achieve an effective antitumor effect.

### Co-delivery of TPP-LND and EPI reprogrammed metabolism of TNBC cells by inhibiting mitochondrial OXPHOS

**Figure 6:**
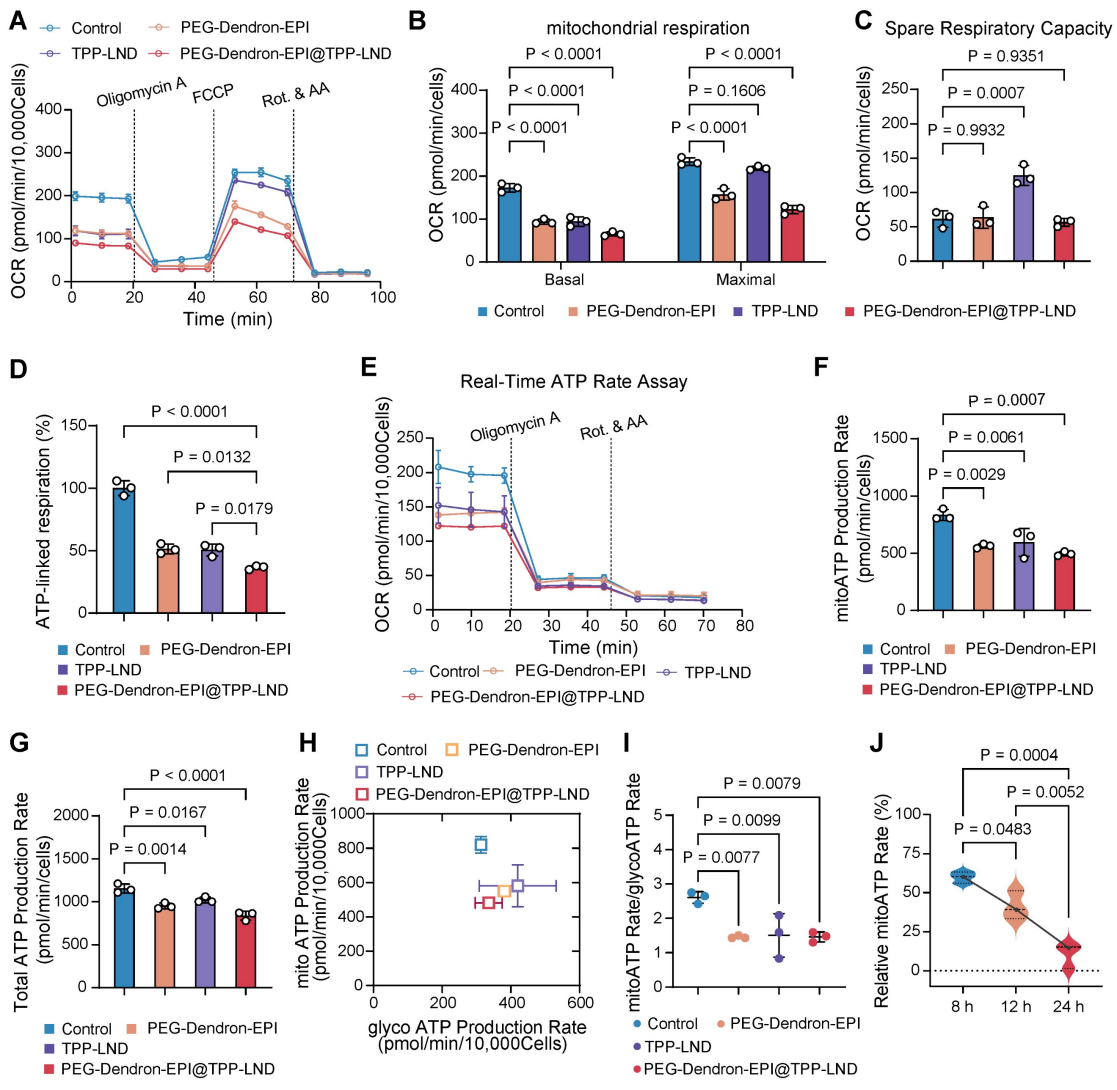
**The PEG-Dendron-EPI@TPP-LND nanomedicine reprogramed tumor cell metabolism by inhibiting mitochondrial OXPHOS. (**A) Oxygen consumption rates (OCRs) of 4T1 cells treated with PEG-Dendron-EPI@TPP-LND, PEG-Dendron-EPI, TPP-LND or the control for 12 h. Mitochondrial stress was applied to obtain kinetic OCR curves. (B) Basal and maximal OCR levels of treated cells compared to the control. (C) The spare respiratory capacity of treated cells compared to the control cells. (D) The rate of ATP production in cells exposed to different treatments vs the control group, which was set to 100% according to the original ATP-linked respiration data in Figure S46. (E) Kinetic OCR curves derived from the real-time ATP production rate assay in treated cells and control cells. (F) Mitochondrial ATP (mitoATP) production rates in 4T1 cells after treatment with different formulations. (G) Total ATP production rates in different treatment groups after combining the contributions of glycolytic and mitochondrial ATP synthesis. (H) ATP production rates from mitochondrial oxidative phosphorylation versus ATP production rates from glycolysis, which could be used to determine energy metabolic phenotypes of 4T1 cells to meet cellular energy balance after treatment with different formulations. (I) ATP rate indexes in different treatment groups, which was calculated from the ratio of mitoATP to glycolytic ATP (glycoATP). The ATP rate index could be used for identifying a shift in the metabolic phenotype after treatment with different formulations. (J) mitoATP production rates of 4T1 cells at 8, 12, and 24 h post treatment with PEG-Dendron-EPI@TPP-LND. mitoATP production rates were set as 100% at each time point in the control cells. Data are shown as mean ± S.D.; n = 3 biologically independent samples.

We have confirmed that 4T1 cells, a representative model of highly metastatic TNBC, displayed a metabolic phenotype characterized with enhanced OXPHOS activity. Notably, the proportion of ATP generated via mitochondrial oxygen consumption was over three times greater than that produced through glycolysis (Figure S45). Targeting OXPHOS by PEG-Dendron-EPI@TPP-LND could be a promising therapeutic strategy for this cancer subtype. First, we assessed the impact on the oxygen consumption rate (OCR) of 4T1 cells after treatment with the PEG-Dendron-EPI@TPP-LND nanomedicine. The OCR curves were depicted in Figure 6A. It was observed that exposure to the PEG-Dendron-EPI@TPP-LND nanomedicine markedly diminished oxygen consumption in the treated cells.

Subsequent quantitative analysis of mitochondrial respiration parameters (Figure 6B) revealed that after treatment with PEG-Dendron-EPI@TPP-LND, both the basal and maximal OCR in 4T1 cells were significantly reduced. This result suggested that the nanomedicine not only suppressed energy production under a basal condition, but also markedly compromised the capacity to reach the maximal respiratory potential. The observed reduction in the maximal OCR was predominantly attributed to the suppression of the spare respiratory capacity (SRC) by the nanomedicine, a vital parameter indicative of metabolic flexibility and adaptability of tumor cells to meet energy demands for their rapid proliferation (Figure 6C). Interestingly, treatment with free TPP-LND resulted in a reduction in the basal OCR, but an increase in the SRC, thus the maximal OCR was not impacted by free TPP-LND. This may be ascribed to compensatory metabolic adaptation of tumor cells after initial treatment with free TPP-LND. Conversely, exposure to PEG-Dendron-EPI@TPP-LND effectively circumvented compensatory upregulation in the SRC, thus it could be a more potent inhibitor for mitochondrial respiration. In addition to diminishing mitochondrial respiration, PEG-Dendron-EPI@TPP-LND treatment markedly reduced ATP production in 4T1 cells (Figure 6D). The ATP production rate in cells exposed to PEG-Dendron-EPI@TPP-LND was reduced to 36.54 ± 1.50% compared to 100% in the control group (the original data shown in Figure S46), which was the lowest ATP level among all treatment groups.

Since intracellular ATP production is highly dynamic and adaptable, particularly in cancer cells, we monitored real-time changes in the ATP production rate after treatment with the nanomedicine. OCR and proton efflux rate (PER) kinetic profiles were generated from real-time ATP rate assays (Figure 6E and Figure S47). Quantitative analysis of the kinetic profile revealed that PEG-Dendron-EPI@TPP-LND significantly decreased both the mitochondrial ATP (mitoATP) and total ATP production rates (Figure 6F-6G).

Furthermore, metabolic phenotyping (Figure 6H) and the ATP rate index (Figure 6I) which is the ratio of mitoATP production to glycoATP production confirmed that incubation with PEG-Dendron-EPI@TPP-LND effectively reprogramed the OXPHOS-dependent metabolic phenotype which is characteristic in 4T1 cells. Notably, treatment with free TPP-LND led to a reduction in mitoATP production, while this treatment induced a compensatory increase in glycoATP production. However, treatment with PEG-Dendron-EPI@TPP-LND blocked this compensatory mechanism, thereby resulting in more comprehensive inhibition of energy metabolism in TNBC cells. Additionally, the temporal effect of PEG-Dendron-EPI@TPP-LND treatment on energy metabolism in 4T1 cells was examined. It was observed that the suppression of mitoATP production was time-dependent, and an extended treatment duration led to more substantial reduction in the mitoATP production rate (Figure 6J).

Collectively, these results supported that the PEG-Dendron-EPI@TPP-LND nanomedicine effectively reprogramed the metabolic phenotype of TNBC cells by suppressing mitochondrial OXPHOS and preventing compensatory upregulation of glycolysis, thus treatment with the nanomedicine to target the metabolic characteristics could be a promising strategy for highly metastatic TNBC cells.

### Metabolic targeting of OXPHOS via the nanomedicine enhanced chemosensitivity and safety in TNBC

**Figure 7:**
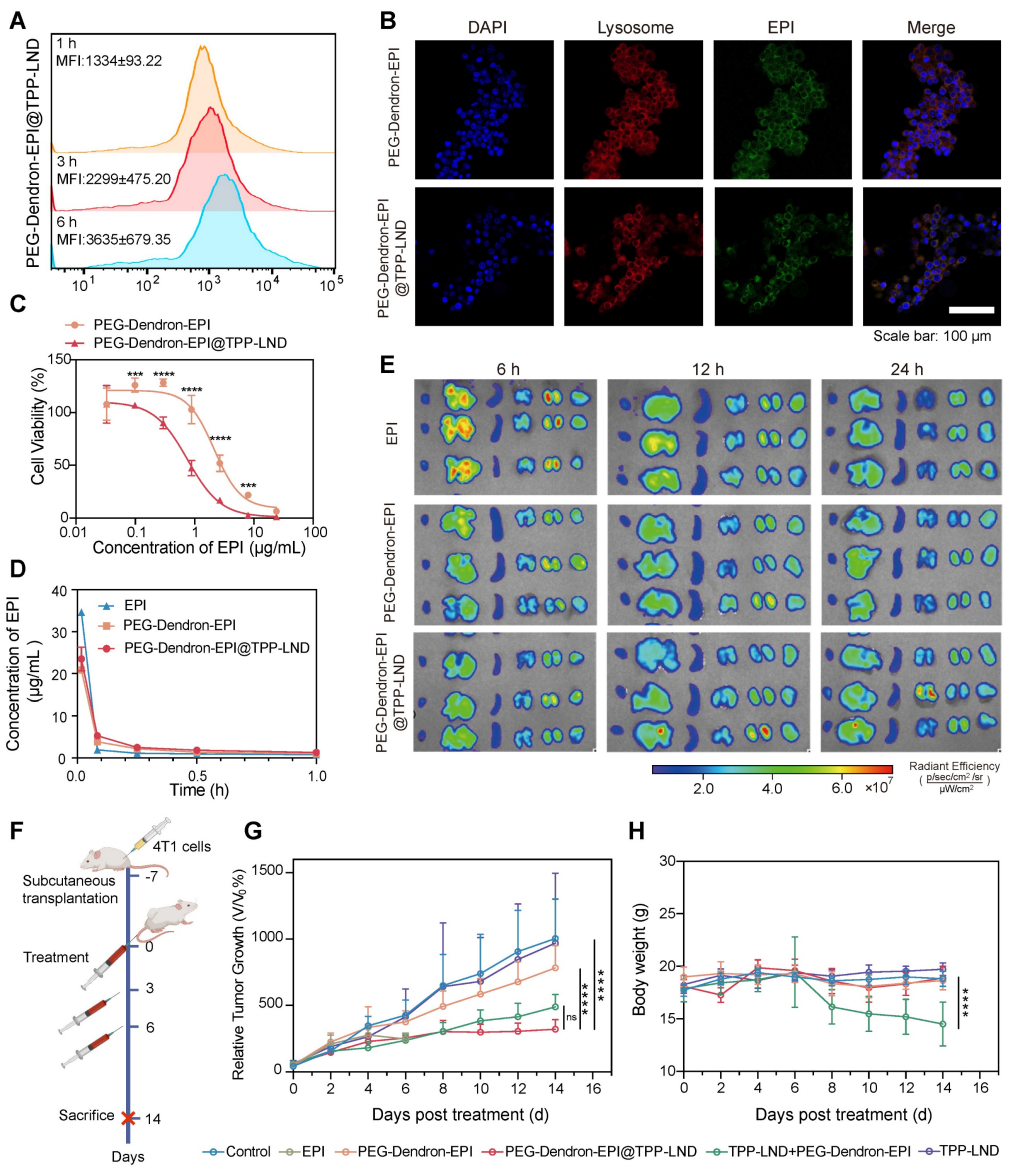
**Evaluation of *in vitro* and *in vivo* therapeutic efficacy of the PEG-Dendron-EPI@TPP-LND nanomedicine.** (A) Mean fluorescence intensity (MFI) of PEG-Dendron-EPI@TPP-LND after cellular uptake by 4T1 cells at 1, 3, and 6 h post-incubation via flow cytometry analysis. (B) Confocal fluorescence microscopy images for localization of EPI (green) and PEG-Dendron-EPI@TPP-LND within lysosomes of 4T1 cells after a 6 h treatment at an EPI equivalent concentration of 10 μg/mL. Nuclei: blue; lysosomes: red; scale bar = 100 μm. (C) Dose-dependent cytotoxic effects of EPI and PEG-Dendron-EPI@TPP-LND against 4T1 cells after a 48-h treatment. (D) Pharmacokinetic profiles of EPI, PEG-Dendron-EPI, and PEG-Dendron-EPI@TPP-LND in mice over a 1-h period post-injection, with excitation at 460 nm and emission at 620 nm. (E) *Ex vivo* fluorescence images of major organs and tumors at 6, 12, and 24 h post administration of EPI, PEG-Dendron-EPI, and PEG-Dendron-EPI@TPP-LND. (F) The experimental timeline for *in vivo* treatment of 4T1 tumor-bearing mice. (G) Changes in the tumor volume in the mice treated with control, EPI, PEG-Dendron-EPI, PEG-Dendron-EPI@TPP-LND, TPP-LND, or PEG-Dendron-EPI+TPP-LND during the treatment course. (PEG-Dendron-EPI@TPP-LND vs. TPP-LND + PEG-Dendron-EPI: *P* = 0.8850, PEG-Dendron-EPI@TPP-LND vs. PEG-Dendron-EPI: *P* < 0.0001, and PEG-Dendron-EPI@TPP-LND vs. Control: *P* < 0.0001) (H) Changes in the body weight during the treatment period. The mice received EPI-containing formulations at an equivalent EPI dose of 8 mg/kg per administration. (TPP-LND + PEG-Dendron-EPI vs. Control/ PEG-Dendron-EPI/ PEG-Dendron-EPI@TPP-LND/ TPP-LND: *P* < 0.0001).

Flow cytometry analysis indicated that cellular uptake of PEG-Dendron-EPI@TPP-LND by 4T1 cells was a time-dependent process. The mean fluorescence intensity (MFI) of PEG-Dendron-EPI@TPP-LND in 4T1 cells increased progressively from 1 h to 6 h post-treatment (Figure 7A). Confocal microscopy observations confirmed co-localization of fluorescence signal from EPI (green) with that from the lysosomal marker (red) at 6 h post treatment, indicating no significant difference in the intracellular distribution between PEG-Dendron-EPI and PEG-Dendron-EPI@TPP-LND (Figure 7B, Figure S48). The location of PEG-Dendron-EPI@TPP-LND in the lysosome suggested that the acid-responsive hydrazone bond could be cleaved at a low pH, allowing for the successful intracellular release of EPI and TPP-LND (Figure S49-S50). Additionally, PEG-Dendron-EPI@TPP-LND is relatively stable in three different media (Figure S51). Cytotoxicity assay results showed that PEG-Dendron-EPI@TPP-LND displayed greater cytotoxicity than PEG-Dendron-EPI. A lower IC_50_ of PEG-Dendron-EPI@TPP-LND against 4T1 cells after 48 h indicated its enhanced cytotoxic effect against TNBC cells through simultaneous action of EPI and TPP-LND (Figure 7C). A similar plasma concentration profile was seen for both PEG-Dendron-EPI and PEG-Dendron-EPI@TPP-LND within the first hour post-injection via pharmacokinetic analysis, suggesting encapsulation of TPP-LND in the EPI prodrug did not have an impact on systemic circulation of the EPI prodrug (Figure 7D, Figure S52). *Ex vivo* imaging at 6, 12, and 24 h post-administration revealed comparable biodistribution of PEG-Dendron-EPI and PEG-Dendron-EPI@TPP-LND in major organs, confirming that the encapsulation process did not affect the tissue distribution of the EPI prodrug (Figure 7E).

To evaluate the antitumor efficacy and biosafety of PEG-Dendron-EPI@TPP-LND *in vivo*, 4T1 tumor-bearing mice were treated every 48 h according to the protocol outlined in Figure 7F (Figure S53). Treatment with either PEG-Dendron-EPI@TPP-LND or the combination of PEG-Dendron-EPI with free TPP-LND significantly inhibited tumor growth compared to the control group and the group treated with PEG-Dendron-EPI alone according to the tumor volume curves shown in Figure 7G. However, treatment with free TPP-LND alone resulted in an increase in the tumor volume, which may be attributed to inadequate tumor accumulation, off-target effects, and compensatory changes in tumor cell metabolism at a low concentration of TPP-LND. EPI treatment was accompanied with severe toxicity. A high mortality rate was seen and five out of seven mice died after the second dose. Mice administered with a physical mixture of PEG-Dendron-EPI and TPP-LND experienced a significant reduction in the body weight down to 81.1% of the baseline (*P* < 0.05), as well as abnormalities in liver and kidney tissue (Figure S54). These results confirm the systemic toxicity of the physical mixture formulation. Conversely, the mice treated with PEG-Dendron-EPI@TPP-LND maintained a stable body weight throughout the study duration (Figure 7H) and displayed normal histopathological characteristics (Figure S54). In addition, PEG-Dendron-EPI@TPP-LND was associated with normal blood biochemical parameters (Tables S7-S8) and normal histopathological findings (Figure S55) in healthy mice, thereby affirming the superior safety profile of this nanocarrier-based delivery system (Figure S55). Collectively, these results suggested that PEG-Dendron-EPI@TPP-LND synergistically enhanced the efficacy of the chemotherapeutic agent (EPI) and mitigated its systemic toxicity, therefore, it could be a safe and effective OXPHOS-targeting nanomedicine.

## Discussion

Chemotherapy remains the mainstay of treatment options for triple-negative breast cancer (TNBC) due to lack of distinct molecular targets and very few therapeutic modalities. It has been demonstrated that metabolic adaptation of tumor cells may significantly compromise therapeutic responses, and the strategy of targeting metabolic adaptation may be used to enhance chemosensitivity and improve chemotherapeutic efficacy 39-42. In this study, the energy metabolism of TNBC was initially profiled, which provided a cornerstone to develop effective therapeutic strategies and improve patient outcomes. It has been widely accepted that oxidative phosphorylation (OXPHOS), in contrast to aerobic glycolysis (the Warburg effect) that provides molecular intermediates and energy for rapid proliferation under a normal condition, plays a critical role in maintaining viability and proliferation of tumor cells under chemotherapeutic stress 43-45. Our data analysis suggested that an elevated level of OXPHOS of tumors was correlated with poor patient prognosis including shortened survival rates and decreased sensitivity to commonly administered chemotherapeutic agents, such as topoisomerase inhibitors like EPI. In addition, the *in vitro* energy metabolism analysis revealed that the 4T1 TNBC cell line, which serves as a model for the stage IV disease, predominantly relied on OXPHOS for energy production rather than glycolysis. Consequently, this OXPHOS-preferable 4T1 TNBC cell line was selected for following investigations. An OXPHOS intervention inhibitor, TPP-LND, was synthesized by modification of LND. The triphenylphosphonium (TPP^+^) moiety promoted mitochondrial targeting in tumor cells by harnessing an elevated membrane potential gradient between the inner and outer mitochondrial membranes 46, thereby enhancing the inhibitory effect of LND on the mitochondrial respiratory chain. Our results confirmed that TPP-LND specifically targeted the mitochondria, which was in accordance with other TPP-modified OXPHOS inhibitors, such as mito-metformin 47, 48 and mito-atovaquone 49. In addition, HY001, a TPP derivative, targets mitochondria to eliminate cancer stem cells 50, 51. TPP-LND exhibited distinct efficacy in inhibiting ATP production via OXPHOS, and it may trigger the mitochondria-mediated apoptosis pathway to induce cancer cell death 52. It has been demonstrated that OXPHOS inhibition can sensitize cancer cells to chemotherapy by forcing them into a less vigorous metabolic state 53, 54. In this context, OXPHOS inhibition may enhance sensitivity of TNBC cells to chemotherapeutic agents such as EPI, thus TPP-LND could be combined with EPI as a novel therapeutic strategy to improve therapeutic outcomes in this challenging cancer.

However, the use of this inhibitor faces challenges such as druggability, which hampers its combination with chemotherapeutic agents *in vivo*. In addition, metabolic processes of tumor cells are highly complex. Inhibition of a single pathway within cellular energy metabolism can trigger the activation of alternative compensatory mechanisms to restore energy supply for tumor cells. These mechanisms have been identified, including activation of alternative metabolic pathways, adaptation of the mitochondrial function, reprogramming of energy metabolism, and mobilization of energy reserves 55, 56. Cell viability results indicated that TPP-LND treatment alone may induce a compensatory increase in ATP production via glycolysis at a low concentration without inducing cytotoxic effects. When TPP-LND inhibited OXPHOS in tumor cells, energy production through an alternative metabolic pathway, such as glycolysis, was activated to maintain cellular energy homeostasis. Although TPP-LND at a low dose could effectively inhibit OXPHOS, treatment with TPP-LND did not successfully prevent tumor cell progression and induce damage or apoptosis of tumor cells. Instead, a metabolic shift to glycolysis was stimulated to support tumor cell proliferation akin to the Warburg effect, which underscores the challenges associated with employing metabolic intervention strategies for the treatment of TNBC.

To address the druggability challenge of TPP-LND, three amphiphilic PEG-EPI prodrugs for co-delivery of EPI and TPP-LND were developed. Three prodrugs could self-assemble into nanoscale particles. They exhibited no significant discrepancies in *in vivo* pharmacokinetics and biodistribution, however, the PEG-Dendron-EPI prodrug exhibited superior performance in terms of cellular uptake, cytotoxicity, and notably, a high efficiency and a high drug loading for encapsulating TPP-LND. The enhanced encapsulation performance of PEG-Dendron-EPI may be attributable to its dendritic structure, which may facilitate intermolecular interaction between EPI and TPP-LND, thereby promoting the encapsulation of TPP-LND within a hydrophobic cavity of PEG-Dendron-EPI during the self-assembly process 57-61. Furthermore, in the combined application of EPI and TPP-LND, it was observed that TPP-LND at a low concentration (5 µM or 10 µM) did not show significant cytotoxicity, but enhanced the efficacy of EPI released from PEG-Dendron-EPI. Subsequently, a sequential dosing regimen was explored to induce a metabolic shift by TPP-LND and then exert cytotoxic action by PEG-Dendron-EPI. However, pretreatment with TPP-LND for 24 h followed by PEG-Dendron-EPI treatment for 48 h was less effective than simultaneous treatment with both TPP-LND and PEG-Dendron-EPI for 48 h. Therefore, concurrent action of TPP-LND and PEG-Dendron-EPI at the target site was essential to achieve a significant antitumor effect.

Co-delivery of TPP-LND and PEG-Dendron-EPI was developed by encapsulating TPP-LND within PEG-Dendron-EPI at an optimal ratio to form a nanomedicine, PEG-Dendron-EPI@TPP-LND. In this PEG-Dendron-EPI@TPP-LND nanomedicine, hydrazone bonds were employed to conjugate EPI, and upon reaching an intracellular location with a low pH, hydrazone bonds were cleaved to promote acid-responsive release of EPI. The release of hydrophobic EPI disrupted the hydrophilic-lipophilic balance of the nanomedicine, leading to the release of TPP-LND from the hydrophobic cavity. It was found that after uptake of PEG-Dendron-EPI@TPP-LND by tumor cells, PEG-Dendron-EPI@TPP-LND was transported to lysosomes. Within lysosomes, EPI and TPP-LND were effectively released from PEG-Dendron-EPI@TPP-LND to exert their simultaneous action on tumor cells.

The synergistic action of PEG-Dendron-EPI@TPP-LND was demonstrated on metabolic interventions in TNBC cells. It was observed that PEG-Dendron-EPI@TPP-LND effectively reduced the mitoATP production rate, and blocked the activation of a compensatory energy pathway through glycolysis for ATP production, thus the treated cells could not reach their maximum respiratory capacity under a basal condition. Consequently, there was a marked reduction in the total ATP production rate, indicating effective inhibition of cellular energy metabolism by PEG-Dendron-EPI@TPP-LND. The synergistic effect of PEG-Dendron-EPI@TPP-LND was subsequently demonstrated to kill TNBC cells. The nanomedicine displayed significantly higher cytotoxicity than PEG-Dendron-EPI or TPP-LND alone, indicating PEG-Dendron-EPI@TPP-LND not only enhanced the sensitivity of TNBC cells to EPI through metabolic disruption induced by TPP-LND, but also effectively reduced the cell viability by preventing them acquiring energy through alternative pathways. Finally, treatment of tumor-bearing mice with PEG-Dendron-EPI@TPP-LND resulted in a remarkable reduction in the tumor volume, consolidating the synergistic effect of this nanomedicine.

In addition, PEG-Dendron-EPI@TPP-LND pronouncedly mitigated the toxicity concerns associated with intravenous administration of TPP-LND and EPI. In the cohort treated with a physical mixture of PEG-Dendron-EPI and TPP-LND, there was a distinct reduction in the body weight of the mice. In contrast to the physical mixture, treatment with PEG-Dendron-EPI@TPP-LND did not induce any significant changes in the body weight, animal behavior or other parameters in tumor-bearing or healthy mice, supporting its excellent biosafety. Overall, PEG-Dendron-EPI@TPP-LND as a nanomedicine addressed a significant challenge in the *in vivo* application of TPP-LND as a mitochondrial inhibitor through an EPI prodrug delivery system, and this novel nanomedicine combining both TPP-LND and EPI was demonstrated to effectively attack metabolic vulnerabilities of TNBC and curb tumor growth in the animal model with excellent biosafety.

## Conclusion

A correlation between OXPHOS and TNBC chemosensitivity was initially established, providing the foundation in this study that metabolic targeting via mitochondrial inhibition could enhance the sensitivity of tumors to conventional chemotherapy. TPP-LND as an OXPHOS inhibitor was encapsulated into an EPI prodrug as a delivery nanocarrier prepared by conjugating EPI with PEG-Dendron via an acid-responsive hydrazone bond. The resulting nanomedicine, PEG-Dendron-EPI@TPP-LND, enhanced drug loading of EPI and TPP-LND and their pharmacokinetics. The nanomedicine simultaneously suppressed OXPHOS to amplify the cytotoxic effect of EPI and prevented the activation of the compensatory glycolysis pathway. This nanomedicine successfully circumvented metabolic adaptation of aggressive cancer cells and inhibited tumor cell growth with excellent biosafety. This strategy proposed in this study could be exploited for OXPHOS modulation, ultimately improving therapeutic outcomes for patients with TNBC.

## Materials and Methods

### Bioinformatics analysis

Bulk RNA sequencing data for breast cancer patients as well as their clinical information were obtained from The Cancer Genome Atlas (TCGA-BRCA). The OXPHOS gene set (KEGG_OXIDATIVE_PHOSPHORYLATION.v2024.1.Hs.gmt) sourced from the Molecular Signatures Database (MSigDB) was employed for gene set enrichment analysis. Single-sample gene set enrichment analysis (ssGSEA) was performed via “GSVA”, an R package, to calculate OXPHOS scores for differentiating the level of upregulation or downregulation of the OXPHOS gene set for each sample. These samples were then categorized into high and low OXPHOS groups for subsequent clinical analysis.

The Kaplan-Meier method in “Survival” and “Survminer,” two R packages, was utilized to generate progression-free survival (PFS), disease-free survival (DFS), and disease-specific survival (DSS) curves for breast cancer patients. The survival differences between groups with high and low OXPHOS scores were compared using the Log-rank test to build a correlation between the OXPHOS level and patient prognosis.

The impact of OXPHOS levels on the sensitivity to chemotherapy in breast cancer patients was evaluated via an R package “OncoPredict” and its training data (source: https://osf.io/c6tfx/). The IC_50_ values of therapeutic drugs for breast cancer patients were correlated with the oxidative phosphorylation levels of these patients.

### Cell culture and animal models

4T1 cells sourced from the Cell Bank of the Chinese Academy of Sciences were routinely cultured according to the recommended cell culture conditions. All animal experiments were approved by the Animal Ethics Committee of West China Hospital, Sichuan University (Approval No. 20230621005). Female BALB/c mice (6-8 weeks) supplied by Chengdu Ensiweier Biotechnology Co. were subcutaneously injected with 50 μL of 1.0 × 10^6^ 4T1 cells in PBS on the right dorsum to establish a subcutaneous 4T1 model.

### Synthesis of PEG-EPI, PEG-Glu-EPI, and PEG-Dendron-EPI

To introduce varying numbers of EPI molecules at the termini of PEG chains, small molecule compounds modified with DBCO containing different numbers of hydrazide bonds were first synthesized. Subsequently, these DBCO-functionalized small molecules were conjugated to azide-terminated PEG chains via a “click chemistry” reaction. Finally, the ketone carbonyl groups of EPI reacted with the hydrazine groups provided by the hydrazide functionalities on the polymer, forming hydrazone linkages, thereby yielding structurally diverse PEG-EPI prodrugs. The details can be seen in the Supplementary Information (SI). We sequentially synthesized DBCO-NHNHFmoc, DBCO-Glu-(NHNHFmoc)_2_, DBCO-G2(Glu)-(NHNHFmoc)_4_, PEG_5k_-N_3_, PEG_5k_-(NHNHFmoc)_1/2/4_, PEG_5k_-(NHNH_2_)_1/2/4_, and three polymer prodrugs PEG-EPI, PEG-Glu-EPI and PEG-Dendron-EPI. Specific synthetic procedures for these chemical compounds and their characterizations by ^1^H NMR and HRMS can be found in SI.

### Synthesis of TPP-LND

A mixture of 4-carboxybutyltriphenylphosphonium bromide (4.88 g, 11 mmol), N-Boc-ethylenediamine (2.34 g, 15 mmol), HOBt (2.23 g, 17 mmol), and HBTU (6.26 g, 17 mmol) in anhydrous DMF (50 mL) was treated with DIPEA (9.1 mL, 55 mmol) under N_2_ at 0 °C. After stirring at room temperature for 12 h, the reaction was quenched with ethyl acetate (300 mL), washed sequentially with saturated NaHCO_3_, 0.1 M HCl, and saturated sodium chloride. The organic phase was concentrated to 20 mL and purified by silica gel chromatography (DCM:MeOH = 10:1) to yield TPP-NHBoc as a white solid (4.95 g, 77%).

TPP-NHBoc (304 mg, 0.52 mmol) in DCM (5 mL) was treated with TFA (5 mL) at 0 °C. After 3 h at room temperature, the solvent was evaporated, and the residue was washed with Et_2_O and dried under vacuum to give TPP-NH_2_·TFA. A solution of TPP-NH_2_·TFA (0.52 mmol), lonidamine (200 mg, 0.623 mmol), HOBt (105 mg, 0.779 mmol), and HBTU (295 mg, 0.779 mmol) in DMF was treated with DIPEA (0.428 mL, 2.60 mmol) under N_2_ at 0 °C. After stirring at room temperature for 12 h, the mixture was processed in a similar manner as the above and purified by silica gel chromatography (DCM:MeOH = 40:1) to yield TPP-LND as a white solid (302 mg, 74%).

### Characterization of prodrug-based nanoparticles

The aqueous particle size distribution, zeta potential, and morphology of prodrug-based nanoparticles were characterized by dynamic light scattering (DLS) and transmission electron microscopy (TEM). Briefly, polymeric prodrugs were dissolved in deionized water and analyzed *via* a NanoBrook Series Size and Zeta Potential Analyzer (Brookhaven Instruments, NanoBrook Omni, New York, USA). Each sample was tested six times to ensure reproducibility. For TEM imaging, 10 μL of a freshly prepared prodrug solution (0.5 mg/mL) was deposited onto carbon-coated copper grids. Excess solution was removed by filter paper, and grids were air-dried to allow complete solvent evaporation prior to observation.

The stability of PEG-EPI, PEG-Glu-EPI, and PEG-Dendron-EPI in water and the stability of PEG-Dendron-EPI@TPP-LND in different media (RPMI 1640 medium, PBS solution containing 10% FBS, and aqueous solution) were monitored by measuring particle size at time points of 0 h, 6 h, 12 h, 24 h, and 48 h.

### Cellular cytotoxicity assays

4T1 cells in 96-well plates were treated with EPI, PEG-EPI, PEG-Glu-EPI or PEG-Dendron-EPI at a gradient concentration from 0.01 μg/mL to 24 μg/mL for 48 h. The spent medium was replaced with the fresh medium containing the CCK-8 kit and cells were incubated for 1.5 h at 37 °C. A multimode microplate reader was used to acquire the OD_450_ value of each well, and the relative cell viability was calculated using GraphPad Prism.

### *In vitro* drug release

PEG-EPI, PEG-Glu-EPI or PEG-Dendron-EPI was dissolved in ultrapure water (EPI concentration: 0.3 mg/mL, volume: 1 mL) and the solution was transferred into a dialysis membrane (MWCO = 2000 Da). The membrane was submerged in PBS (40 mL) at different pH values (5.4, 6.7, 7.4) in a shaking bed (120 rpm) at 37 ℃ for incubation. At 1 h, 2 h, 4 h, 6 h, 8 h, 12 h, 24 h, 36 h, 48 h, or 72 h post incubation, 1.5 mL of the release medium was collected, and the same amount of the corresponding release medium at the same pH value was replenished to maintain a constant volume of the release medium. The samples were stored temporarily at 4 ℃ in the dark before analysis via a multifunctional microplate reader. The drug release from PEG-Dendron-EPI@TPP-LND was evaluated using the same method described above, with the difference that the released TPP-LND was detected by HPLC (0.1% TFA aqueous solution/acetonitrile as mobile phase; a detection wavelength at 298 nm; and a retention time at 23 min).

### Cellular uptake and subcellular location

Assessment of cellular uptake of EPI-derived compounds was performed by flow cytometry. After seeding 1×10^4^ 4T1 cells per well into a 12-well plate, free EPI, PEG-EPI, PEG-Glu-EPI, and PEG-Dendron-EPI at an equivalent EPI concentration of 5 μg/mL were added into the plate and incubated with cells for 1 h, 3 h, and 6 h. The cells were collected after each incubation time and centrifuged at 300 g at 4 °C. After PBS washing 2-3 times, cells were resuspended with 300 μL PBS, and the suspension was transferred to a flow tube for flow cytometry analysis (BD). The data were analyzed using FlowJo. Three replicates for each experimental sample were used.

After EPI, PEG-EPI, PEG-Glu-EPI and PEG-Dendron-EPI at an EPI concentration of 5 μg/mL were incubated with 4T1 cells for 6 h, a lysosomal staining dye (Lyso Tracker Deep Red) was incubated with cells for 1 h. The EPI drug distribution in the organelles was monitored via confocal laser scanning microscopy (CLSM). Three replicates for each experimental sample were used.

### *In vivo* biodistribution

4T1 tumor-bearing mice were intravenously injected with 200 μL of Cy5 solution or Cy5-labeled nanoparticles based on PEG-EPI, PEG-Glu-EPI and PEG-Dendron-EPI, at a Cy5 concentration of 150 μg/mL. Cy5-labeled nanoparticles for *in vivo* imaging were prepared according to a previously reported method 62 and the Cy5 loading content was determined by UV-Vis spectroscopy. The mice were anesthetized with isoflurane at 1 h, 3 h, 6 h, 12 h, 24 h, 36 h, 48 h and 72 h post injection for *in vivo* imaging (IVIS Spectrum). Major organs including the heart, lung, liver, spleen and kidney and tumors were collected at 72 h post injection for *ex vivo* imaging.

### Pharmacokinetics

Six-eight-weeks BALB/c female mice at a weight of approximate 20 g were selected for this study. Two hundreds (200) μL of (1) EPI, (2) PEG-EPI, (3) PEG-Glu-EPI or (4) PEG-Dendron-EPI was injected into the mice through the tail vein at an EPI dose of 8 mg/kg. The time points for blood collection were established at 1 min, 5 min, 30 min, 1 h, 2 h, 4 h, 8 h, 12 h, 24 h, 36 h, 48 h, and 72 h post injection. At each time point, 20 μL of blood was withdrawn from the orbital venous plexus of mice and added into a sodium heparin EP tube containing 80 μL of ddH_2_O. DMSO was added into each tube after the blood sample was collected, and subsequently the fluorescence intensity (Ex: 485 nm, Em: 595 nm) was measured. The data obtained from the experiment was processed by GraphPad Prism.

### *Ex vivo* imaging

After establishing a 4T1 subcutaneous tumor model in mice, EPI, PEG-EPI, PEG-Glu-EPI, and PEG-Dendron-EPI were injected intravenously at a dose of 8 mg EPI/kg. At 6 h, 12 h, and 24 h after injection, the mice were sacrificed, and the tumor was collected. *Ex vivo* imaging of the tumor sample was performed using IVIS to measure the fluorescence intensity in the tumor tissue. The quantitative data was processed and analyzed using GraphPad Prism.

### Assessment of cytotoxicity of combination therapy

The interaction between PEG-Dendron-EPI and TPP-LND was probed through cytotoxicity experiments. 4T1 cells were incubated with LND and TPP-LND for 24 h. The concentration of TPP-LND was fixed at 5 μM or 10 μM, and a concentration gradient of EPI in PEG-Dendron-EPI was applied. Subsequently, based on the results obtained after 24 h, 4T1 cells were further incubated with TPP-LND for 48 h. After 48 h incubation, the cell viability was detected using a CCK-8 kit. The IC_50_ value was calculated after fitting the curve of the cell viability vs the concentration gradient.

To assess the cytotoxicity against 4T1 cells by sequentially adding TPP-LND and PEG-Dendron-EPI, 4T1 cells in the logarithmic growth phase were pre-incubated with 5 μM or 10 μM TPP-LND for 24 h. PEG-Dendron-EPI at a concentration gradient was then added and incubated for an additional 24 h or 48 h. After drug incubation, the cell viability was detected using the CCK-8 kit.

### Evaluation of the loading capacity of EPI prodrugs for TPP-LND

The drug-loading capacity of three EPI prodrugs for TPP-LND was evaluated using the thin-film hydration method. Initially, PEG-EPI, PEG-Glu-EPI, and PEG-Dendron-EPI were dissolved in 10 mL methanol, respectively (EPI concentration: 0.08 mg/mL). Subsequently, TPP-LND was added to the EPI-containing methanol solution at EPI/TPP-LND mass ratios of 1:0.25, 1:0.5, 1:1, 1:1.5, or 1:2. The mixture was transferred into a 50 mL round-bottom flask and stirred in the dark for 1 h to ensure homogeneous mixing. A uniform thin film was formed on the inner wall of the flask via rotary evaporation under reduced pressure. Residual organic solvents were completely removed by vacuum drying overnight. The film was then hydrated with deionized water under gentle stirring at room temperature, followed by sonication for 5 min to disperse aggregates. The resulting suspension was filtered through a 0.45 μm membrane to remove unencapsulated TPP-LND. The filtrate was lyophilized to obtain TPP-LND-loaded nanoparticles. TPP-LND was analyzed via HPLC (0.1% TFA and acetonitrile as the mobile phase; a detection wavelength at 298 nm; and a retention time at 23 min). The amount of TPP-LND in the final product, the final drug loading and encapsulation efficiency, were calculated based on the standard curves of TPP-LND.

### Oxygen consumption rate and real-time ATP measurements

The energy metabolism profile and metabolic phenotypes of tumor cells were assessed using the Seahorse XF24 analyzer (Agilent Technologies) by following the Seahorse assay protocol. Briefly, 4T1 cells were seeded in a culture plate and treated with either 10 μM of LND, PEG-EPI containing 1.95 μg/mL EPI, or PEG-Dendron-EPI@TPP-LND containing an equivalent concentration of EPI on the subsequent day. The cells were incubated for an additional 24 h.

To measure the oxygen consumption rate (OCR), the cells were washed with PBS three times on the day of measurement and then incubated with a fresh Seahorse MEM XF medium (Agilent, Cat. No. 103575-100). The medium was free of phenol red and bicarbonate, and it was supplemented with 2 mM L-glutamine, 10 mM glucose, and 1 mM sodium pyruvate. The medium was equilibrated in a 37 °C, non-CO_2_ incubator for 30 min. After baseline measurements, the mitochondrial function was assessed by sequentially adding 5 μM oligomycin A, 0.75 μM FCCP, and 1 μM of rotenone/antimycin A. Key mitochondrial parameters were calculated, including the basal respiration rate, the maximal respiration rate, the ATP production rate, and the spare respiratory capacity.

The experimental conditions and reagents in the OCR protocol were used for real-time ATP production rate measurements. During the assay phase, 5 μM oligomycin A and 1 μM of rotenone and antimycin A were supplemented. The extracellular acidification rate (ECAR), the OCR, and the proton efflux rate (PER) were monitored via the Seahorse XF24 analyzer. The glycolytic ATP production rate and the mitochondrial ATP production rate were calculated using the Wave Desktop and Controller 2.6 software (version 2.6.1).

### Biological effects of PEG-Dendron-EPI@TPP-LND

After synthesizing PEG-Dendron-EPI@TPP-LND, its biological properties were assessed, including cytotoxicity, pharmacokinetics, organelle co-localization, and *ex vivo* imaging. Cellular uptake of PEG-Dendron-EPI@TPP-LND at different time points was examined via flow cytometry. Cytotoxic effects of PEG-Dendron-EPI@TPP-LND and PEG-Dendron-EPI on 4T1 cells were evaluated via a CCK-8 kit. The blood half-life of PEG-Dendron-EPI@TPP-LND was examined using the same method described in the **Pharmacokinetics subsection**. The experimental method in “Cellular uptake and subcellular location” was applied to identify intracellular localization of PEG-Dendron-EPI@TPP-LND after cellular uptake at a drug concentration of 5 μg/mL for an incubation time of 8 h. *Ex vivo* imaging experiments were performed at 6 h, 12 h, and 24 h.

### *In vivo* anti-tumor study

Before conducting *in vivo* drug therapy, the tumor-bearing mice model was established. PEG-Dendron-EPI was administered via tail vein injection at a dose of 8 mg/kg per mouse to determine the dosing interval. Mice were sacrificed at 1 h, 3 h, 6 h, 12 h, and 24 h after administration, and *ex vivo* imaging of tissues and organs was performed using the IVIS system. The dosing interval for *in vivo* therapy was determined based on the biodistribution result.

4T1 tumor-bearing mice received (1) saline, (2) EPI, (3) PEG-Dendron-EPI, (4) PEG-Dendron-EPI@TPP-LND, (5) a physical mixture of PEG-Dendron-EPI and TPP-LND, and (6) TPP-LND at an EPI dose of 8.0 mg/kg (The dose of TPP-LND in groups 5 and 6 was equivalent to the corresponding dose of TPP-LND in group 4 when 8 mg/kg of drug EPI was administered). The treatment was repeated every three days three times. The tumor length and width were recorded every two days and the weight of the mice was monitored. The volume of the tumor was calculated by the following formula: V(mm^3^) = π/6 × Length × Width^2^. The experiment was terminated when a loss in the mice body weight reached more than 20%. Upon completion of the experiment, the mice were executed, and their major organs were removed and fixed. Paraffin sections were made for hematoxylin and eosin (H&E) staining to evaluate *in vivo* biosafety.

To evaluate the safety of the drugs, normal mice received intravenous injections of different drug groups via the tail vein on Days 0, 3, and 6, as shown in Figure 7F. On the seventh day, the mice were euthanized to facilitate tissue and blood analysis. Their major organs were then excised, fixed in paraformaldehyde, embedded in paraffin, sectioned, and stained with H&E. Subsequently, the sections were scanned using the VS200 whole-slide bright-field imaging system (Olympus). Blood samples were collected and subjected to centrifugation at 3500 rpm for 10 min. The resulting serum was analyzed for biochemical indicators of liver and kidney function, including alanine aminotransferase (ALT), aspartate aminotransferase (AST), albumin (ALB), uric acid (UA), and urea (UR).

### Statistical analysis

GraphPad Prism was used for data analysis of at least three independent measurements, and quantitative results were expressed as mean ± standard deviation (± S.D.). For comparison between two groups, a two-tailed *t*-test was applied. For drug response prediction analyses, comparison of the IC₅₀ values between different groups was conducted using the Wilcoxon rank-sum test. For comparison among more than two groups, one-way analysis of variance (ANOVA) was performed, followed by Dunnett's test to evaluate pairwise group differences. When multiple conditions were present, two-way ANOVA was conducted, and the Šidák's method was applied for post hoc multiple comparison. A *P*-value < 0.05 was considered to be statistically significant for all analyses.

## Supplementary Material

Supplementary materials and methods, figures and tables.

## Abbreviations

ALB: albumin
ALT: alanine aminotransferase
AST: aspartate aminotransferase
BRCA: Breast invasive carcinoma
CLSM: confocal laser scanning microscopy
CMC: critical micelle concentration
DFS: disease-free survival rate
DLS: dynamic light scattering
DPD: dissipative particle dynamics
DSS: disease-specific survival rates
ECAR: extracellular acidification rate
EPI: epirubicin
IC_50_: half-maximal inhibitory concentration
LND: lonidamine
OCR: oxygen consumption rate
OXPHOS: oxidative phosphorylation
PER: the proton efflux rate
PFS: progression-free survival rates
SRC: spare respiratory capacity
TCGA: The Cancer Genome Atlas Program
TEM: transmission electron microscopy
TNBC: Triple-negative breast cancer
TPP: triphenylphosphine group
TPP-LND: lonidamine conjugating with triphenylphosphine group
UA: uric acid
UR: urea
