# Supplementary material for: Metabolic Targeting of Oxidative Phosphorylation Enhances Chemosensitivity in Triple-Negative Breast Cancer via a Synergistic Nanomedicine
Source: Theranostics. 2025 Jun 23;15(15):7607–26. doi: 10.7150/thno.116250 (PMC12316031; doi:10.7150/thno.116250)
Supplement: Supplementary file 1 — Supplementary materials and methods, figures and tables. [file thnov15p7607s1.pdf]

Supplementary Materials

for

Metabolic Targeting of Oxidative Phosphorylation Enhances Chemosensitivity in Triple-Negative Breast Cancer via a Synergistic Nanomedicine

Xiaoding Shen[1,#], Hao Cai[1,#], Yihan Wang[1,2], Maodi Xie[1], Yunkun Li[1,3], Dayi Pan[1,3,*], Jing Jing[1], Qiyong Gong[1,3,4], Kui Luo[1,3,*]

1. Department of Radiology, Huaxi MR Research Center (HMRRC), Institution of Radiology and Medical Imaging, Rehabilitation Therapy, Breast Center, Institute of Breast Health Medicine, Department of Thoracic Surgery and Institute of Thoracic Oncology, Laboratory of Mitochondrial Metabolism and Perioperative Medicine, Frontiers Science Center for Disease-Related Molecular Network, State Key Laboratory of Biotherapy, West China Hospital, Sichuan University, Chengdu 610041, China.
2. West China School of Medicine, Chengdu 610041, China.
3. Functional and molecular imaging Key Laboratory of Sichuan Province, Key Laboratory of Transplant Engineering and Immunology, NHC, and Research Unit of Psychoradiology, Chinese Academy of Medical Sciences, Chengdu 610041, China.
4. Xiamen Key Lab of Psychoradiology and Neuromodulation, Department of Radiology, West China Xiamen Hospital of Sichuan University, Xiamen 361021, China.

[#]These authors contributed equally: Xiaoding Shen and Hao Cai.
*Corresponding author: Prof. Dayi Pan: dypan@scu.edu.cn; Prof. Kui Luo, E-mail: luokui@scu.edu.cn.

1. Materials and methods

1.1. Materials and characterization methods

Poly(ethylene glycol) methyl ether ($mPEG_{5k}$-OH, Mn ≈ 5,000 Da) was supplied by Sigma-Aldrich (St. Louis, MO, USA). Epirubicin hydrochloride (EPI·HCl), Cy5 and azidobenzoic acid were obtained from Macklin Biochemical Co., Ltd. (Shanghai, China). 11,12-Didehydrodibenzo[b,f]azocin-5(6H)-yl-4-oxobutanoic acid (DBCO-acid), N-Boc-ethylenediamine, Lonidamine, and 4-Carboxybutyltriphenylphosphonium bromide were purchased from Bide Pharmatech Co., Ltd. (Shanghai, China). Fmoc-hydrazine (FmocNH-NH_2) was acquired from Energy Chemical Co., Ltd. (Shanghai, China). Boc-L-glutamic acid (Boc-Glu-OH) was provided by GL Biochem Co., Ltd. (Shanghai, China).

Characterizations of monomers and polymers were conducted by ^{1}H NMR (400 MHz Bruker Avance II NMR spectrometer, AVANCE NEO, Germany) for their structure confirmation. High-performance liquid chromatography (HPLC) analyses were performed on a Shimadzu Prominence HPLC system (column: Shim-pack GIST C18, pore size: 5 μm, dimension: 4.6 × 250 mm, flow rate: 1 mL/min, column temperature: 35 °C, injection volume: 20 μL, Shimadzu Corporation, Kyoto, Japan). The EPI content was measured using an ultraviolet-visible spectrophotometer (UV-1800, Shimadzu Corporation, Tokyo, Japan).

1.2. Synthesis of Functionalized Small Molecules and Polymeric Prodrugs

A mitochondria-targeting small molecule TPP-LND and three pH-sensitive PEG-EPI polymeric prodrugs with distinct architectures were designed and synthesized. The synthetic route and structure of TPP-LND were illustrated in Scheme S1. The structural characterization of intermediates and final products was performed using ^{1}H NMR and HRMS (Figures S1-S4).

In order to prepare pH-sensitive PEG-EPI precursors, we first designed and synthesized DBCO-modified small molecules containing different numbers of hydrazide bonds, which could be efficiently coupled to the end of the azide-modified PEG chain via “click chemistry” reaction. The specific synthetic route was shown in Scheme S2. The structure of the resulting small molecule was characterized by ^{1}H NMR

and HRMS, and the results were shown in Figure S7-S16. Next, azide modification of poly(diethanolmethyl ether) (molecular weight: ~5000) allowed coupling of the DBCO-functionalized small molecule compound to PEG chain ends. After deprotection treatment to expose the amino group of the hydrazide bond, it was coupled with EPI in a weakly acidic buffer solution, and poly(ethylene glycol)-EPI precursors with different structures could be prepared. The specific synthetic routes were shown in Scheme S3 and Scheme S4. We characterized the structures of the resulting polymer intermediates and precursors by ^{1}H NMR, and the results were displayed in Figures S17-S26.

1.2.1. Synthesis of DBCO-Modified Hydrazide-Bearing Small Molecules

a. Synthesis of DBCO-NHNHFmoc

DBCO-COOH (0.40 g, 1.3 mmol), FmocNH-NH_2 (0.67 g, 2.6 mmol), and HATU (1.00 g, 2.6 mmol) were dissolved in anhydrous DMF (15 mL) under N_2. DIPEA (1.3 mL, 7.8 mmol) was added, and the mixture was stirred at 25 °C for 4 h. The reaction was quenched with ethyl acetate (200 mL), washed with saturated $NaHCO_3$, 0.1 M HCl, and brine. After drying over Na_2SO_4, the crude product was purified by silica column chromatography (DCM:MeOH = 40:1) to yield DBCO-NHNHFmoc as a white solid (0.46 g, 66%).

b. Synthesis of DBCO-Glu-$(NHNHFmoc)_2$

Boc-Glu-OH (5.0 g, 20 mmol), Fmoc-$NHNH_2$ (20.3 g, 80 mmol), and HATU (30.4 g, 80 mmol) were dissolved in DMF (150 mL). DIEA (40.1 mL, 240 mmol) was added dropwise at 0 °C, and the reaction proceeded at 4 °C for 36 h. The product was extracted with ethyl acetate, purified by silica column chromatography (PE:EA = 1:1), and deprotected with TFA/DCM (1:1) to yield (NH_2·TFA)-Glu-$(NHNHFmoc)_2$. Subsequent conjugation with DBCO-COOH (0.39 g, 1.3 mmol) using HATU (0.72 g, 1.9 mmol) and DIEA (1.3 mL, 7.8 mmol) in DMF produced DBCO-Glu-$(NHNHFmoc)_2$ (0.87 g, 71%).

c. Synthesis of DBCO-G2(Glu)-$(NHNHFmoc)_4$

Boc-G2(Glu)-$(NHNHFmoc)_4$ was synthesized via stepwise coupling of Boc-Glu-OH (0.91 g, 3.7 mmol) with (NH_2·TFA)-Glu-$(NHNHFmoc)_2$ (6.88 g, 11.1 mmol) using

HATU (4.21 g, 11.1 mmol) and DIEA (3.1 mL, 18.5 mmol). Deprotection with TFA/DCM (1:1) followed by conjugation with DBCO-COOH (254 mg, 0.83 mmol) yielded DBCO-G2(Glu)-$(NHNHFmoc)_4$ (0.88 g, 65%).

1.2.2. Synthesis of pH-Sensitive PEG-EPI Prodrugs

a. Preparation of $mPEG_{5k}$-N_3

$mPEG_{5k}$ -OH (5.0 g) was functionalized with azidobenzoic acid (0.653 g, 4 mmol) using DCC (0.83 g, 10.5 mmol) and DMAP (0.49 g, 4 mmol) in DCM/DMF (5:1). After 36 h functionalization, the product was dialyzed (MWCO: 2 kDa) and lyophilized to yield $mPEG_{5k}$-N_3. The target products were characterized by ^{1}H NMR spectroscopy and IR spectroscopy.

b. Conjugation of DBCO-Modified Compounds to $mPEG_{5k}$-N_3

$mPEG_{5k}$-N_3 (900 mg, 164 μmol) reacted with DBCO-G2(Glu)-$(NHNHFmoc)_4$ (375 mg, 229 μmol) in DMSO (25 mL) for 30 h. The product, $mPEG_{5k}$-G2(Glu)-$(NHNHFmoc)_4$, was purified by dialysis (MWCO: 2 kDa) and characterized by ^{1}H NMR. Deprotection with diethylamine (589 μL, 5.71 mmol) in DCM yielded $mPEG_{5k}$-G2(Glu)-$(NHNH_2)_4$ (423.2 mg). Similar procedures were used for $mPEG_{5k}$-$(NHNH_2)_2$ and mPEG-5k-$NHNH_2$.

c. Synthesis of PEG-Dendron-EPI Prodrugs

mPEG-G2(Glu)-$(NHNH_2)_4$ (300 mg) reacted with EPI·HCl (255 mg) in 0.1 M ammonium acetate buffer (pH 5.0) for 48 h. The product, mPEG-G2(Glu)-$(EPI)_4$ (PEG-Dendron-EPI), was purified by dialysis (MWCO: 2 kDa) and lyophilized. The EPI loading content was determined by UV-Vis spectroscopy (Table S1). PEG-EPI and PEG-Glu-EPI were synthesized via a similar procedure.

1.2.3 Determination of Critical Micelle Concentration (CMC) for Polymeric Prodrugs

The CMC of the polymeric prodrugs was determined using pyrene as a fluorescent probe. Briefly, 200 μL of a pyrene-acetone solution (0.67×10^{-6} M) was added to a 10 mL glass vial. After complete evaporation of acetone, an aqueous solution of the polymeric prodrug at varying concentrations (0.03 to 1000 μg/mL) was added to the vial. The mixture was incubated at room temperature for 2 h to reach equilibrium. Fluorescence emission spectra were recorded using a fluorescence spectrophotometer

(excitation wavelength: 335 nm; emission wavelength range: 350-450 nm). The intensity ratio of the first (I1, 373 nm) to the third (I3, 384 nm) vibronic peaks in the pyrene spectrum was calculated. The I1/I3 ratio was plotted against the logarithm of the polymer concentration, and the CMC value was determined as the intersection point of two linear regressions.

1.3 Simulation of nanoparticle formation and drug loading based on polymer prodrug

1.3.1 Dissipative particle dynamics simulations

We constructed a coarse-grained (CG) model to describe the experimental system for dissipative particle dynamics [1] mesoscopic simulation. The interaction parameter α_{ij} is a constant which describes the maximum repulsion between interacting beads. The coarse-grained scheme for this system was shown in Figure S27. In this model, three molecules of water were treated as one bead.

The interaction parameter α_{ij} between beads was mapped to the Flory-Huggins χ parameter through $\Delta\alpha_{ij} = 3.27\chi_{ij}$ [2]. The parameter χ can be calculated by the following equation:

$$\chi_{ij} = \frac{V_{ij}}{RT}(\delta_i - \delta_j)^2$$

Here, V_{ij} is is the average molar volume of the bead, R is the gas constant, T is the absolute temperature, and δ_i and δ_j are the solubility parameters for the chemical entities i and j, which were calculated via the Materials Studio Program (Accelrys Inc.).

The temporal evolution of interacting beads was trajectorized by following the Newton's equations of motion [3]. Canonical ensemble simulations were performed in a three-dimensional box with a size of 40^3. The bead number density ρ was defined as 3, and the integration time step Δt was set at 0.01.

Two different processes were simulated, including the self-assembly of polymers and the engulfment of drugs. In the simulation of drug engulfment, each polymer was separately simulated to engulf drugs with a mass fraction of 5%, 10% or 20% [4]. The details of this model can be found in a previous study by Liu et al. in 2007 [5].

1.3.2 All-atom molecular dynamics simulation (AAMD)

All-atom molecular dynamics simulation (AAMD) was used to reveal the mechanism of engulfing EPI by polymers at different amounts from a microscopic perspective. The adsorption interaction between polymers with different percentages of

EPI grafting and drug molecules was calculated.

The Materials Studio software was employed to perform molecular dynamics simulations [6]. Condensed-phase Optimized Molecular Potentials for Atomistic Simulation Studies (COMPASS II) [7] were applied to model three types of EPI prodrugs and TPP-LND. Initial system optimization was achieved through the steepest descent minimization algorithm. Canonical ensemble (*NVT*) stabilization was performed until the total energy of the system was converged to a predefined threshold [8]. Subsequent *NVT* ensemble simulations spanning 3 ns were executed for all configurations to ensure reaching an equilibrium. The final frame structure was selected to independently assess the total system energy and subsystem energies for adsorption energy determination. A 1-fs timestep was used for these simulations at 298 K, enabling precise characterization of molecular dynamics [9]. Both electrostatic and van der Waals interactions were truncated at a cut-off distance of 1.8 nm [10]. Visualization and analysis was performed via the Visual Molecular Dynamics (VMD) software (version 1.9.2) [11]. The calculation formula of the adsorption energy was shown below:

$$E_{ads} = E_{total} - E_{adsorbate} - E_{adsorbent}$$

2. Supplementary results

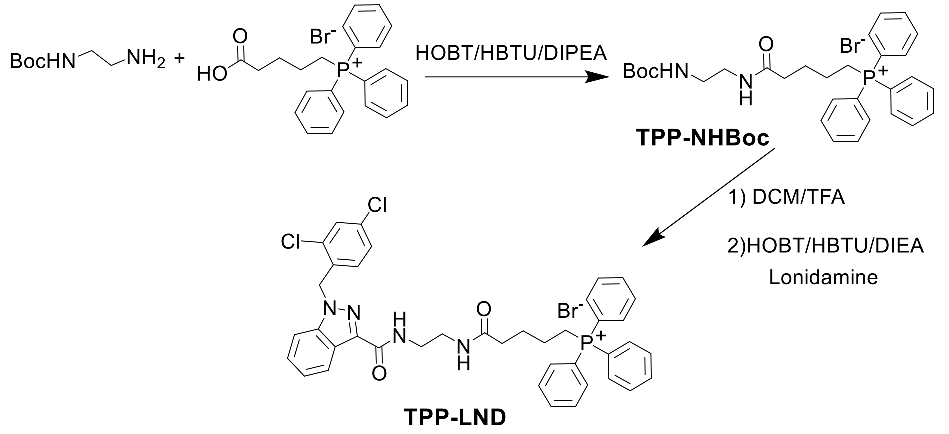

Scheme S1. The synthetic route and the chemical structure of the mitochondria-targeting small-molecule compound TPP-LND.

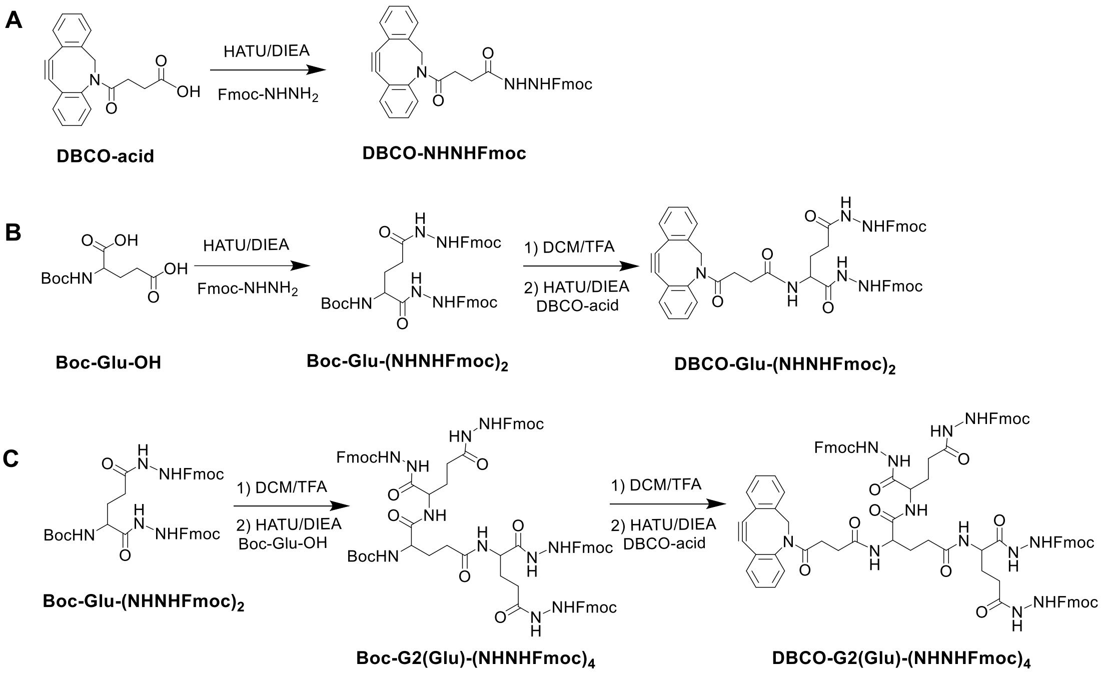

Scheme S2. Synthesis routes and chemical structures of small molecule compounds modified with DBCO.

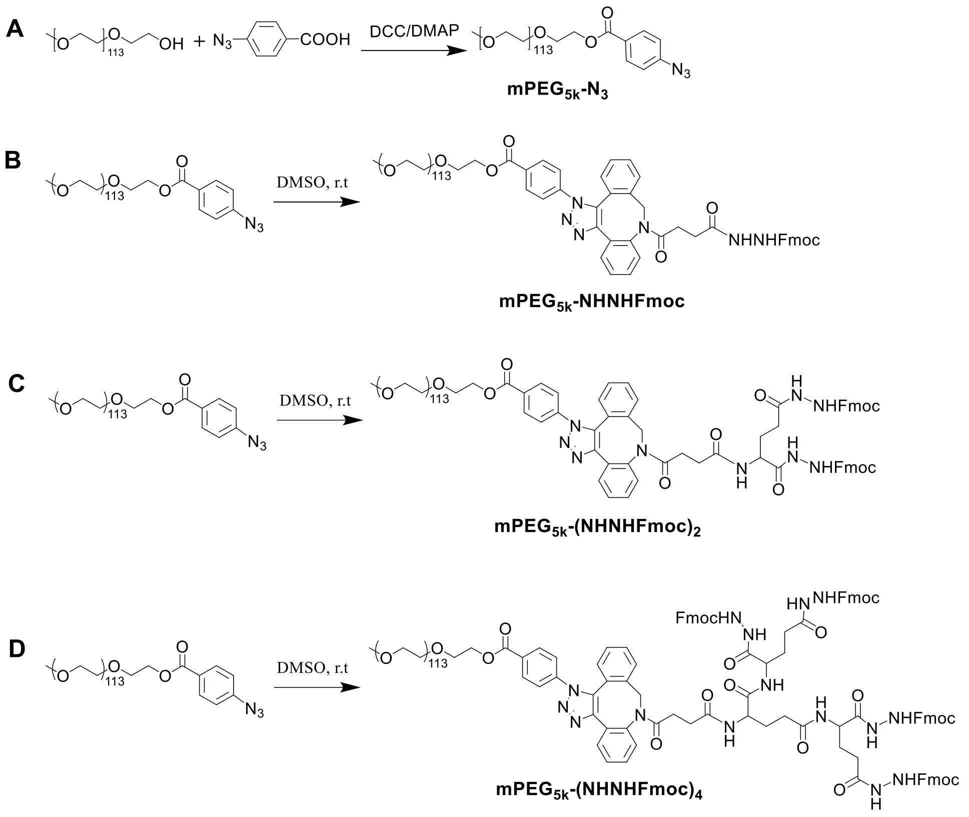

Scheme S3. Synthetic routes and chemical structures of polymer intermediates based on mPEG$_{5k}$.

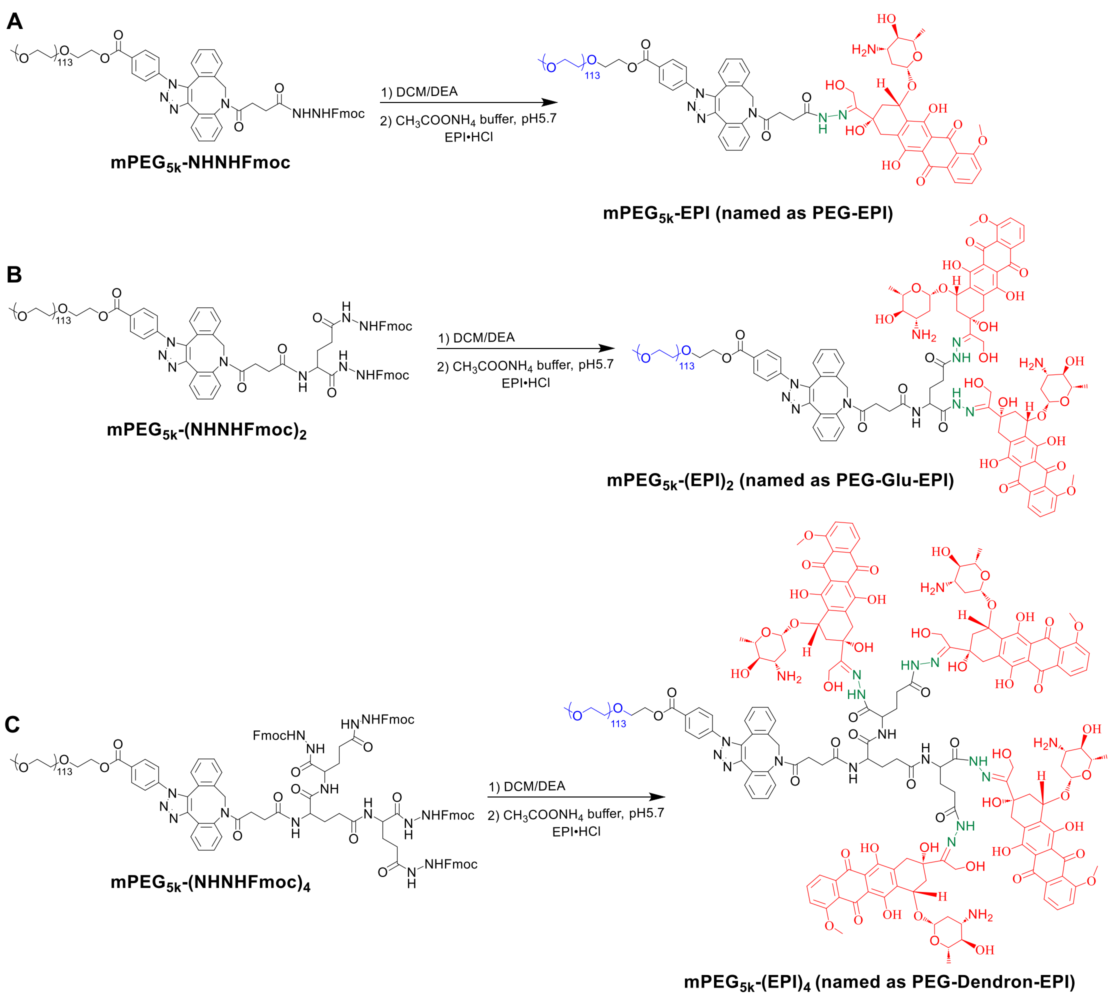

Scheme S4. The preparation routes and structural diagrams of PEG-EPI polymer prodrugs with different structures.

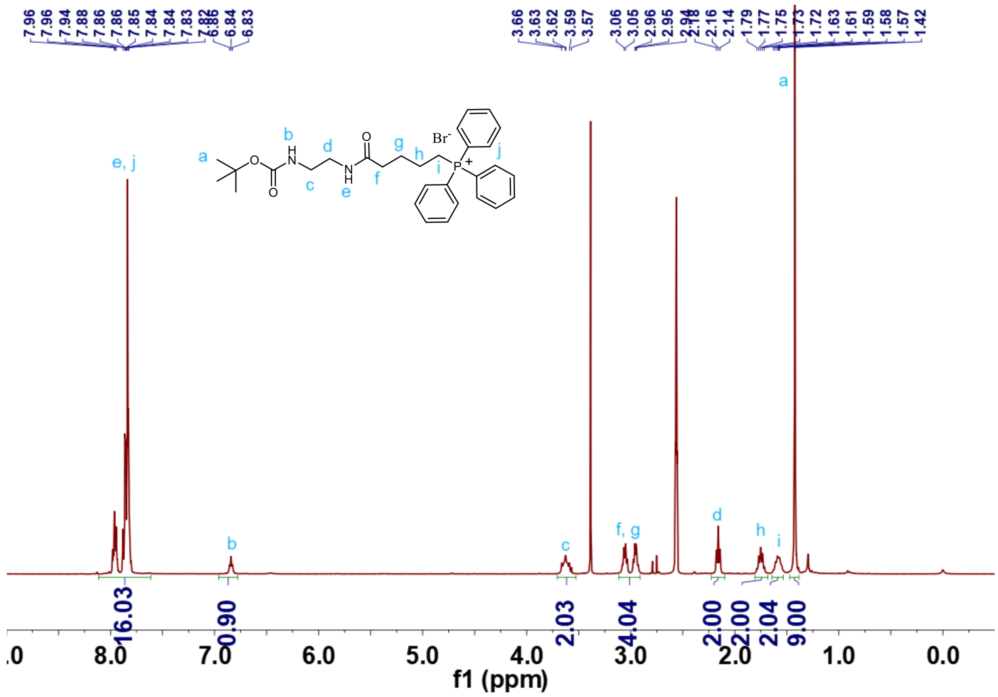

Figure S1. ^{1}H NMR spectrum of TPP-NHBoc in DMSO-*d6*.

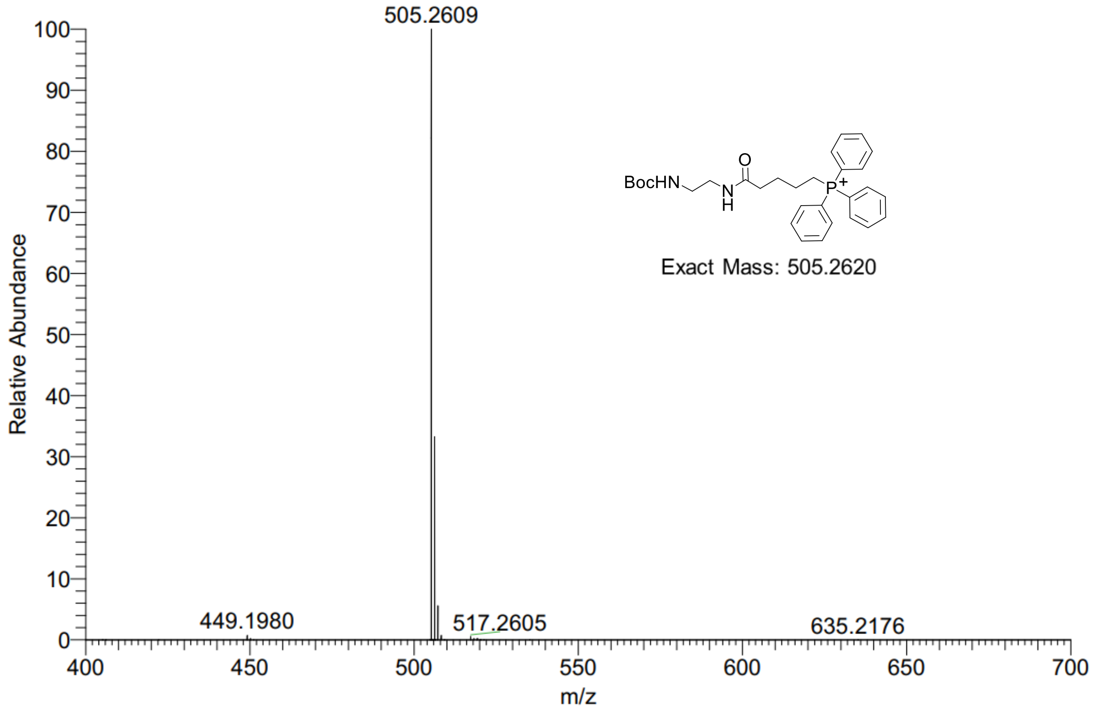

Figure S2. HRMS spectrum of TPP-NHBoc.

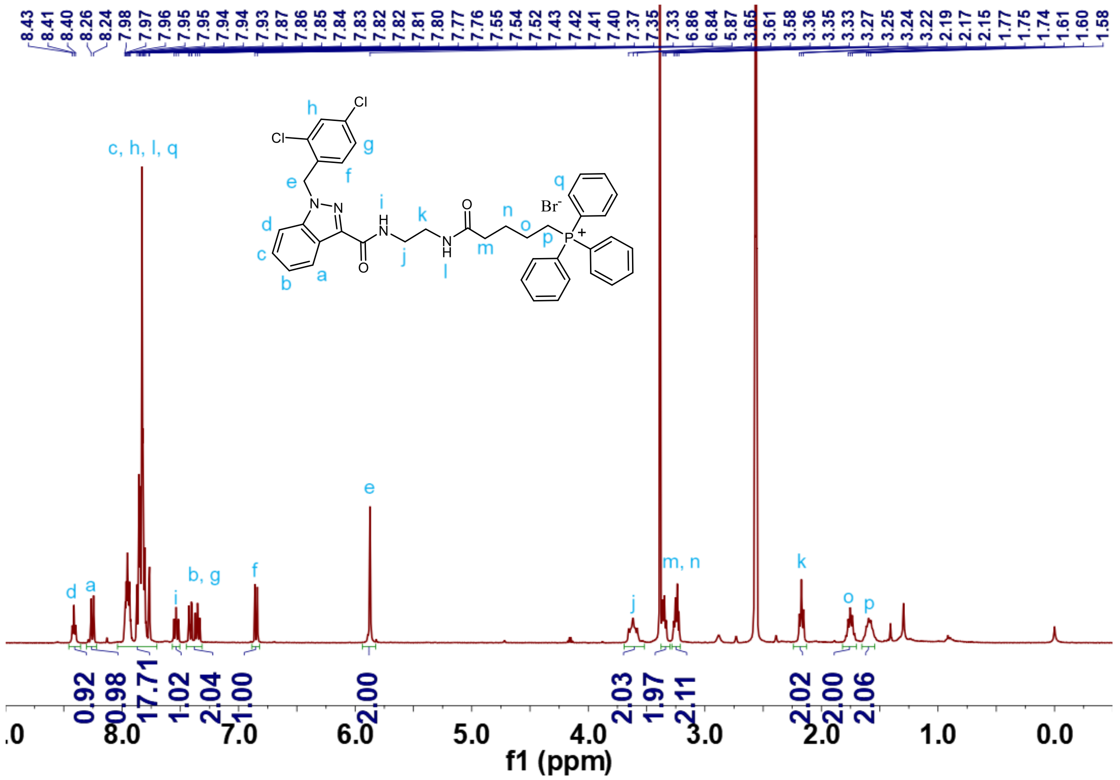

Figure S3. 1H NMR spectrum of TPP-LND in DMSO-d6.

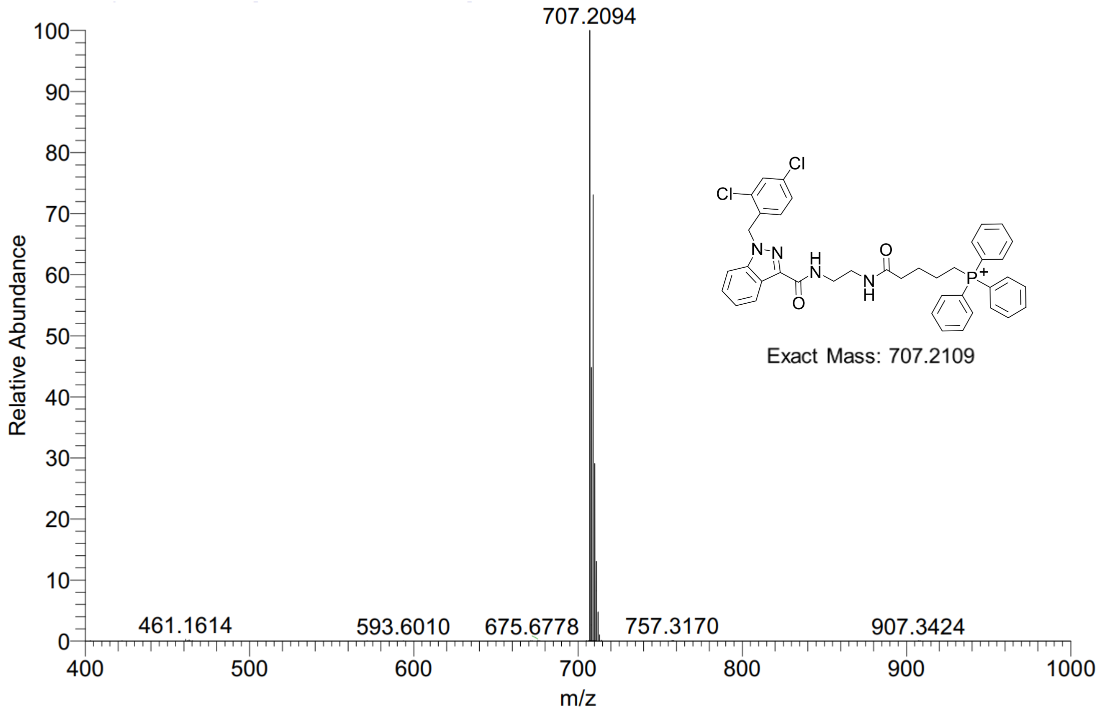

Figure S4. HRMS spectrum of TPP-LND.

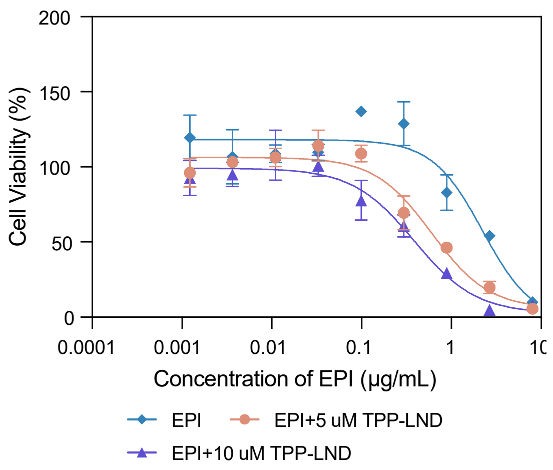

Figure S5. The viability of 4T1 cells treated with EPI and TPP-LND at a TPP-LND concentration of 0, 5 or 10 μM with a gradient concentration of EPI for 48 h.

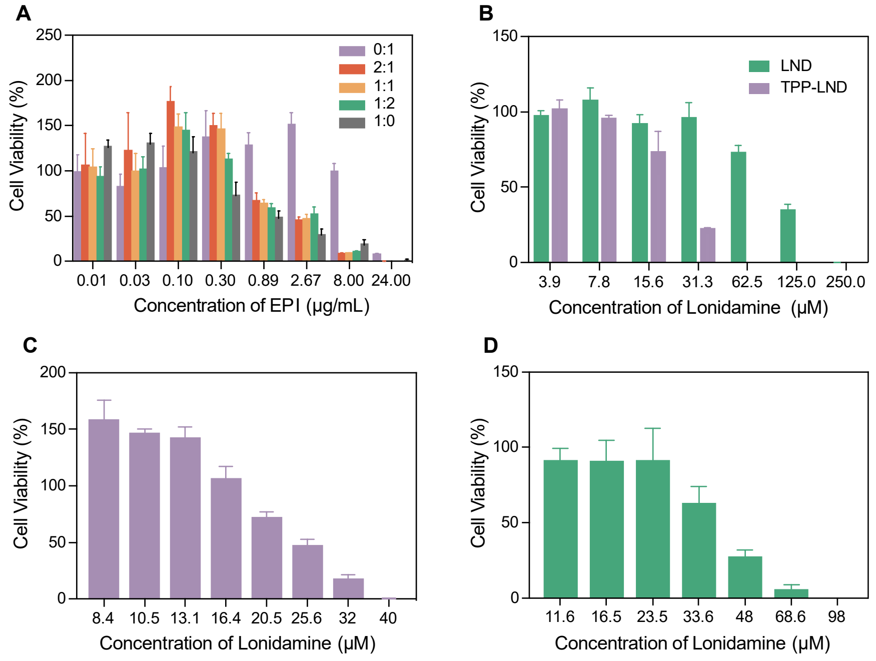

Figure S6. (A) The viability of 4T1 cells treated with a physical mixture of EPI and TPP-LND at different ratios (EPI / TPP-LND) for 48 h. (B) The cytotoxicity of LND and TPP-LND at different concentrations of Loinidamine against 4T1 cells at 24 h after treatment. The viability of 4T1 cells treated with (C) LND and (D) TPP-LND at different concentrations of Loinidamine at 48 h.

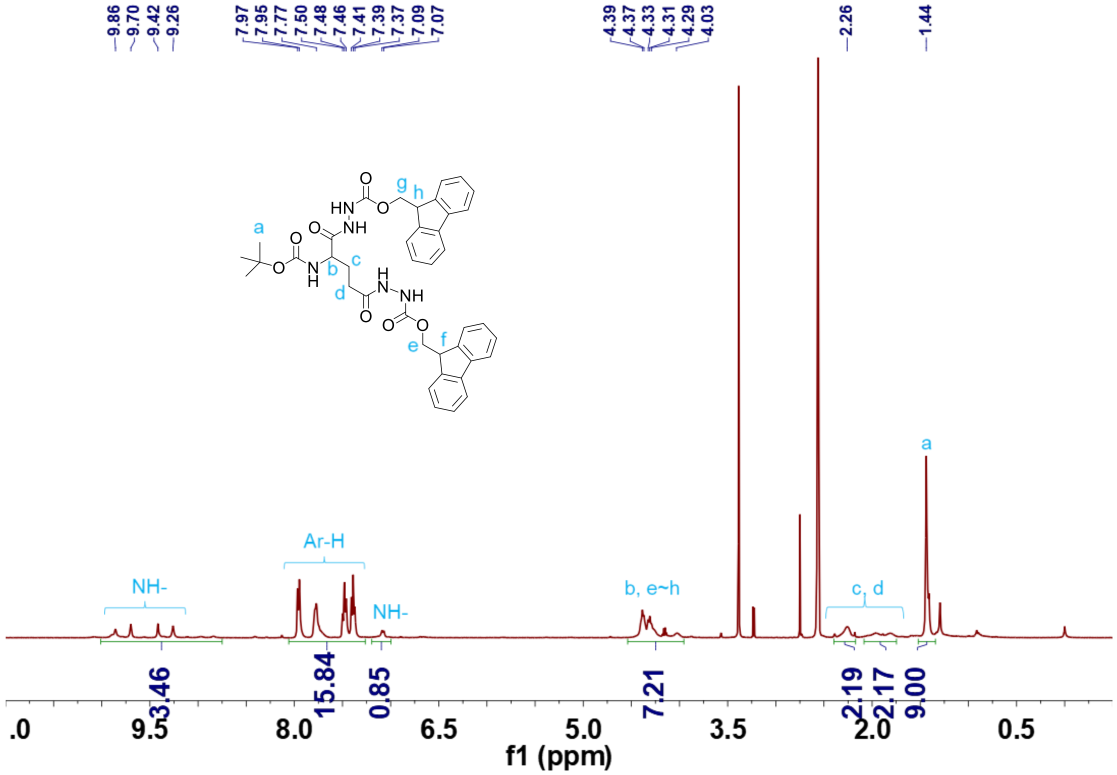

Figure S7. ^{1}H NMR spectrum of Boc-Glu-(NHNHFmoc)$_2$ in DMSO-*d6*.

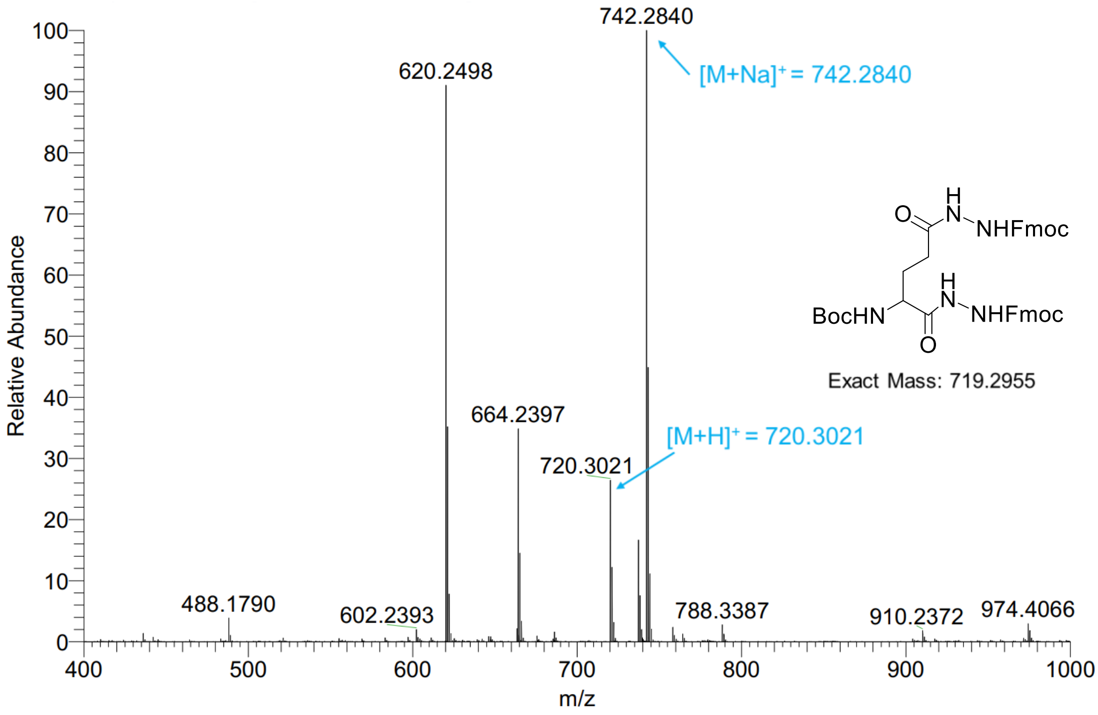

Figure S8. HRMS spectrum of Boc-Glu-(NHNHFmoc)$_2$.

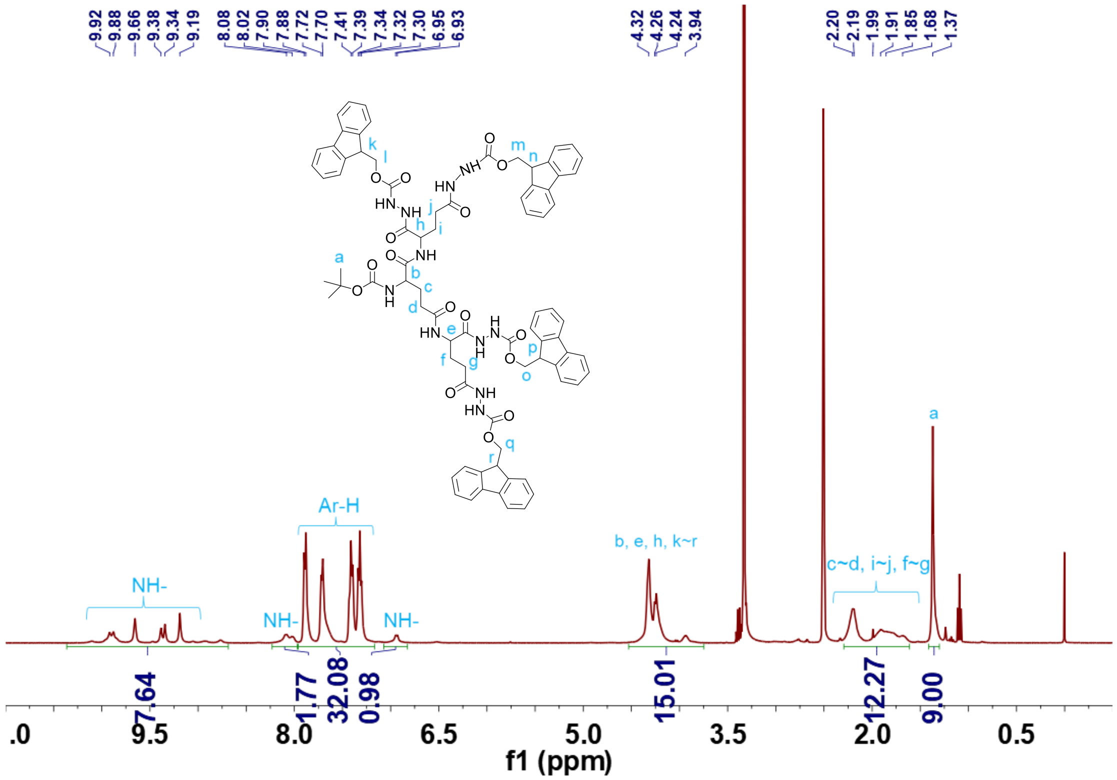

Figure S9. ^{1}H NMR spectrum of Boc-G2(Glu)-(NHNHFmoc)$_4$ in DMSO-*d6*.

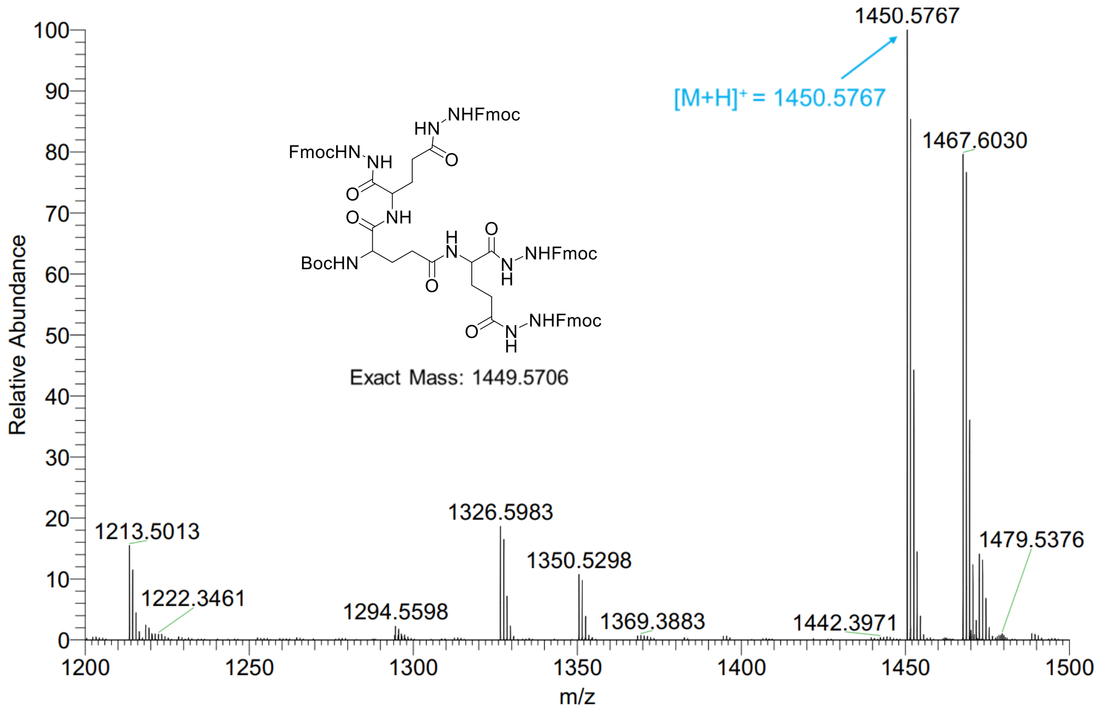

Figure S10. HRMS spectrum of Boc-G2(Glu)-(NHNHFmoc)$_4$.

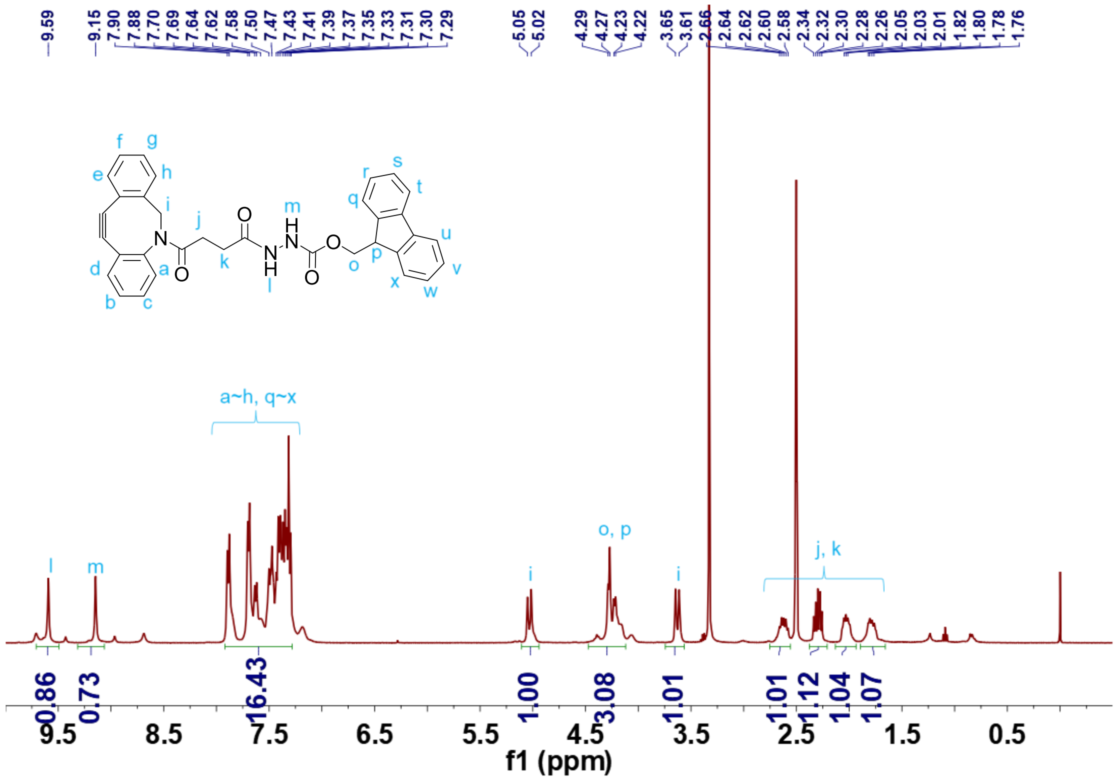

Figure S11. ^{1}H NMR spectrum of DBCO-NHNHFmoc in DMSO-*d6*.

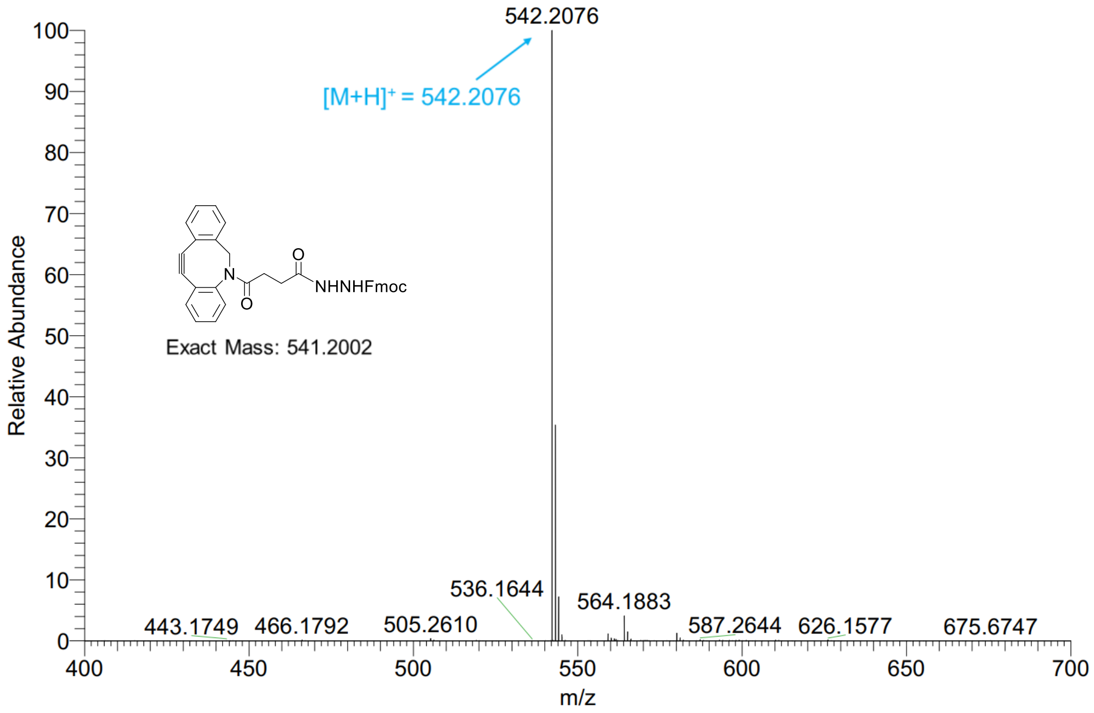

Figure S12. HRMS spectrum of DBCO-NHNHFmoc.

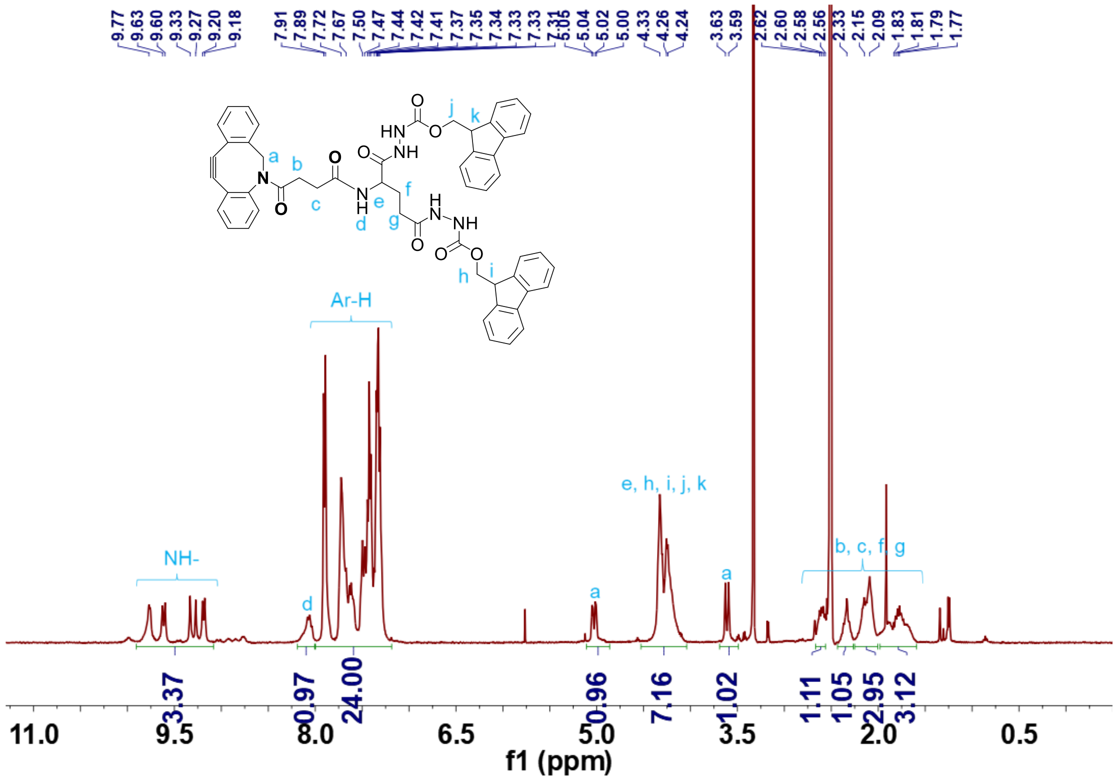

Figure S13. ^{1}H NMR spectrum of DBCO-(NHNHFmoc)$_2$ in DMSO-*d6*.

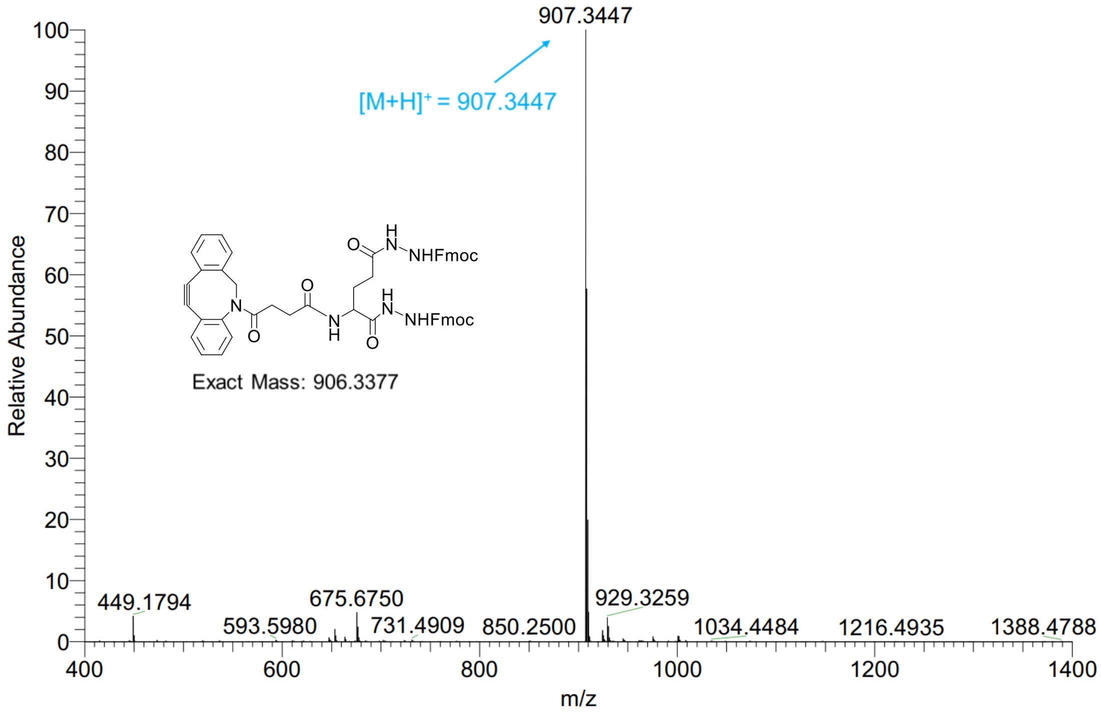

Figure S14. HRMS spectrum of DBCO-(NHNHFmoc)$_2$.

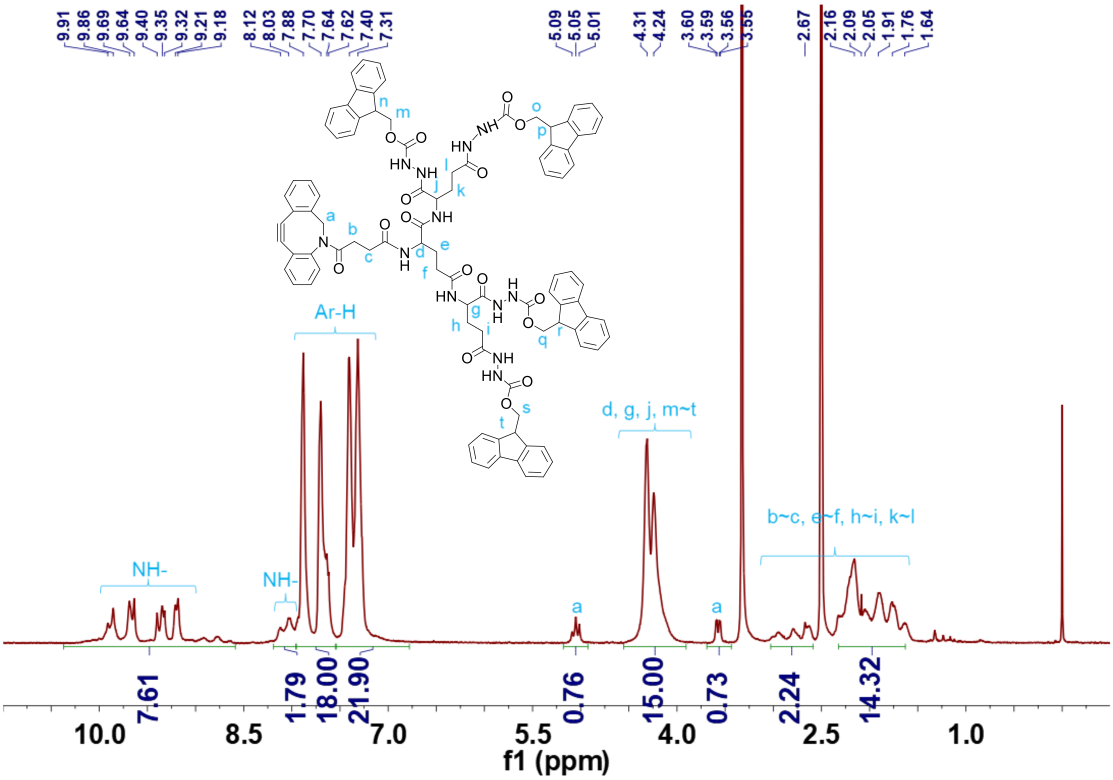

Figure S15. ^{1}H NMR spectrum of DBCO-(NHNHFmoc)$_4$ in DMSO-*d6*.

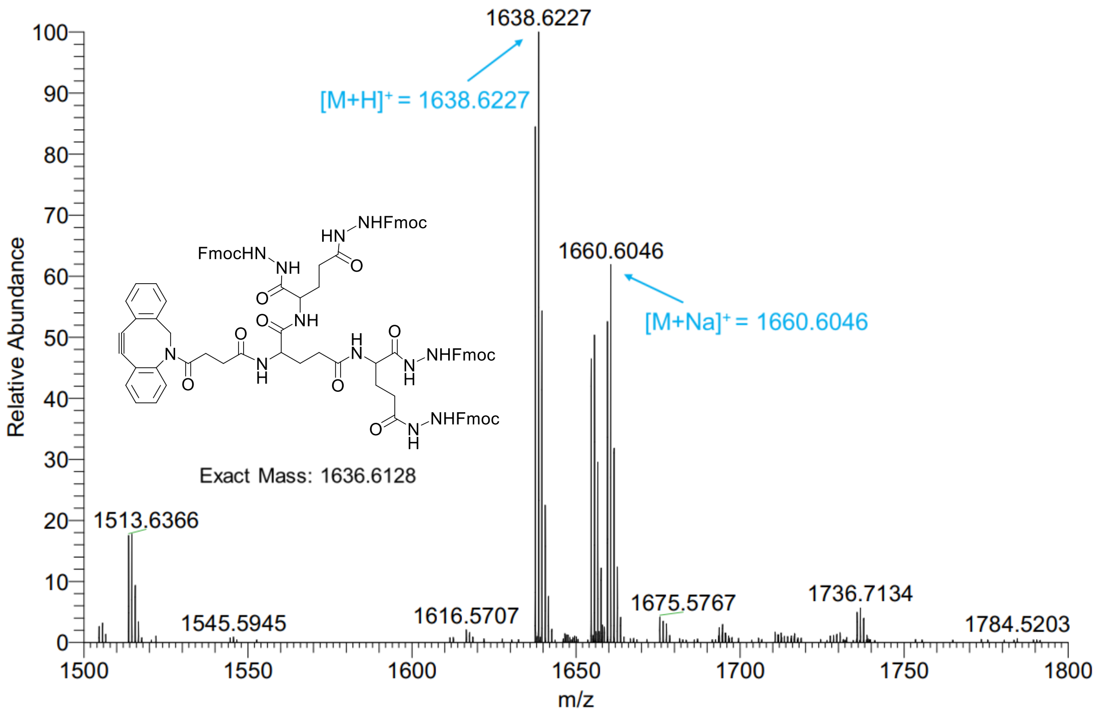

Figure S16. HRMS spectrum of DBCO-(NHNHFmoc)$_4$.

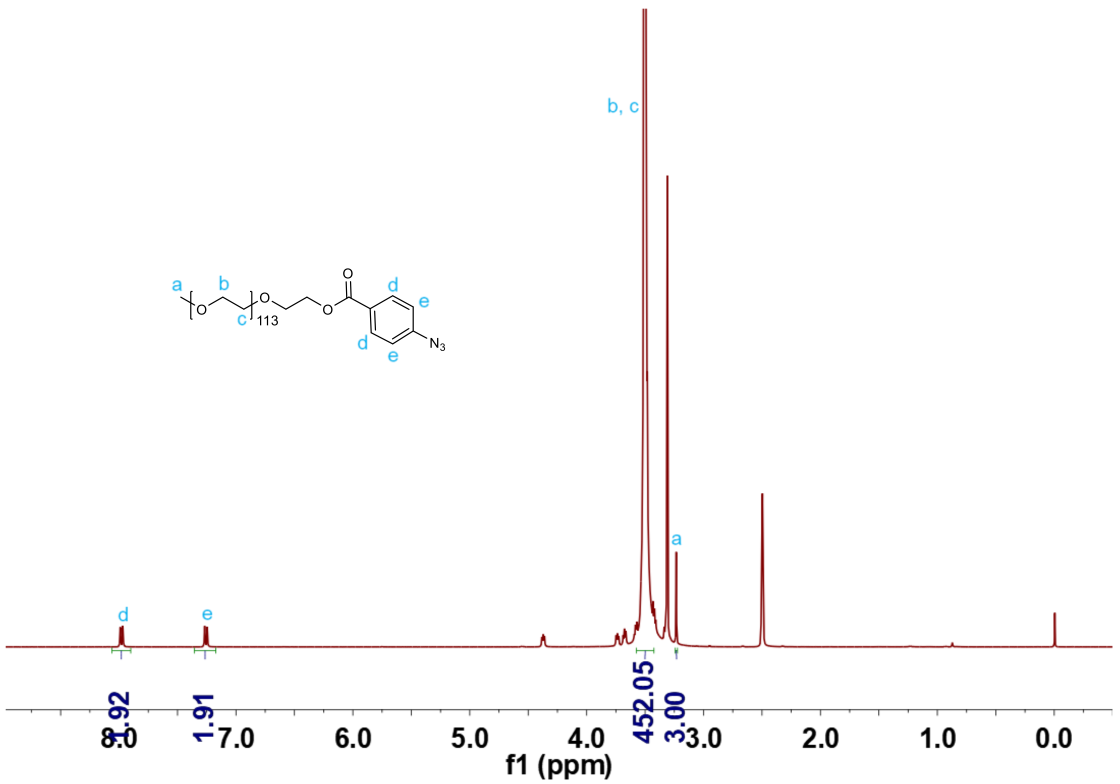

Figure S17. ^{1}H NMR spectrum of mPEG$_{5k}$-N_3 in DMSO-*d6*.

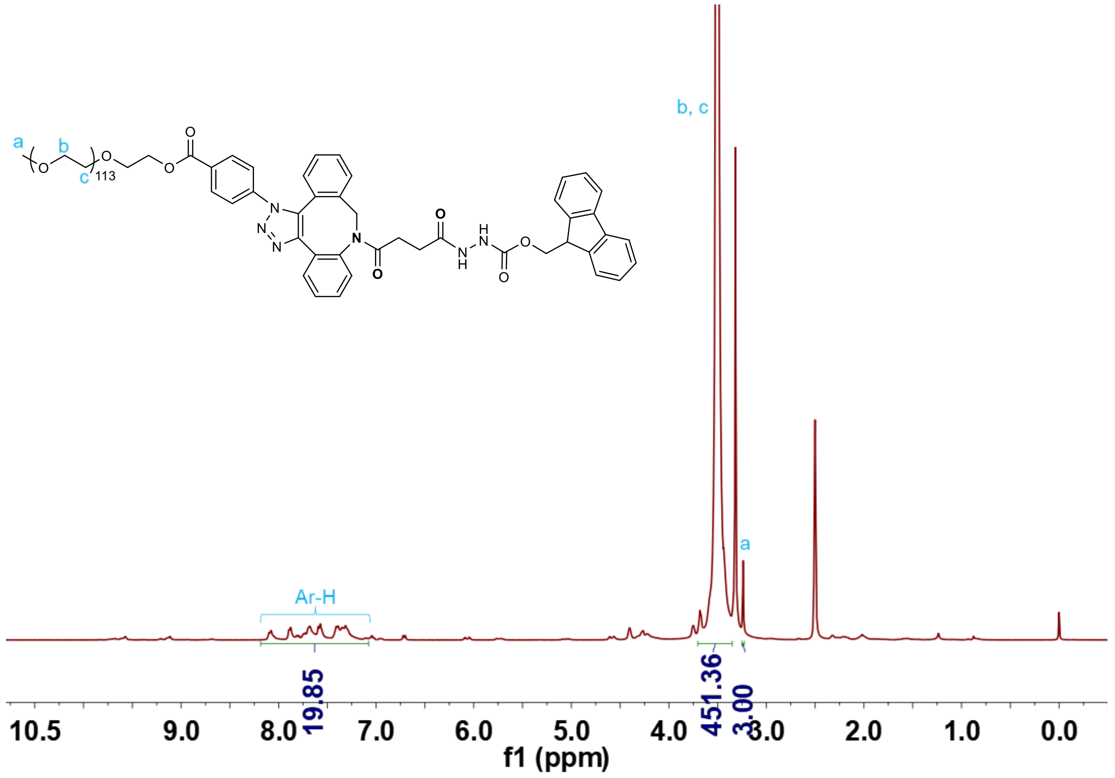

Figure S18. ^{1}H NMR spectrum of mPEG$_{5k}$-NHNHFmoc in DMSO-*d6*.

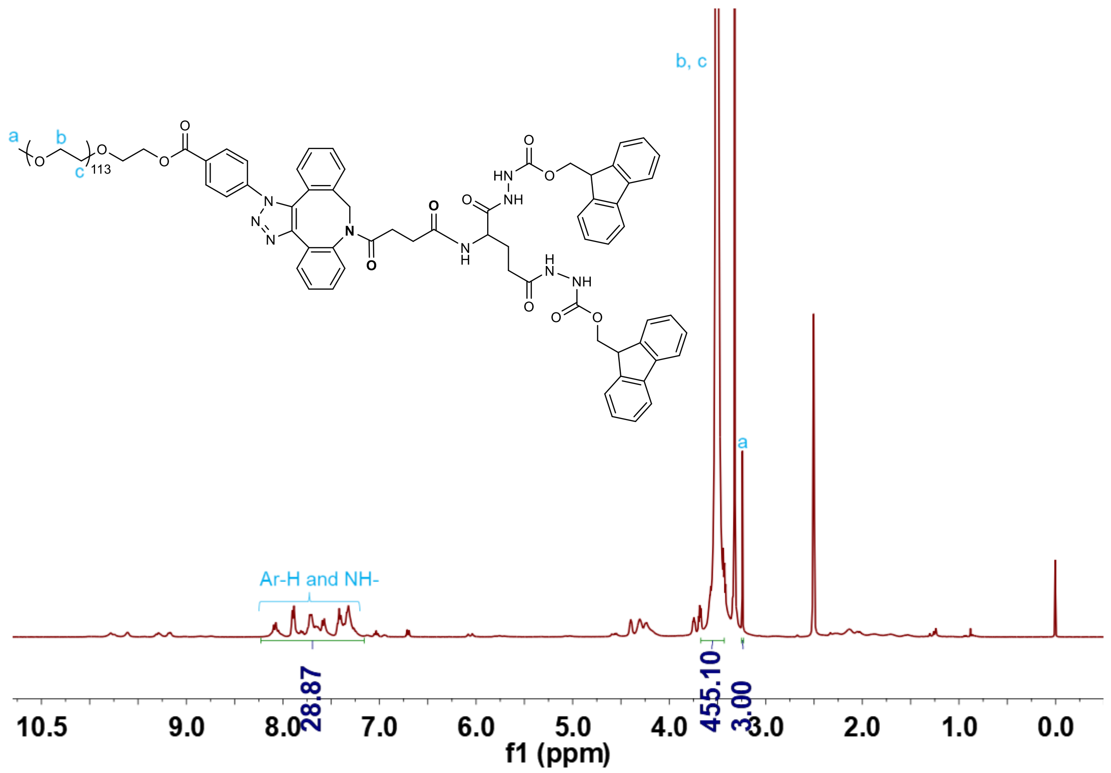

Figure S19. ^{1}H NMR spectrum of mPEG$_{5k}$-(NHNHFmoc)$_2$ in DMSO-*d6*.

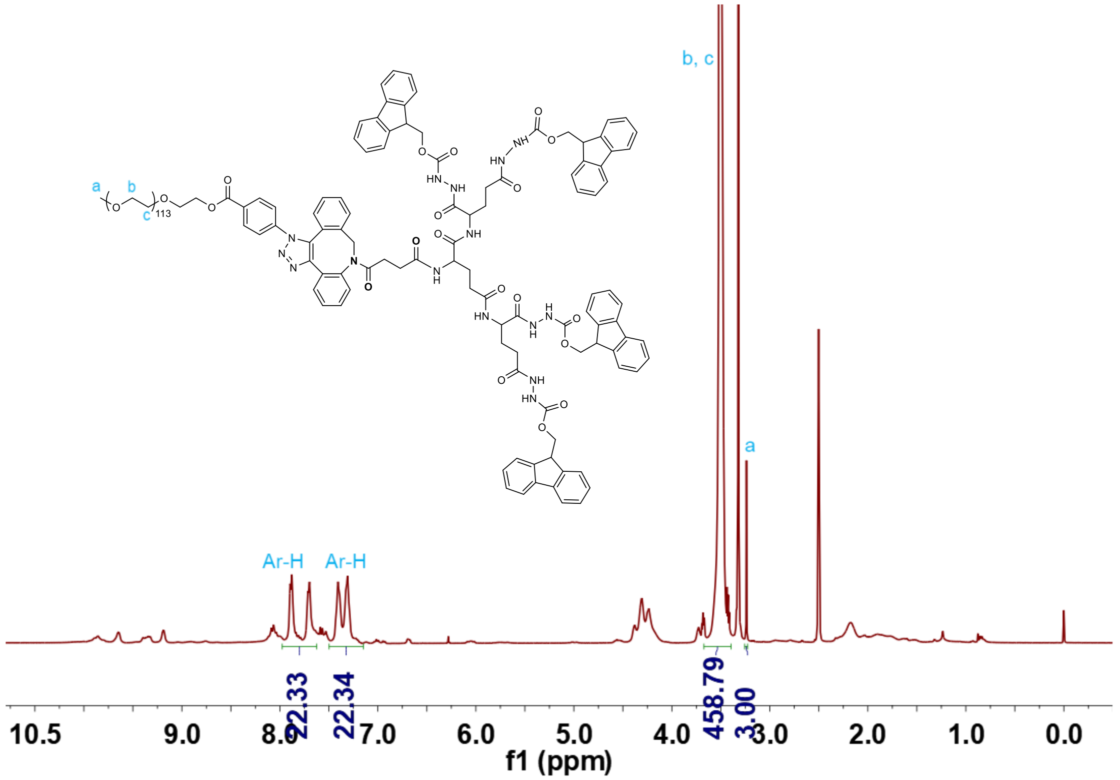

Figure S20. ^{1}H NMR spectrum of mPEG$_{5k}$-(NHNHFmoc)$_4$ in DMSO-*d6*.

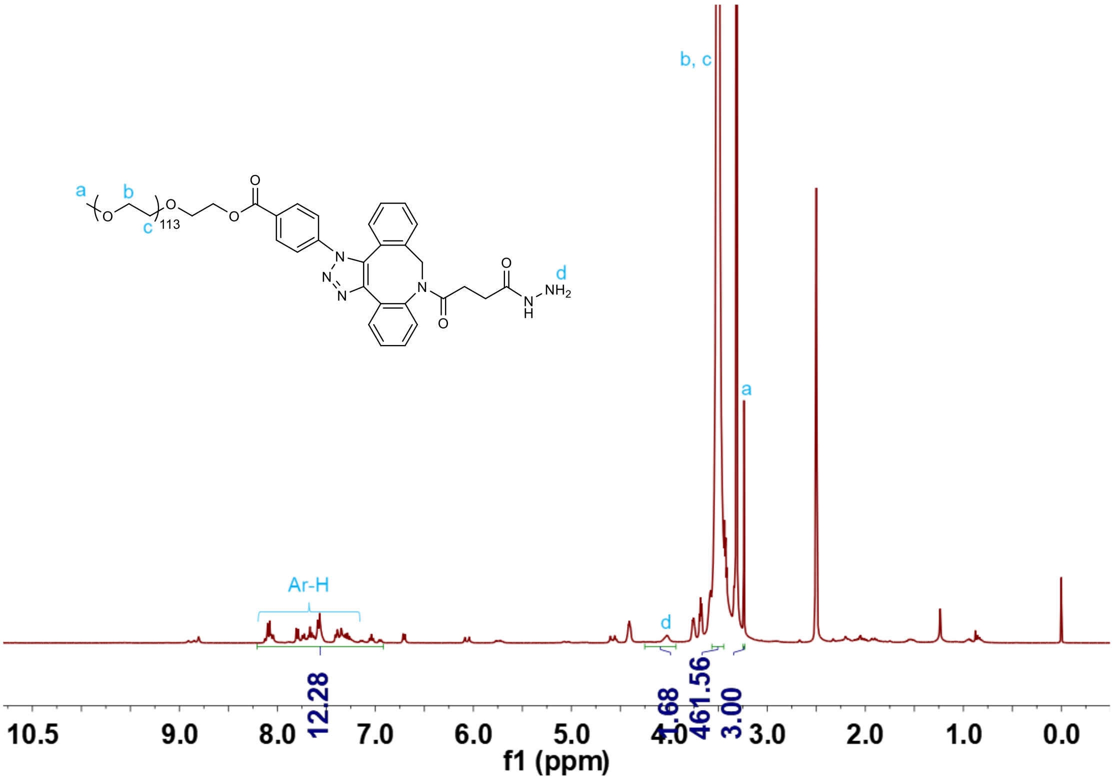

Figure S21. ^{1}H NMR spectrum of mPEG$_{5k}$-NHNH$_2$ in DMSO-*d6*.

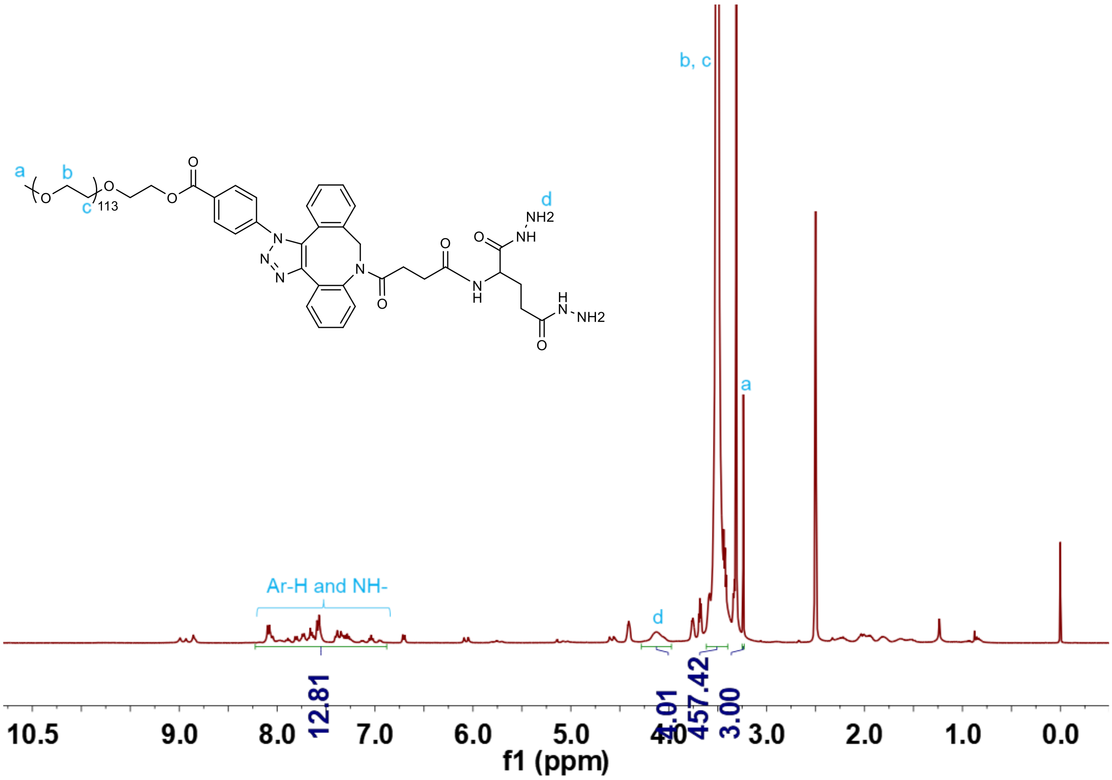

Figure S22. ^{1}H NMR spectrum of mPEG$_{5k}$-(NHNH$_2$)$_2$ in DMSO-*d6*.

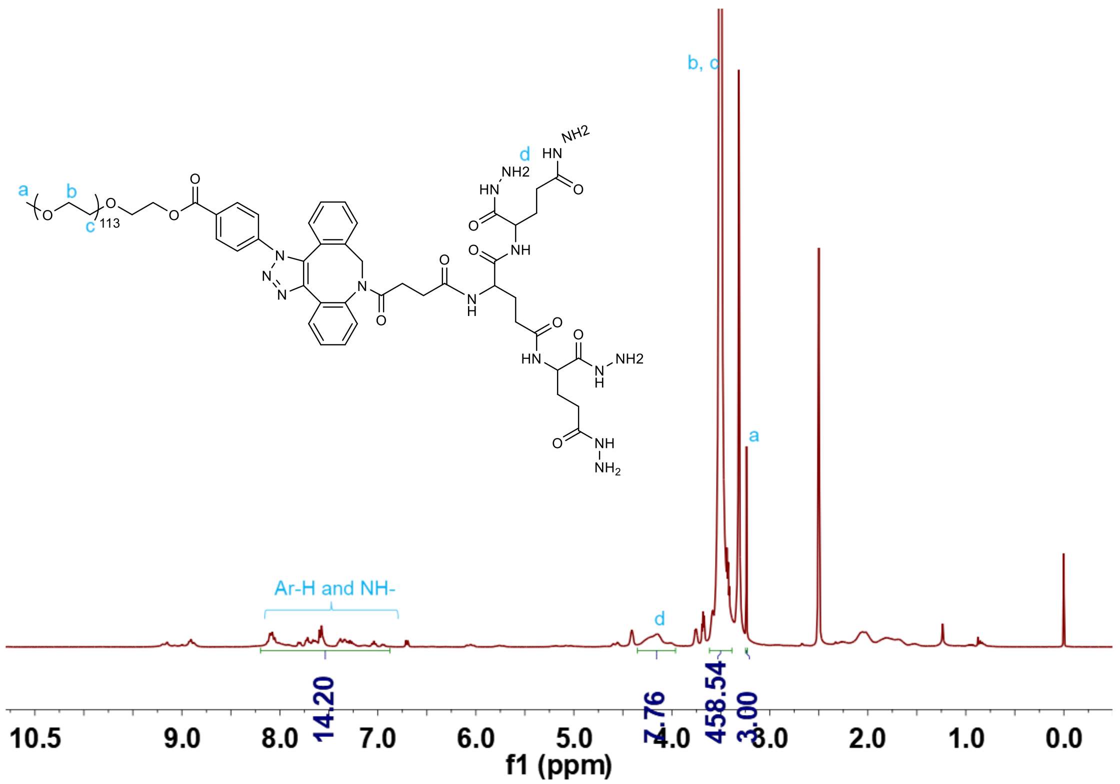

Figure S23. ^{1}H NMR spectrum of mPEG$_{5k}$-$(NHNH_2)_4$ in DMSO-*d6*.

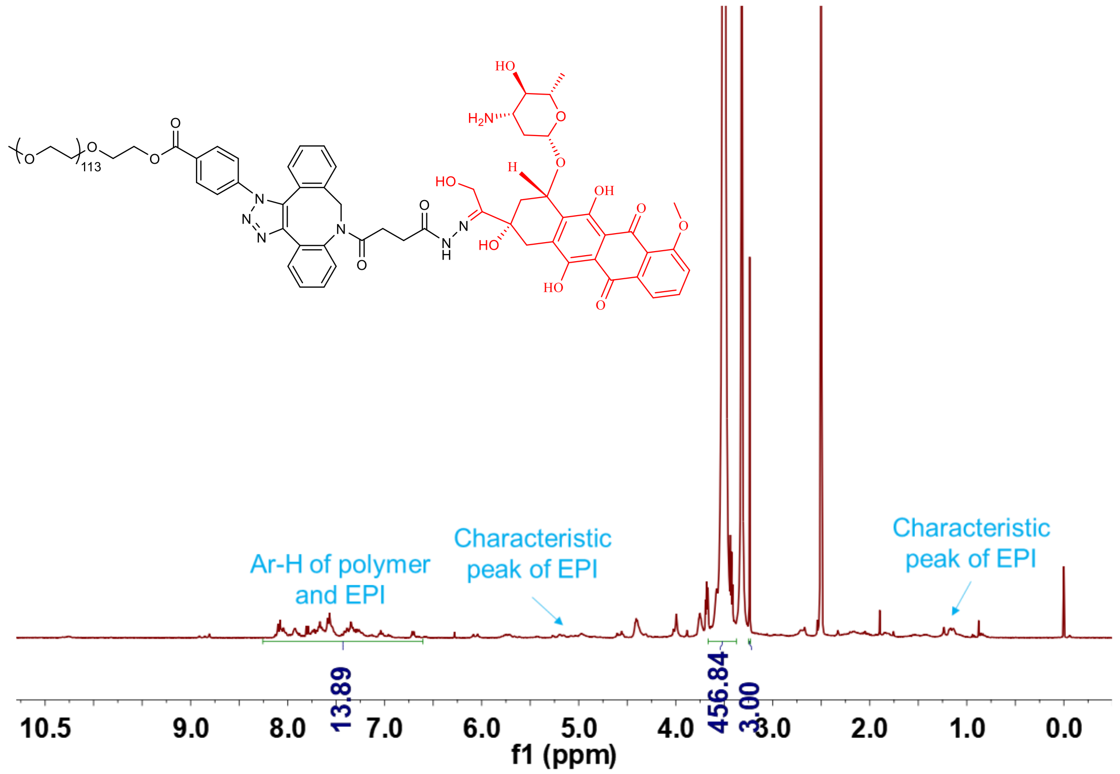

Figure S24. ^{1}H NMR spectrum of PEG-EPI in DMSO-*d6*.

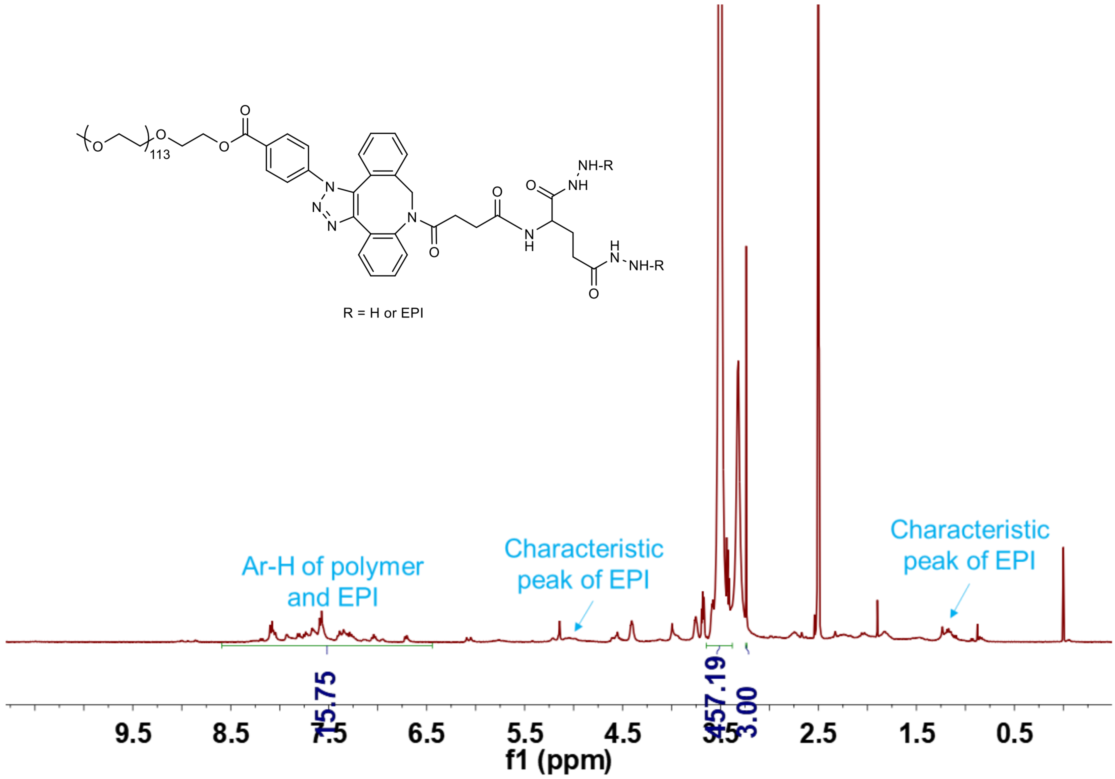

Figure S25. ^{1}H NMR spectrum of PEG-Glu-EPI in DMSO-*d6*.

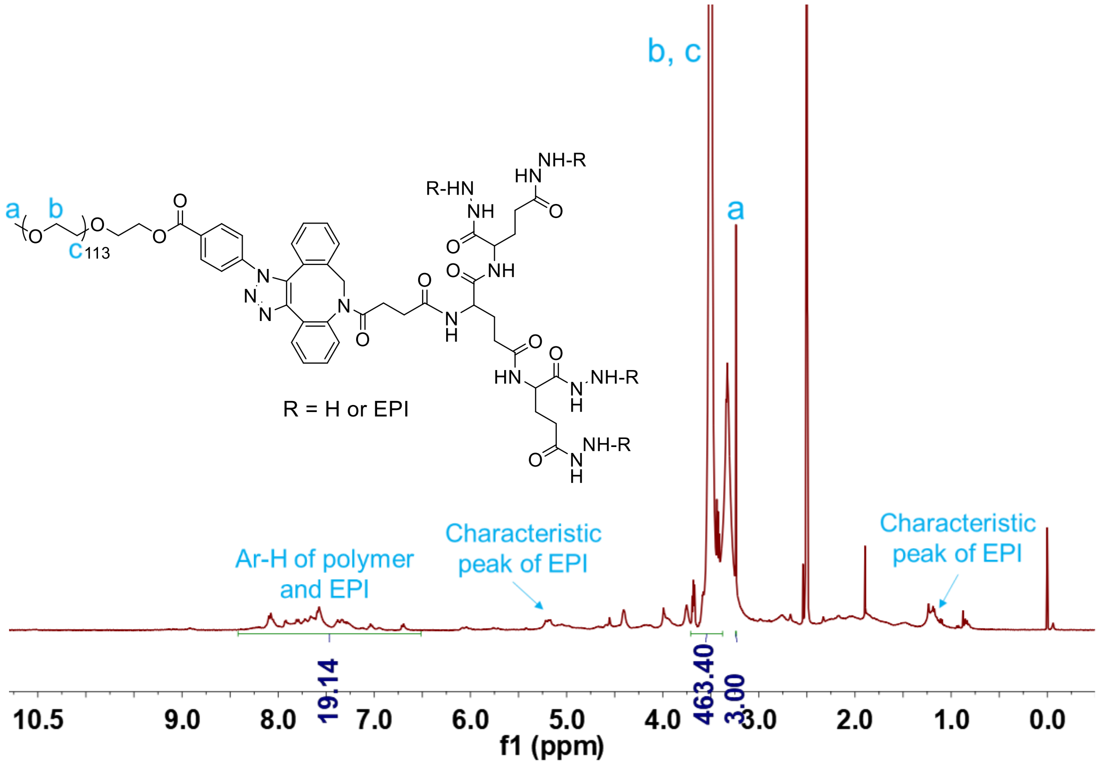

Figure S26. ^{1}H NMR spectrum of PEG-Dendron-EPI in DMSO-*d6*.

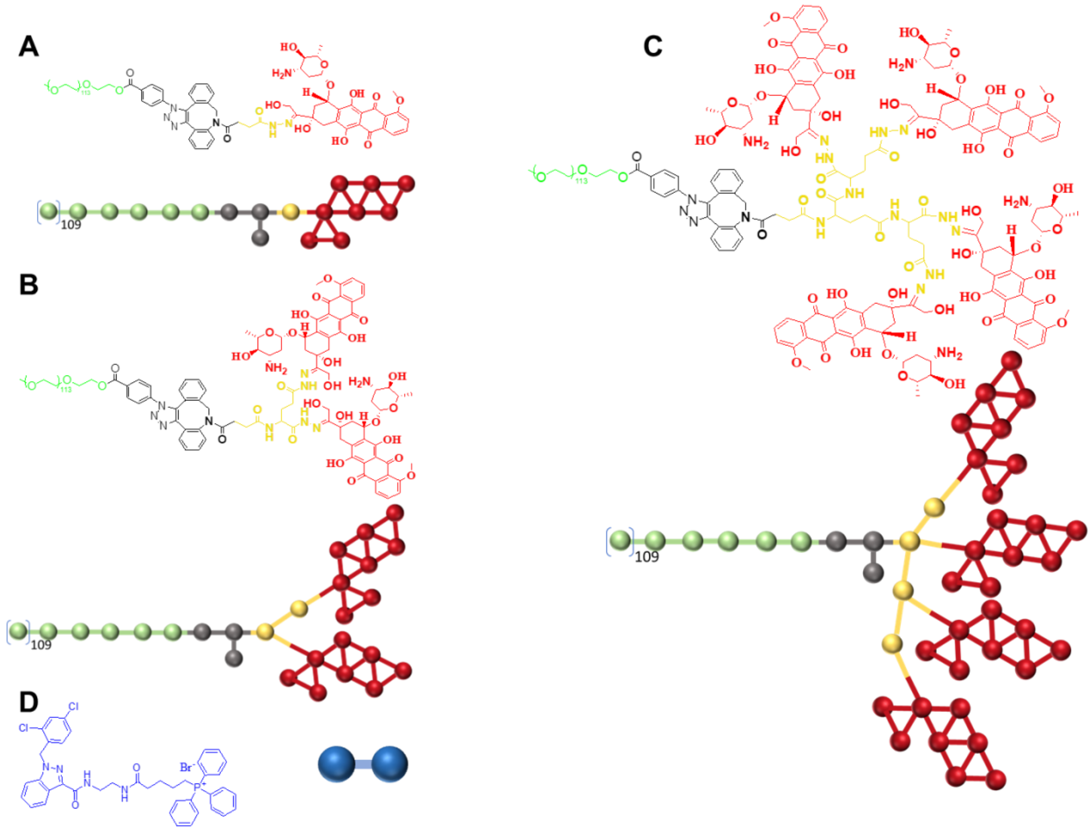

Figure S27. Structural representations and CG models of polymeric prodrugs and small molecule drugs. A-C: dissipative particle dynamics (DPD) mappings of polymeric prodrugs PEG-EPI, PEG-Glu-EPI, and PEG-Dendron-EPI, respectively. D: mapping of the mitochondria-targeting small-molecule drug TPP-LND. In the DPD models, red beads for the EPI moieties; yellow and black beads for the linker segments connecting hydrophobic and hydrophilic blocks; green beads for hydrophilic segments; and blue beads for the TPP-LND drug molecule.

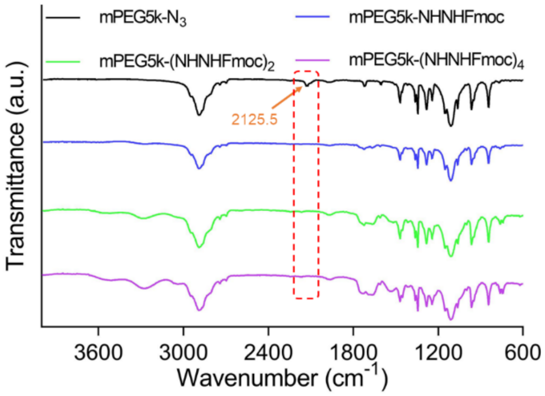

Figure S28. Infrared spectra of mPEG$_{5k}$-N$_3$, mPEG$_{5k}$-NHNHFmoc, mPEG$_{5k}$-(NHNHFmoc)$_2$ and mPEG$_{5k}$-(NHNHFmoc)$_4$. Compared with mPEG$_{5k}$-N$_3$, the disappearance of the characteristic azide peak at 2125.5 cm^{-1} in the other three polymeric intermediates indicated successful conjugation of DBCO-functionalized small molecules to the PEG terminals.

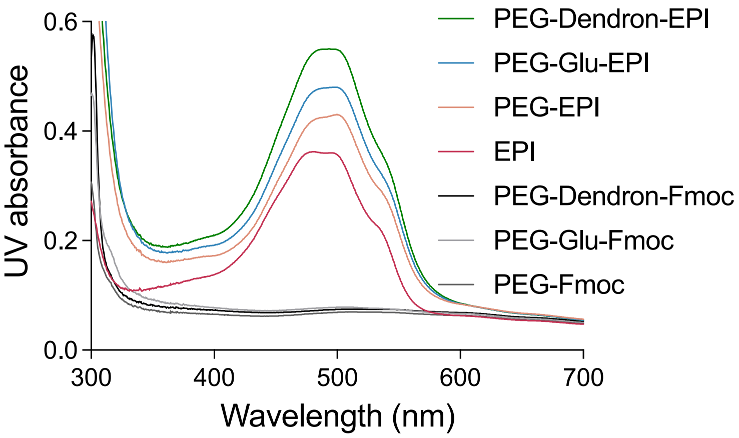

Figure S29. UV-vis absorption spectra of EPI, PEG-EPI , PEG-Glu-EPI and PEG-Dendron-EPI in water. PEG-Fmoc, PEG-Glu-Fmoc and PEG-Dendron-Fmoc were used as controls. The three EPI prodrugs and free EPI displayed similar UV absorption spectra, indicating that coupling EPI to polymers had no significant effect on their optical properties.

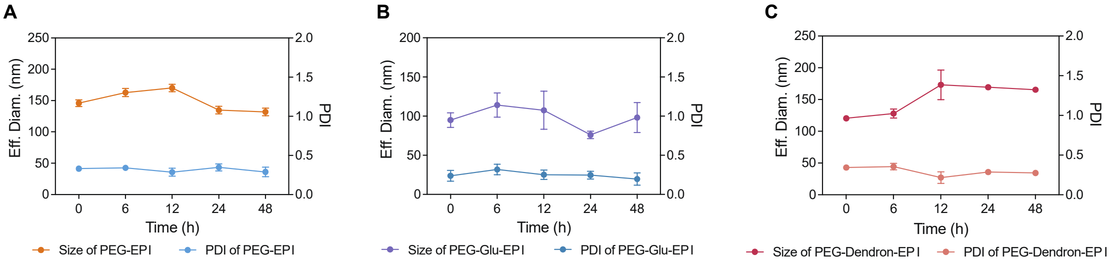

Figure S30. The stability of PEG-EPI (A), PEG-Glu-EPI (B) and PEG-Dendron-EPI (C) in water within 48 h.

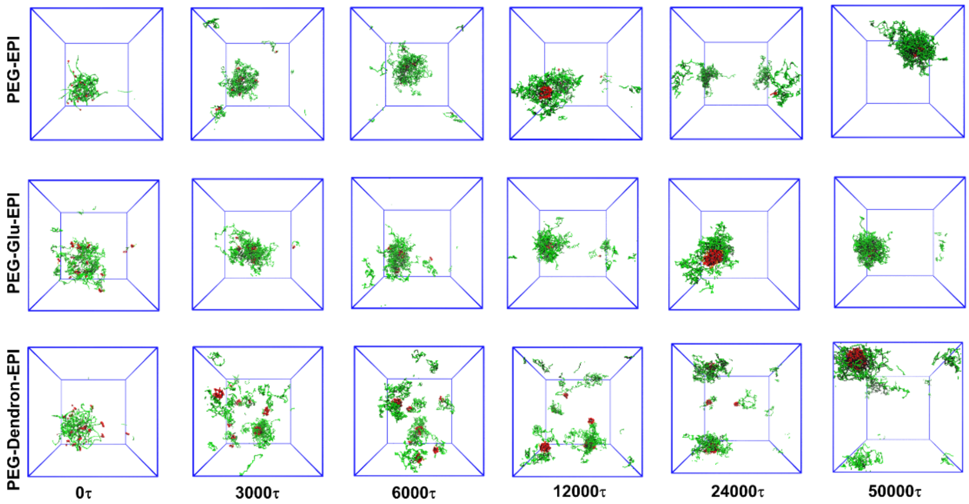

Figure S31. Simulation of nanoparticle formation by PEG-EPI, PEG-Glu-EPI, and PEG-Dendron-EPI in a neutral solvent. The DPD simulations were performed for 50000τ with an integration time step of Δt = 0.01τ.

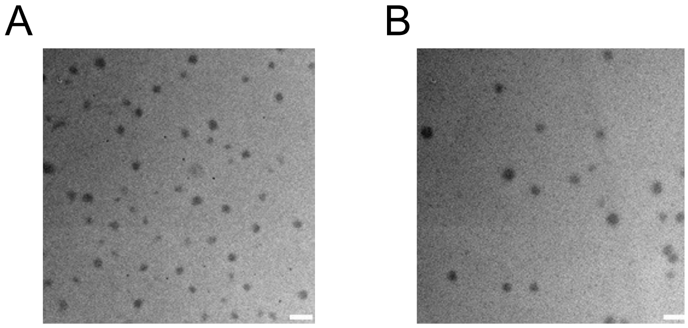

Figure S32. TEM images of PEG-EPI (A) and PEG- Glu-EPI (B) (scale bar = 100 nm).

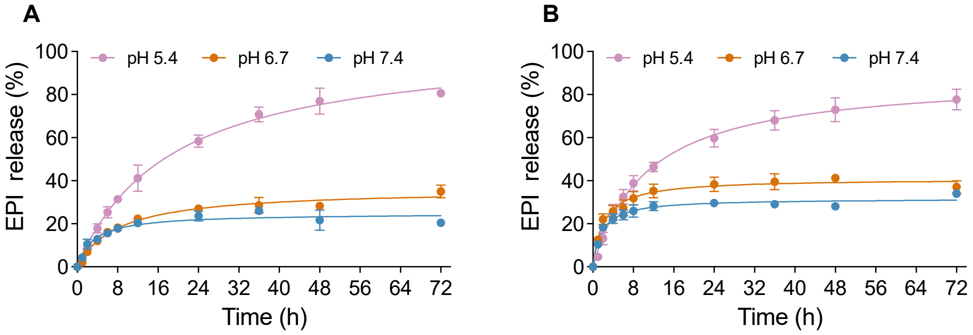

Figure S33. Release curves of EPI from PEG-EPI (A) and PEG-Glu-EPI (B) under different pH conditions.

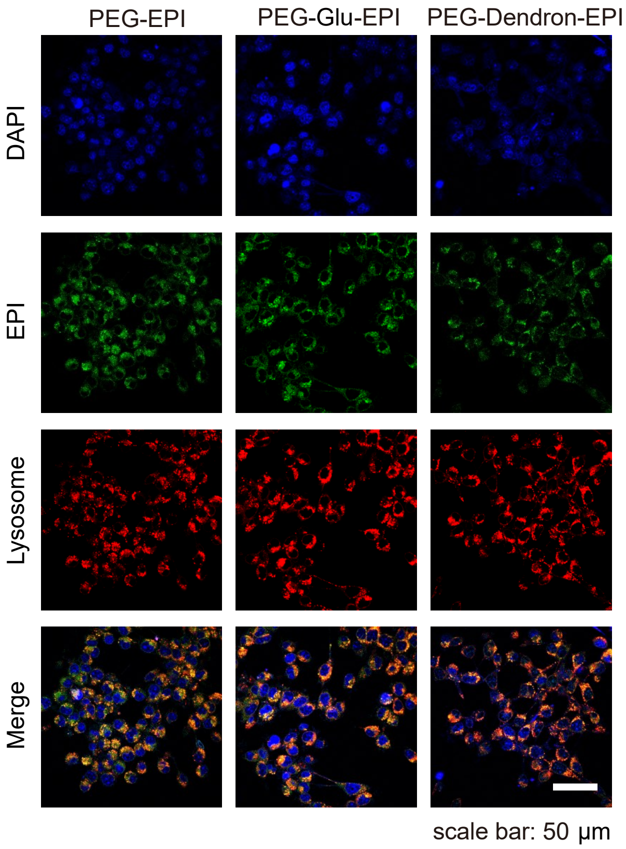

Figure S34. Confocal fluorescence microscopy images for cellular uptake of PEG-EPI, PEG-Glu-EPI, and PEG-Dendron-EPI at 6 h post treatment (EPI incubation concentration: 10 μg/mL, blue: DAPI, green: EPI, red: Lysosome). The average fluorescence intensities of the three prodrug polymers in the EPI channel are as follows: 30.396, 27.205, 21.325.

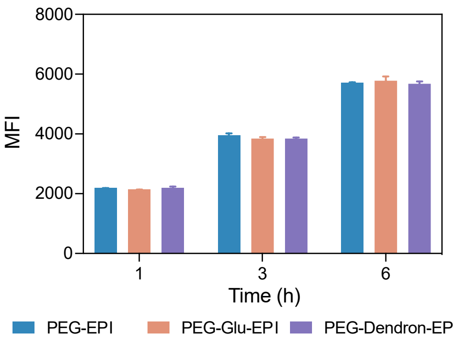

Figure S35. Mean fluorescence intensity (MFI) of cells after cellular uptake of PEG-EPI, PEG-Glu-EPI, and PEG-Dendron-EPI at 1 h, 3 h, and 6 h (n = 3) via flow cytometry analysis

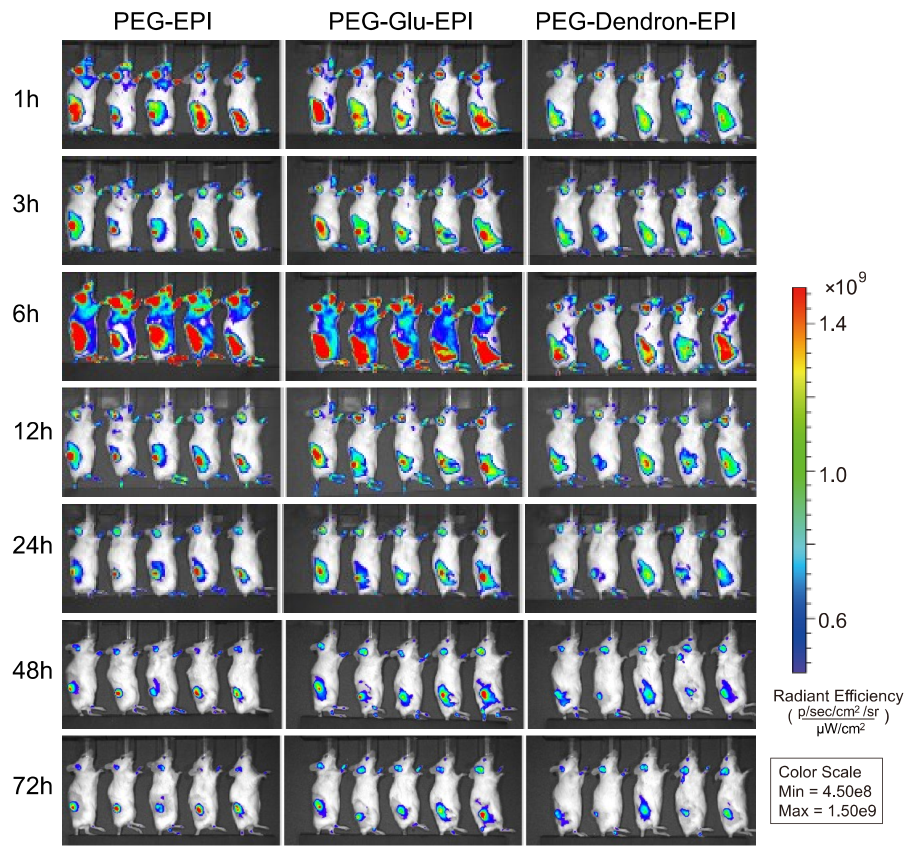

Figure S36. In vivo fluorescence images of mice at 1 h, 3 h, 6 h, 12 h, 24 h, 48 h, and 72 h post administration of PEG-EPI, PEG-Glu-EPI, and PEG-Dendron-EPI that were labeled with a fluorescent dye Cy5.

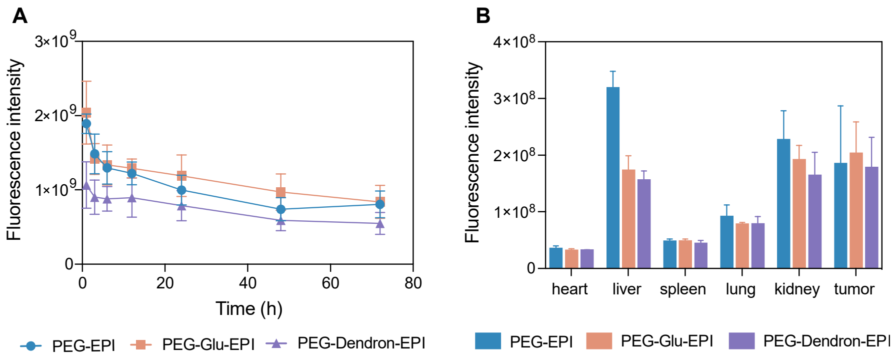

Figure S37. (A) The Cy5 fluorescence intensity at the tumor site at different time points after intravenous injection of Cy5-labeled nanoparticles based on PEG-EPI, PEG-Glu-EPI, and PEG-Dendron-EPI. (B) Ex vivo fluorescence intensities of major organs and tumors after they were collected at 72 h upon completion of in vivo monitoring.

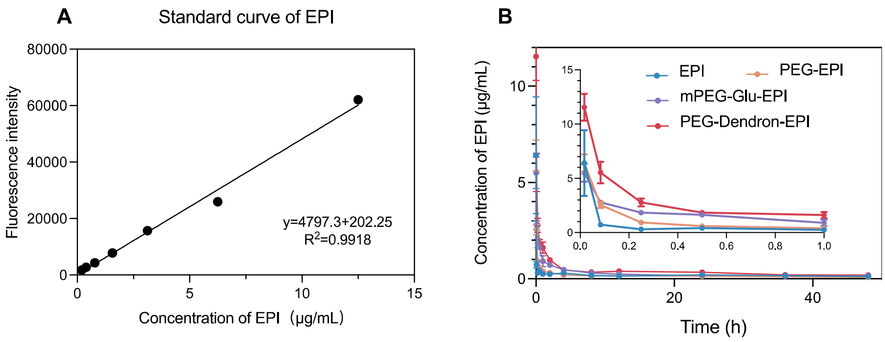

Figure S38. (A) The standard curve of EPI dissolved in DMSO (Ex: 485 nm, Em: 595 nm). (B) Pharmacokinetic analysis of PEG-EPI, PEG-Glu-EPI, and PEG-Dendron-EPI (n = 5).

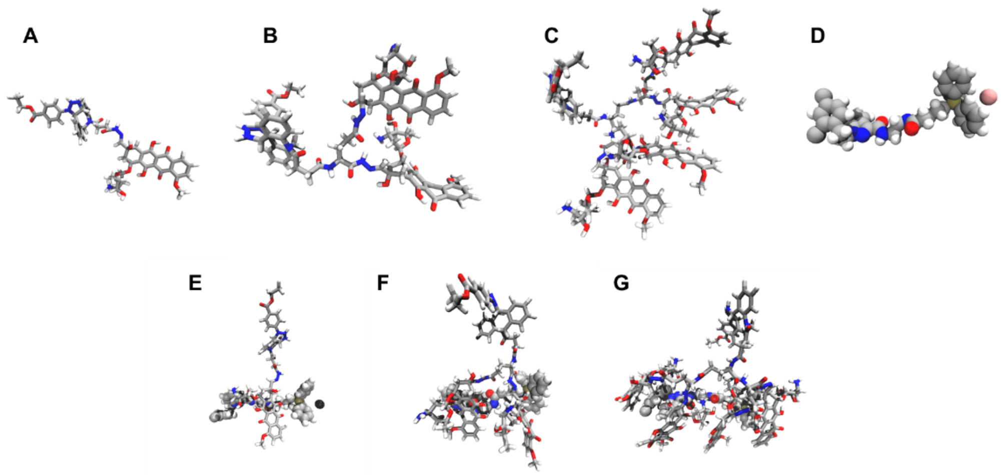

Figure S39. Structural features of prodrugs revealed from all-atom molecular dynamics (AAMD) simulations: (A) PEG-EPI, (B) PEG-Glu-EPI, and (C) PEG-Dendron-EPI terminal segments. (D) Schematic structure of the drug molecule TPP-LND. The corresponding drug adsorption models at the polymer terminals for (E) PEG-EPI, (F) PEG-Glu-EPI, and (G) PEG-Dendron-EPI. Color scheme: hydrogen (white), carbon (grey), oxygen (red), nitrogen (blue), and phosphorus (purple).

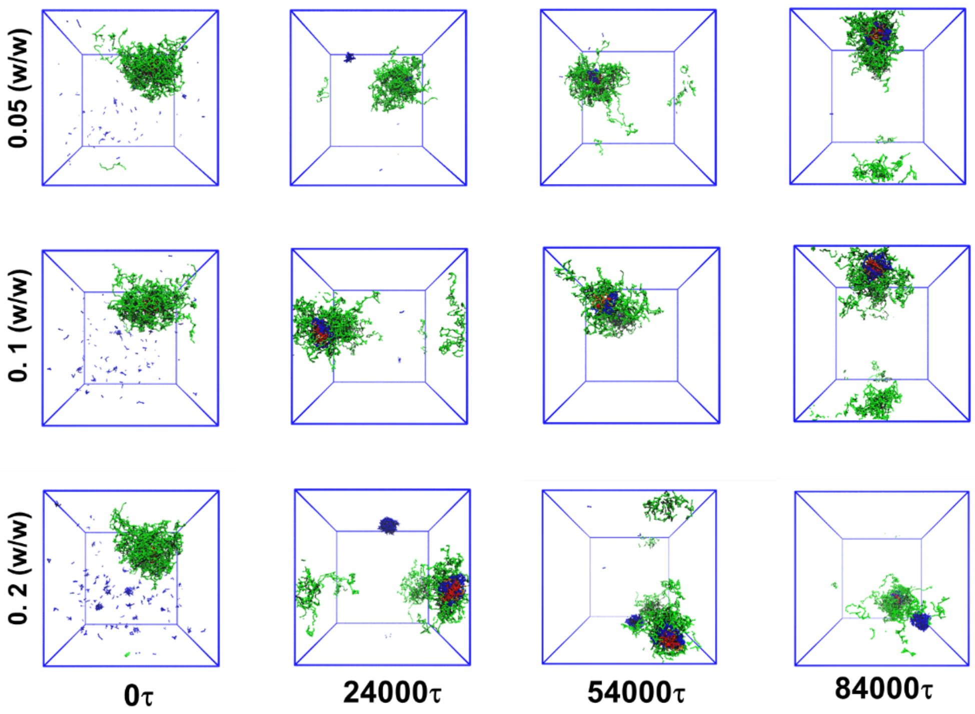

Figure S40. Simulation of nanoparticle formation by PEG-EPI and TPP-LND (blue beads) loading in water. The mass ratios of TPP-LND and PEG-EPI were 0.05, 0.1 and 0.2, respectively. The DPD simulations were performed for 84000τ with an integration time step of Δt = 0.01τ.

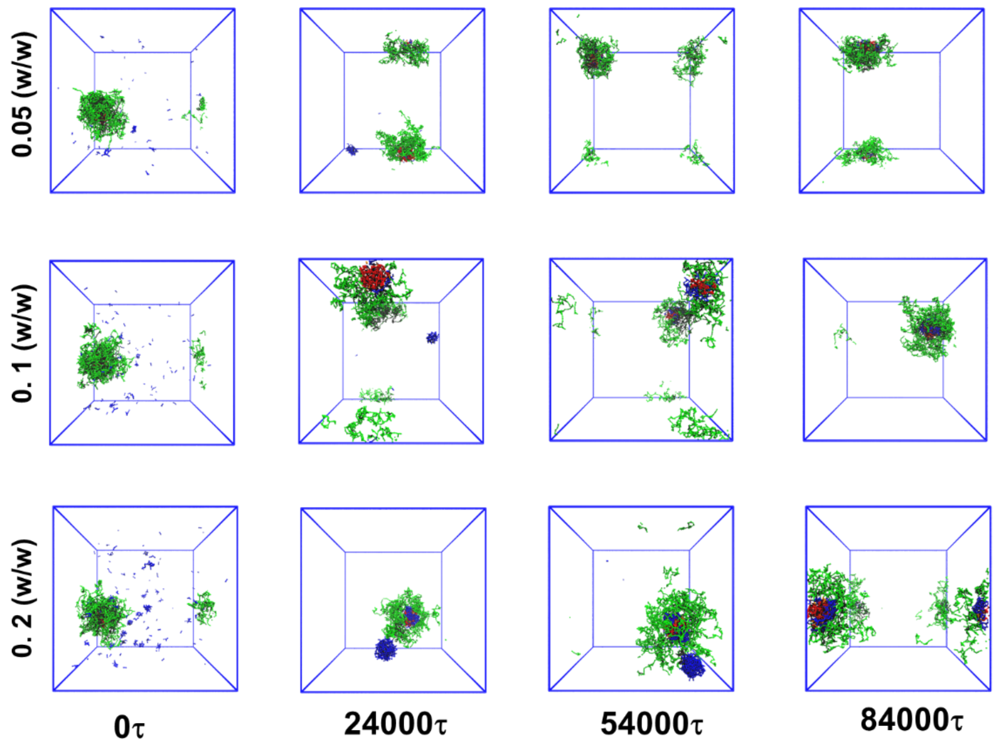

Figure S41. Simulation of nanoparticle formation by PEG-Glu-EPI and TPP-LND (blue beads) loading in water. The mass ratios of TPP-LND and PEG-Glu-EPI were 0.05, 0.1 and 0.2, respectively. The DPD simulations were performed for 84000τ with an integration time step of Δt = 0.01τ.

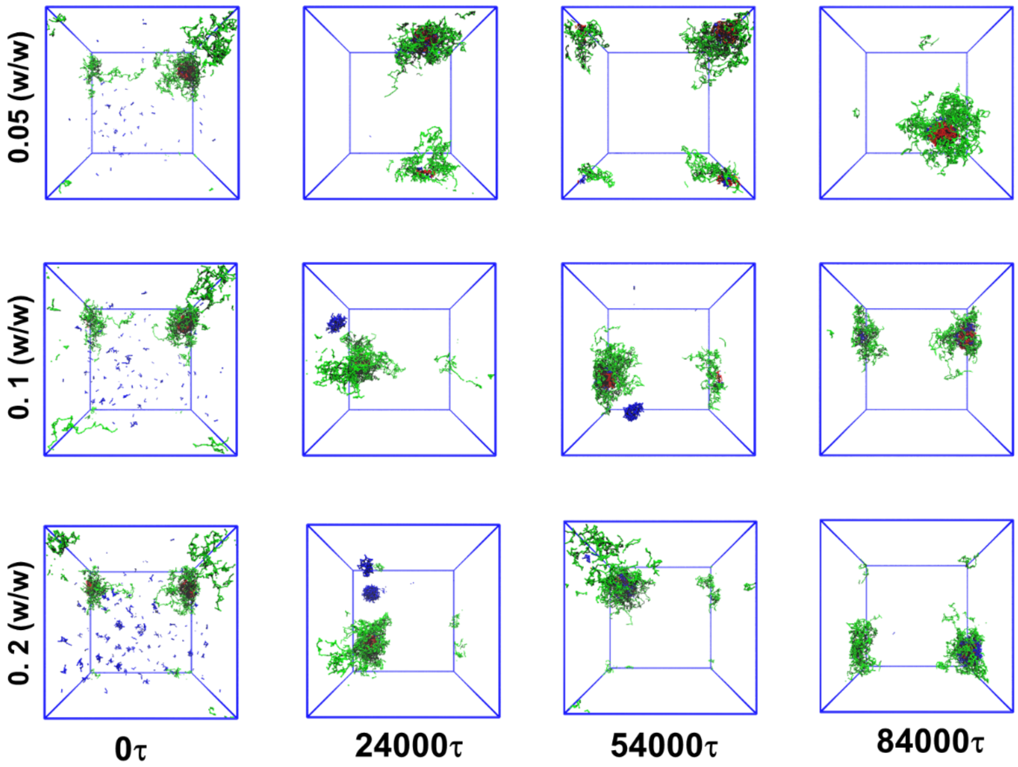

Figure S42. Simulation of nanoparticle formation by PEG-Dendron-EPI and TPP-LND (blue beads) loading in water. The mass ratios of TPP-LND and PEG-Dendron-EPI were 0.05, 0.1 and 0.2, respectively. The DPD simulations were performed for 84000τ with an integration time step of Δt = 0.01τ.

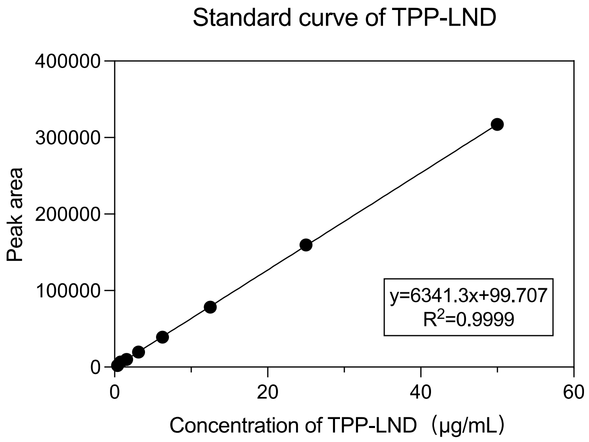

Figure S43. The standard curve of TPP-LND dissolved in 95% ethanol (detection wavelength: 298 nm).

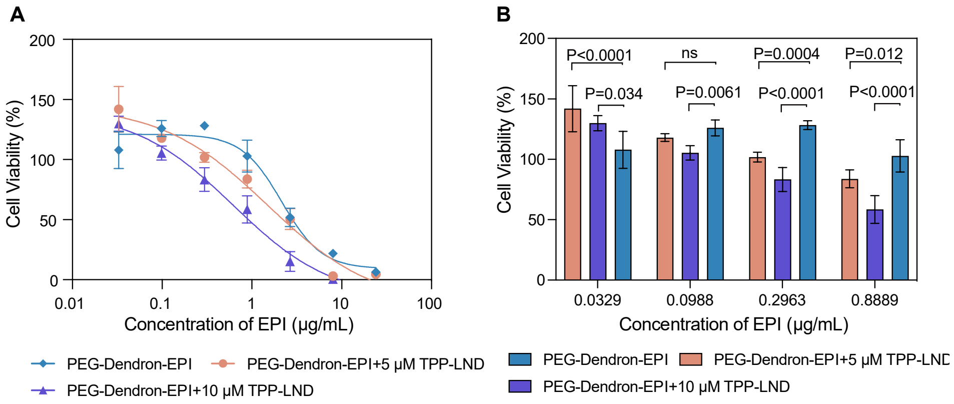

Figure S44. (A) The viability of 4T1 cells treated with PEG-Dendron-EPI, PEG-Dendron-EPI and TPP-LND at 5 μM, PEG-Dendron-EPI and TPP-LND at 10 μM at a gradient concentration of EPI in PEG-Dendron-EPI for 48 h. (B) Bar charts for treatment groups at selected EPI concentrations from (A) (n = 5).

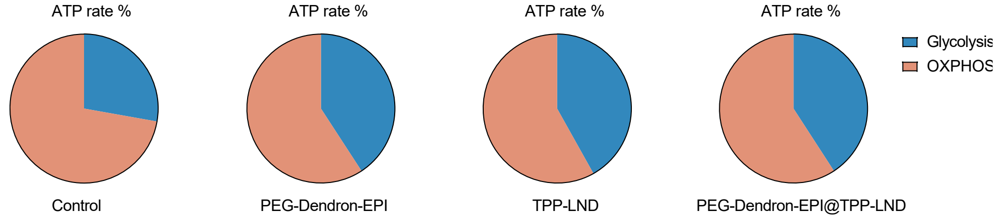

Figure S45. Percentages of cellular respiration via glycolysis and OXPHOS after 4T1 cells were treated with PEG-Dendron-EPI, TPP-LND, and PEG-Dendron-EPI @TPP-LND for 12 h.

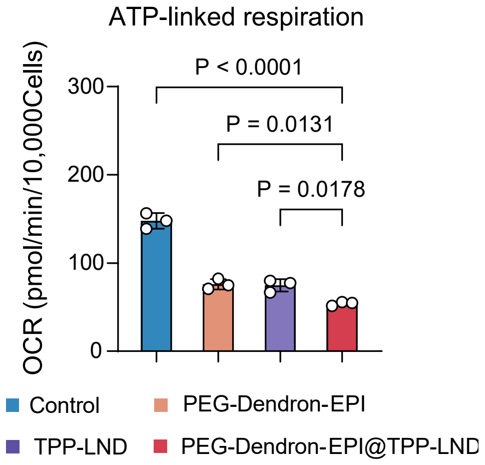

Figure S46. OCRs for ATP-linked respiration, indicating the proportion of basal respiration used for mitochondrial ATP production to meet cellular energy demands, in different treatment groups.

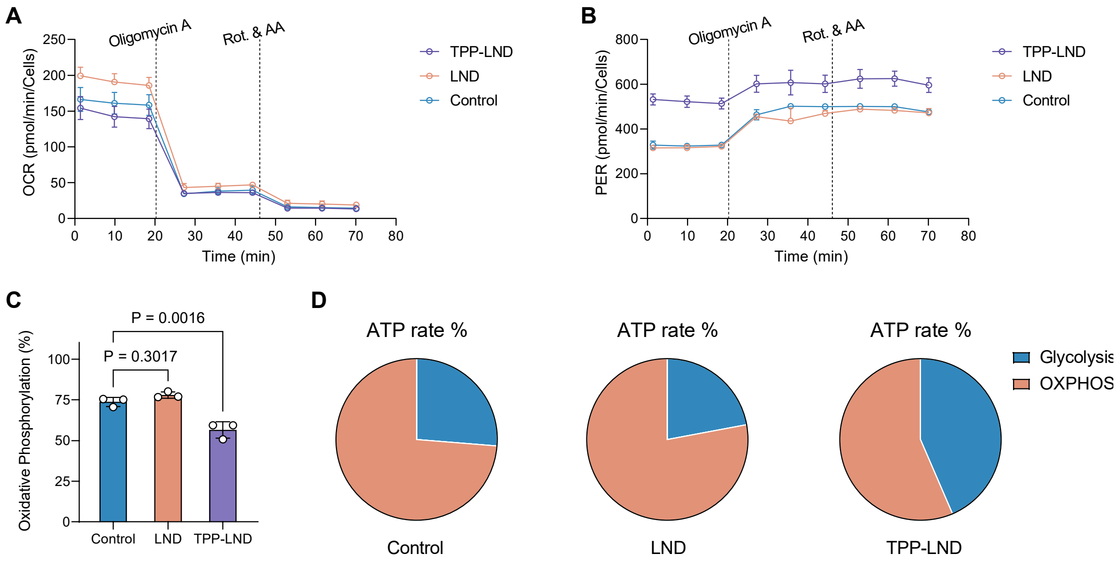

Figure S47. (A) Kinetic OCR curves derived from the real-time ATP rate assay in 4T1 cells treated with LND and TPP-LND versus the control cells. (B) The proton extrusion rate in 4T1 cells treated with LND and TPP-LND at different time points. (C) The degree of oxidative phosphorylation in 4T1 cells treated with LND and TPP-LND. (D) The proportion of ATP produced via glycolysis and oxidative phosphorylation in 4T1 cells treated with LND and TPP-LND versus the control group.

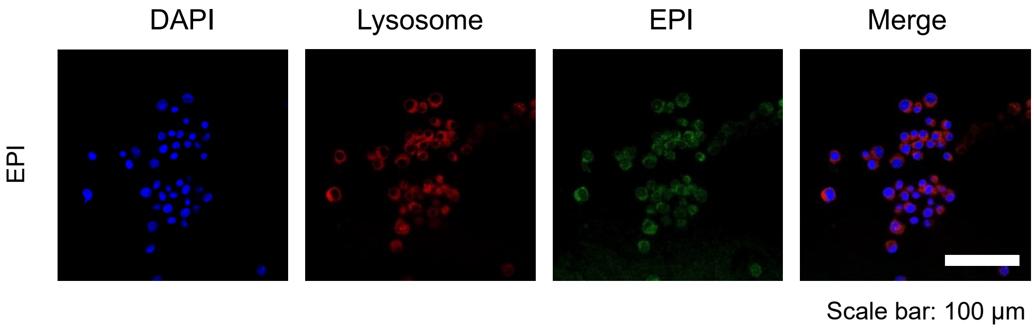

Figure S48. Confocal fluorescence microscopy images for localization of EPI within lysosomes of 4T1 cells after a 6-h treatment (EPI: 10 μg/mL). EPI: green; nuclei: blue; lysosomes: red; scale bar = 100 μm.

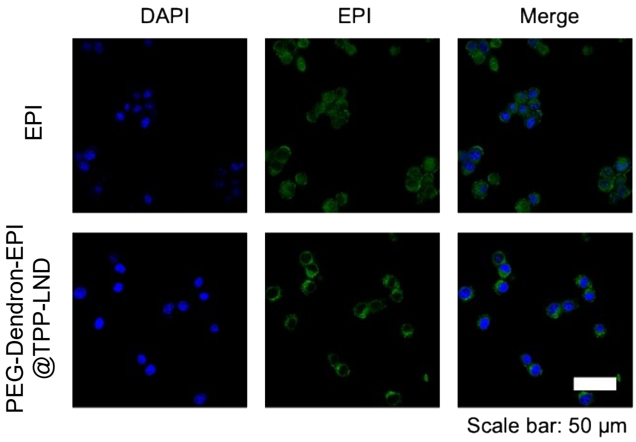

Figure S49. Cellular uptake images at 8 h post drug incubation (5 μg/mL) via a laser scanning confocal microscope.

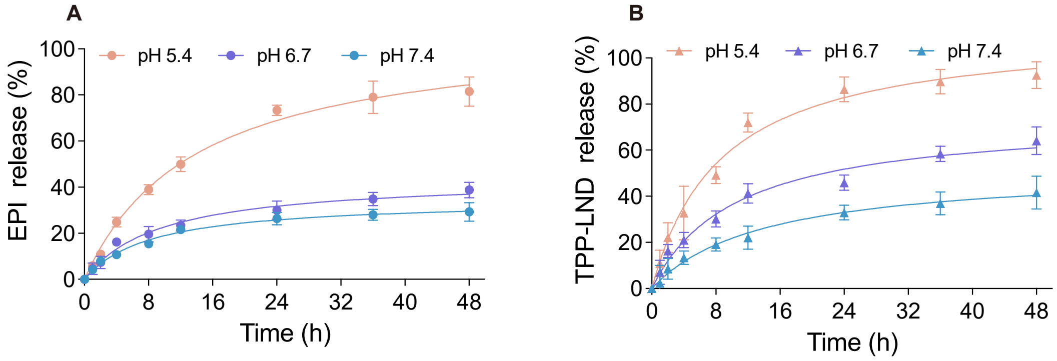

Figure S50. Release curves of (A) EPI and (B) TPP-LND from PEG-Dendron-EPI@TPP-LND under different pH conditions.

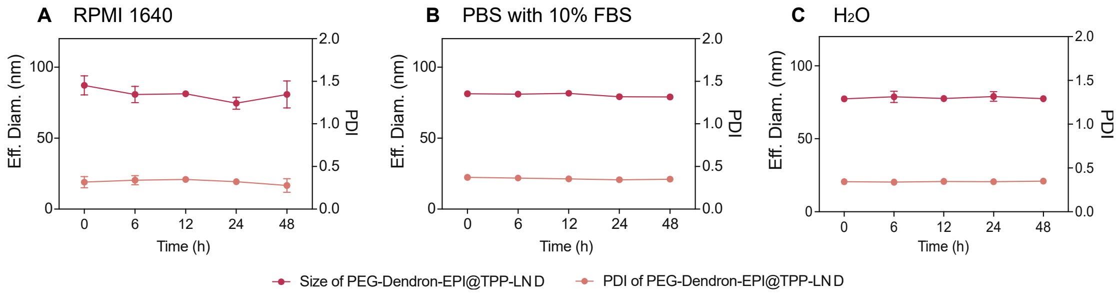

Figure S51. The stability of PEG-Dendron-EPI@TPP-LND in RPMI 1640 medium (A), PBS with 10% FBS (B) and H_2O (C) within 48 h.

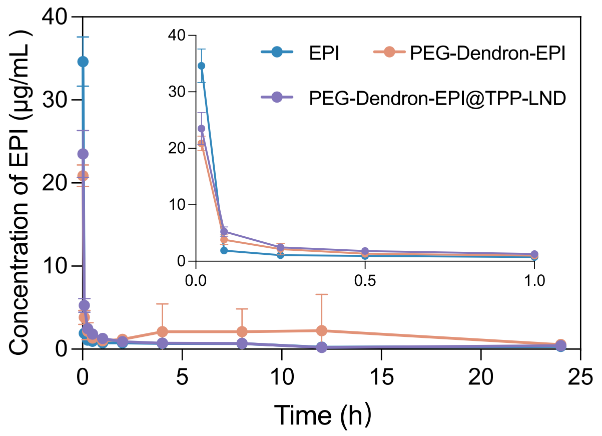

Figure S52. Pharmacokinetic analysis of EPI, PEG-Dendron-EPI, and PEG-Dendron-EPI@TPP-LND (n = 5).

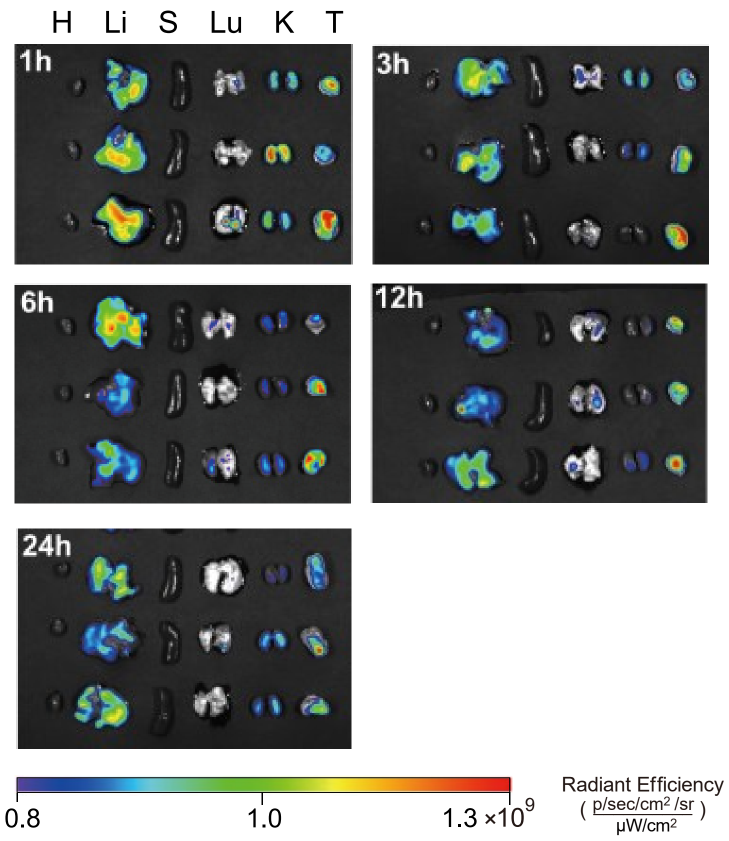

Figure S53. Ex vivo images of major organs at 1 h, 3 h, 6 h, 12 h, and 24 h after intravenous injection of 8 mg/kg PEG-Dendron-EPI into 4T1 tumor bearing mice. (H: heart, Li: liver, S: spleen, Lu: lung, K: kidney, and T: tumor)

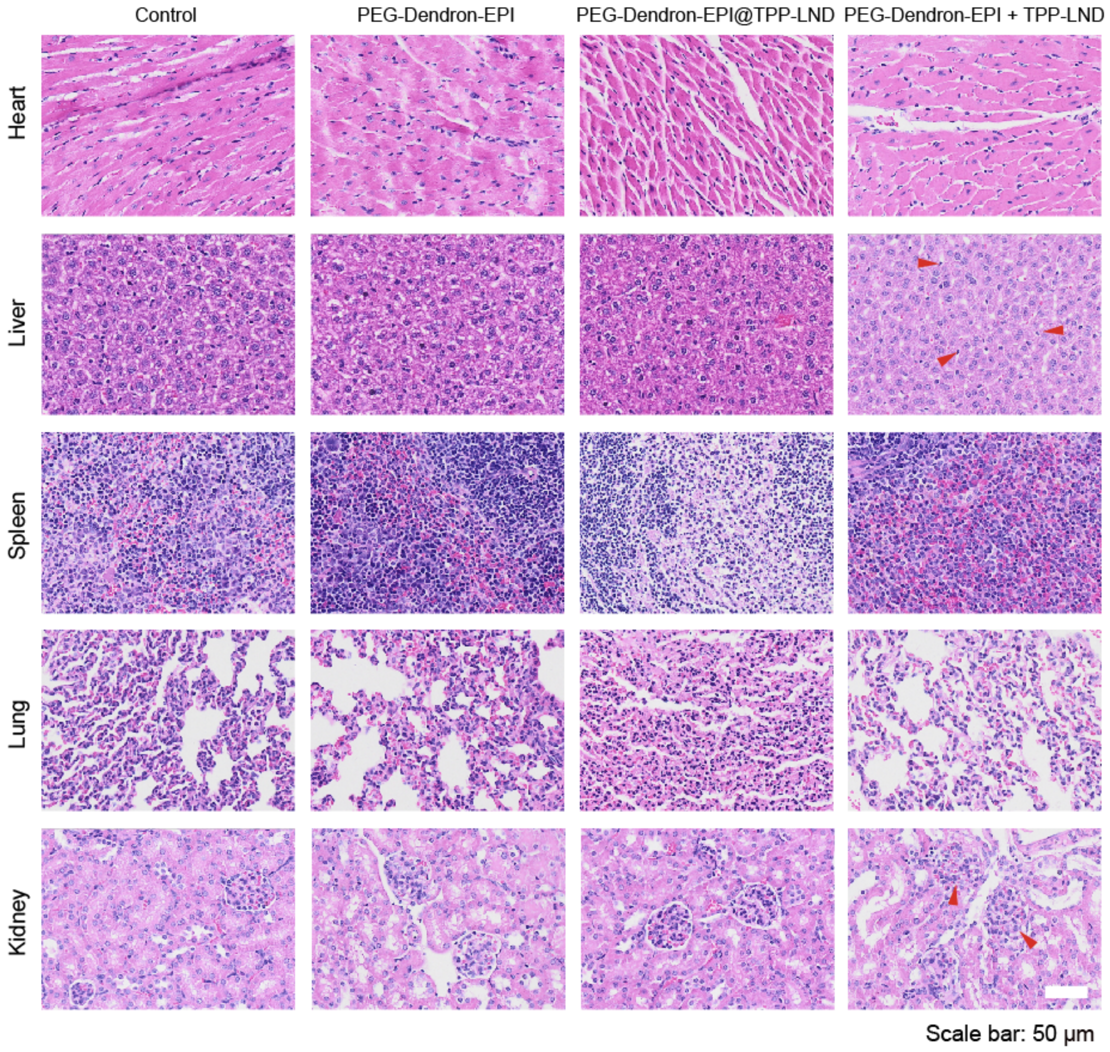

Figure S54. After the mice treated with different drugs were sacrificed on day 16 of treatment, the major organs were dissected and obtained. The results were photographed at 30× magnification after H&E staining. The red arrow points to the Kupffer cells in the liver and the glomerular inflammation in the kidney.

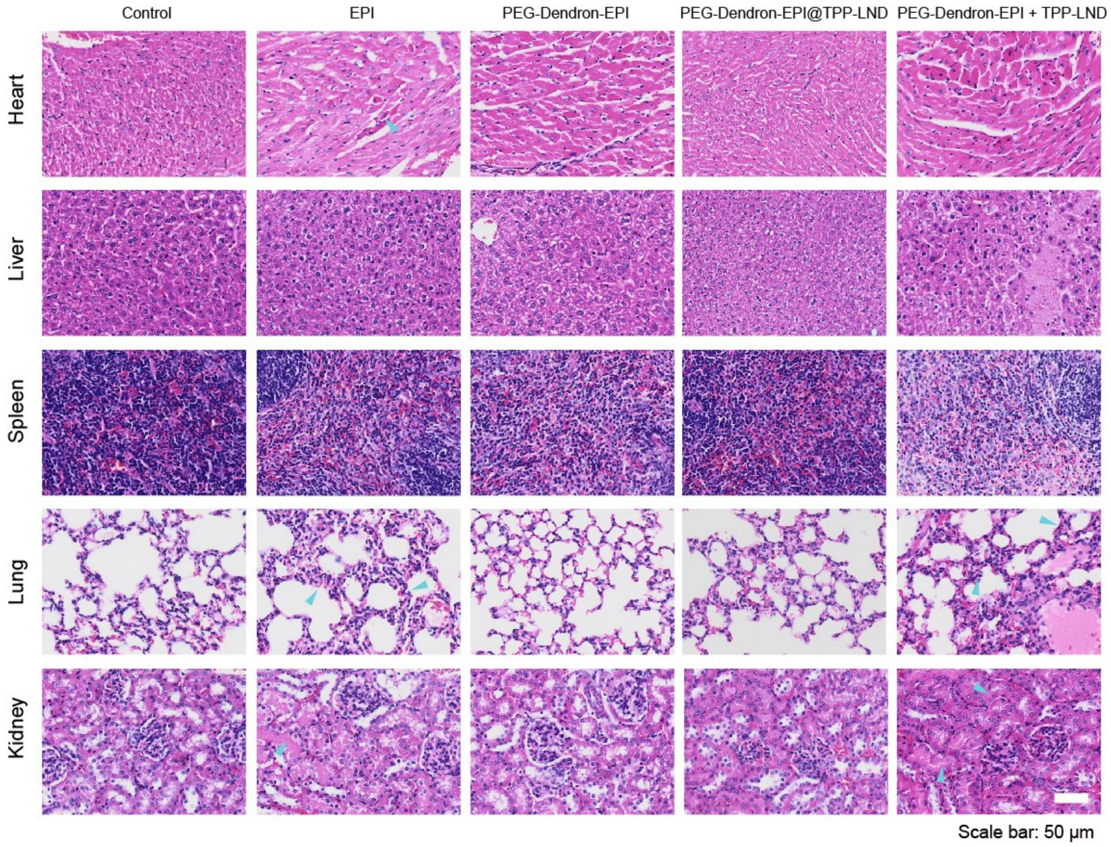

Figure S55. After the normal mice treated with different drugs were sacrificed on day 7 of treatment, the major organs were dissected and obtained. The results were photographed at 30× magnification after H&E staining. The light blue arrow points to the areas with obvious pathological abnormalities.

Table S1. The hydrodynamic size of prodrug polymers measured using a particle size analyzer via DLS (Dynamic Light Scattering), and the drug loading determined based on UV absorption (480 nm, n = 3).

Prodrug polymer	DLS size (nm)	Drug loading (%)
PEG-EPI	175.26 ± 4.87	8.8% ± 0.2
PEG-Glu-EPI	148.88 ± 3.32	11.5% ± 0.6
PEG-Dendron-EPI	122.62 ± 5.64	22.0% ± 0.7

Table S2. Pharmacokinetic data analysis results for PEG-EPI, PEG-Glu-EPI, and PEG-Dendron-EPI.

Parameter	PEG-EPI	PEG-Glu-EPI	PEG-Dendron-EPI
$AUC_{0\text{–}24}$(μg/mL·h)	5.053	8.749	13.016
$T_{1/2}$ (h)	13.375	14.257	8.874
CL (L/h/kg)	1.041	0.690	0.463
$MRT_{0\text{–}24}$(h)	20.420	16.289	15.220

Tabel S3. Energies of optimized structures using the Materials Studios.

	E^a/Kcal·mol^{-1}	ΔE^a/Kcal·mol^{-1}
TPP-LND	81.04	*b*
PEG-EPI	42.77	*b*
PEG-Glu-EPI	-59.11	*b*
PEG-Dendron-EPI	-271.68	*b*
PEG-EPI@TPP-LND	25.51	-98.30
PEG-Glu-EPI@TPP-LND	-79.73	-101.66
PEG-Dendron-EPI@TPP-LND	-371.5	-181.86

[a]The adsorption energy ΔE is calculated from $\Delta E = E_{total} - E_{adsorbate} - E_{adsorbent}$. [b]Not suitable.

Table S4. Encapsulation efficiency and drug loading capacity of PEG-EPI and TPP-LND at different ratios.

PEG-EPI: TPP-LND	Encapsulation rate (%)	Drug loading (%)
1:0.25	79.6	1.8
1:0.5	80.5	3.6
1:1	58.5	5.4
1:1.5	49.1	6.5
1:2	47.5	8.6

Table S5. Encapsulation efficiency and drug loading capacity of PEG-Glu-EPI and TPP-LND at different ratios.

PEG-Glu-EPI: TPP-LND	Encapsulation rate (%)	Drug loading (%)
1:0.25	63.3	1.8
1:0.5	58.5	3.4
1:1	44.2	5.1
1:1.5	35.2	6.1
1:2	32.4	7.5

Table S6. Encapsulation efficiency and drug loading capacity of PEG-Dendron-EPI and TPP-LND at different ratios.

PEG-Dendron-EPI: TPP-LND	Encapsulation rate (%)	Drug loading (%)
1:0.25	75.3	3.4
1:0.5	63.9	5.7
1:1	74.3	13.2
1:1.5	63.9	17.1
1:2	52.7	18.8

Table S7. The results of liver biochemical parameters measured in normal mice on day 7 following the third administration, according to the dosing frequency in Figure 7F (ALB: Albumin; ALT: Alanine Aminotransferase; AST: Aspartate Aminotransferase).

Biochemical indicators of liver	ALB (g/L)	ALT (U/L)	AST (U/L)
Control	40.63 ± 1.50	41.43 ± 4.32	156.43 ±34.01
EPI	37.53 ± 4.94	39.60 ± 2.27	135.03 ± 21.35
PEG-Dendron-EPI	40.7 ± 0.50	51.25 ± 3.97	197.95 ± 26.93
PEG-Dendron-EPI@TPP-LND	40.83 ± 1.26	42.30 ± 3.67	146.85 ± 21.41
PEG-Dendron-EPI+TPP-LND	40.83 ± 0.80	54.10 ± 8.27	200.83 ± 48.60

Table S8. The results of kidney biochemical indicators parameters measured in normal mice on day 7 following the third administration, according to the dosing frequency in Figure 7F (UA: Uric Acid; UR: Urea).

Biochemical indicators of kidney	UA (mg/dL)	UR (mg/dL)
Control	1.70 ± 0.42	35.88 ± 5.53
EPI	1.70 ± 0.37	25.48 ± 2.65
PEG-Dendron-EPI	2.00 ± 0.14	29.93 ± 2.88
PEG-Dendron-EPI@TPP-LND	2.13 ± 0.28	36.85 ± 4.64
PEG-Dendron-EPI+TPP-LND	2.28 ± 0.29	30.93 ± 8.22

References

1. Hoogerbrugge PJ, Koelman JMVA. Simulating Microscopic Hydrodynamic Phenomena with Dissipative Particle Dynamics. Europhys Lett (EPL). 1992; 19: 155-60.
2. Groot RD, Madden TJ. Dynamic simulation of diblock copolymer microphase separation. J Chem Phys. 1998; 108: 8713-24.
3. Groot RD, Warren PB. Dissipative particle dynamics: Bridging the gap between atomistic and mesoscopic simulation. J Chem Phys. 1997; 107: 4423-35.
4. Liu H, Xue Y-H, Zhu Y-L, Gu F-L, Lu Z-Y. Inverse Design of Molecular Weight Distribution in Controlled Polymerization via a One-Pot Reaction Strategy. Macromolecules. 2020; 53: 6409-19.
5. Liu H, Qian HJ, Zhao Y, Lu ZY. Dissipative particle dynamics simulation study on the binary mixture phase separation coupled with polymerization. J Chem Phys. 2007; 127: 144903.
6. Shamay Y, Shah J, Isik M, Mizrachi A, Leibold J, Tschaharganeh DF, et al. Quantitative self-assembly prediction yields targeted nanomedicines. Nat Mater. 2018; 17: 361-8.
7. Sun H, Ren P, Fried JR. The COMPASS force field: parameterization and validation for phosphazenes. Comput Theor Polym Sci. 1998; 8: 229-46.
8. Vasquez VR, Williams BC, Graeve OA. Stability and comparative analysis of AOT/water/isooctane reverse micelle system using dynamic light scattering and molecular dynamics. J Phys Chem B. 2011; 115: 2979-87.
9. Ma J, Xiao P, Wang P, Han X, Luo J, Shi R, et al. Molecular dynamics simulation study on π-π stacking of Gemini surfactants in oil/water systems. Chin J Chem Eng. 2022; 50: 335-46.
10. Ma J, Song X, Luo J, Zhao T, Yu H, Peng B, et al. Molecular Dynamics Simulation Insight into Interfacial Stability and Fluidity Properties of Microemulsions. Langmuir. 2019; 35: 13636-45.
11. Humphrey W, Dalke A, Schulten K. VMD: visual molecular dynamics. J Mol Graph. 1996; 14: 33-8, 27-8.
